# Marine Pharmacology in 2022–2023: Marine Compounds with Antibacterial, Antidiabetic, Antifungal, Anti-Inflammatory, Antiprotozoal, Antituberculosis and Antiviral Activities, Affecting the Immune and Nervous Systems, and Other Miscellaneous Mechanisms of Action

**DOI:** 10.3390/md24040133

**Published:** 2026-04-09

**Authors:** Alejandro M. S. Mayer, Veronica A. Mayer, Michelle Swanson-Mungerson, Marsha L. Pierce, Cai M. Roberts, Abimael D. Rodríguez, Fumiaki Nakamura, Orazio Taglialatela-Scafati

**Affiliations:** 1 Department of Pharmacology, College of Graduate Studies, Midwestern University, 555 31st Street, Downers Grove, IL 60515, USA; mpierc1@midwestern.edu (M.L.P.); crober@midwestern.edu (C.M.R.); 2 Department of Nursing Education, School of Nursing, Aurora University, 347 S. Gladstone Ave., Aurora, IL 60506, USA; vmayer@aurora.edu; 3 Department of Microbiology and Immunology, College of Graduate Studies, Midwestern University, 555 31st Street, Downers Grove, IL 60515, USA; mswans@midwestern.edu; 4 Molecular Sciences Research Center, University of Puerto Rico, 1390 Ponce de León Avenue, San Juan, PR 00926, USA; abimael.rodriguez1@upr.edu; 5 Research Institute for Science and Engineering, Waseda University, 3-4-1 Okubo, Shinjuku-ku, Tokyo 169-8555, Japan; what-will_be.x2@akane.waseda.jp; 6 Department of Pharmacy, University of Naples Federico II, Via D. Montesano 49, I-80131 Napoli, Italy; scatagli@unina.it

**Keywords:** drug, marine, sea, pharmacology, pharmaceutical, review, toxicology, pipeline, preclinical, mechanism

## Abstract

During 2022–2023, research groups from 40 nations contributed to the preclinical pharmacology of 173 structurally defined marine-derived compounds, unveiling innovative mechanisms of action. Peer-reviewed publications in the field of marine natural product pharmacology during 2022–2023 included mechanism-of-action studies with 43 compounds showing antibacterial, antifungal, antiprotozoal, antitubercular, and antiviral activity. Additional mechanism-of-action studies were reported for 74 marine compounds that exhibited antidiabetic and anti-inflammatory properties, as well as significant effects on both the immune and nervous systems. Finally, while 65 marine compounds revealed unique and diverse pharmacological mechanisms, further investigation will be required to determine whether they will contribute to a particular therapeutic category. Collectively, the pharmacology of 2022–2023 preclinical marine natural products demonstrated robust activity, offering both novel mechanistic insights and promising chemical scaffolds to enrich the 2026 marine pharmaceutical development pipeline (https://www.marinepharmacology.org/) which currently consists of 17 marine-derived pharmaceuticals approved for clinical use and 29 compounds in either Phase I, II or III of clinical pharmaceutical development.

## 1. Introduction

**Figure 1:**
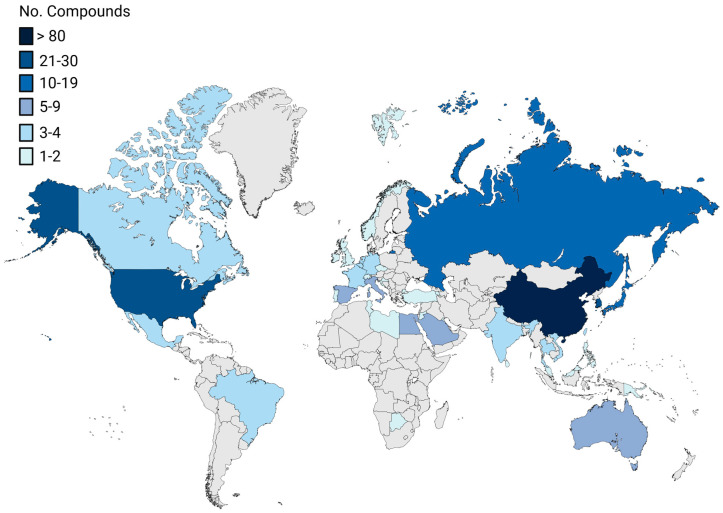
Marine pharmacology in 2022–2023: World heatmap illustration of the geographic distribution of marine compounds studied in this review. Color gradients represent number of compounds, with lighter shades indicating lower numbers and darker shades indicating higher numbers. Regions not indicated are shown in gray. Created in BioRender. Pierce, M. (2026) https://BioRender.com/5p2l8so.

**Figure 2:**
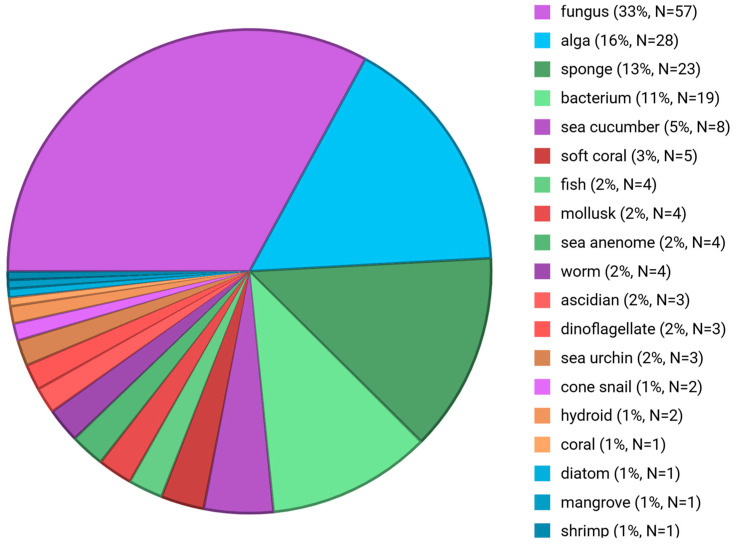
Marine pharmacology in 2022–2023: Proportions of compounds from source organisms included in this review. Each segment represents a distinct organism, with segment size corresponding to its percentage contribution to the total number of studied compounds. Created in BioRender. Pierce, M. (2026) https://BioRender.com/5p2l8so.

**Figure 3:**
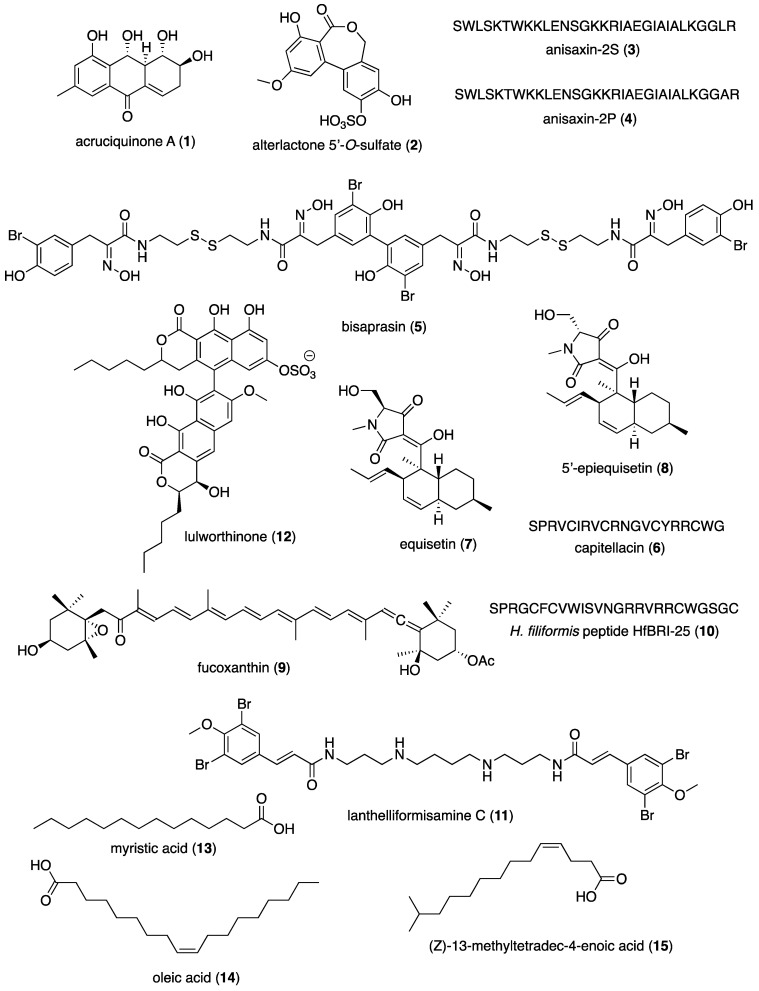

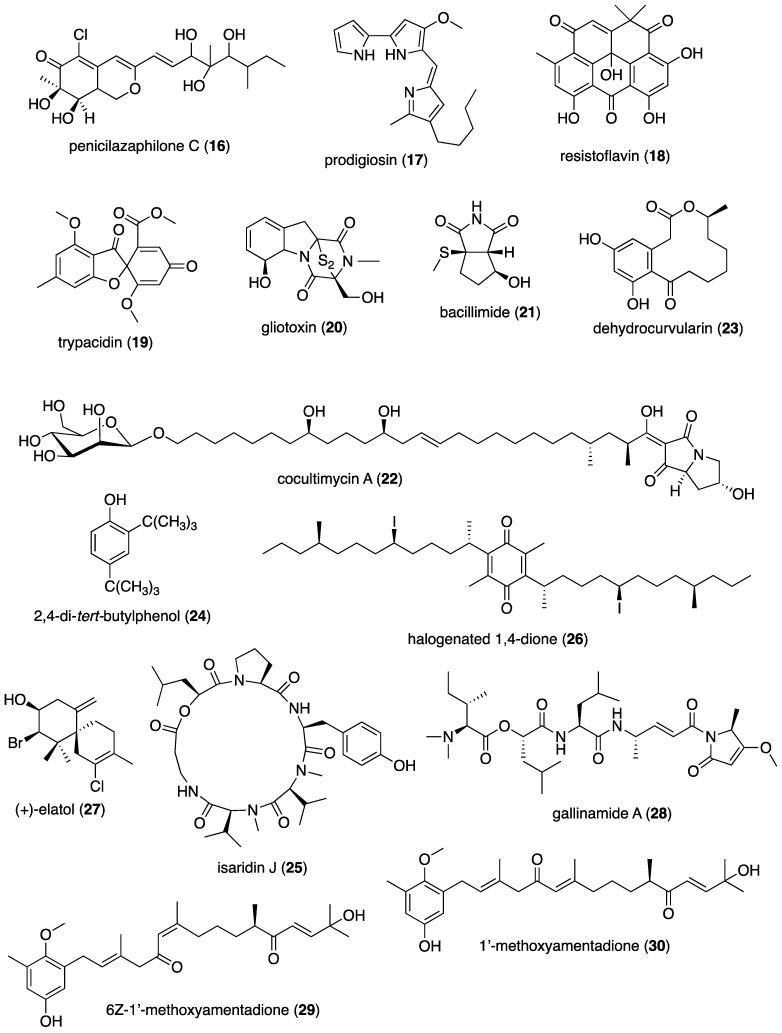

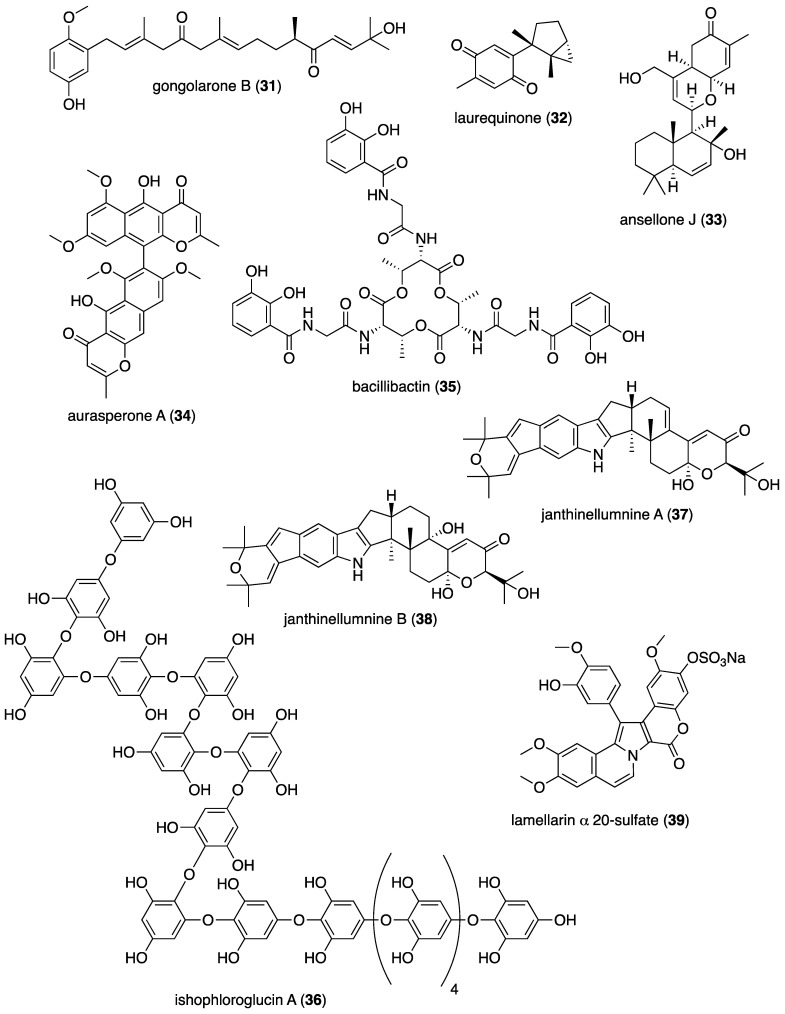

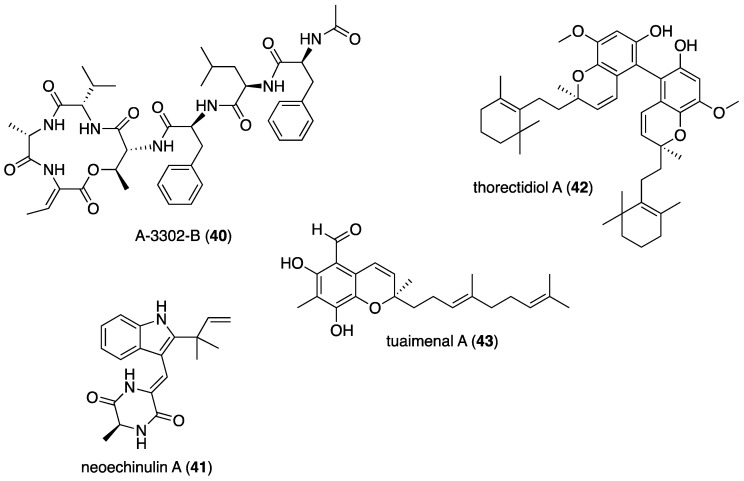
Marine pharmacology in 2022–2023: marine compounds with antibacterial, antituberculosis, antifungal, antiprotozoal, and antiviral activities.

**Figure 4:**
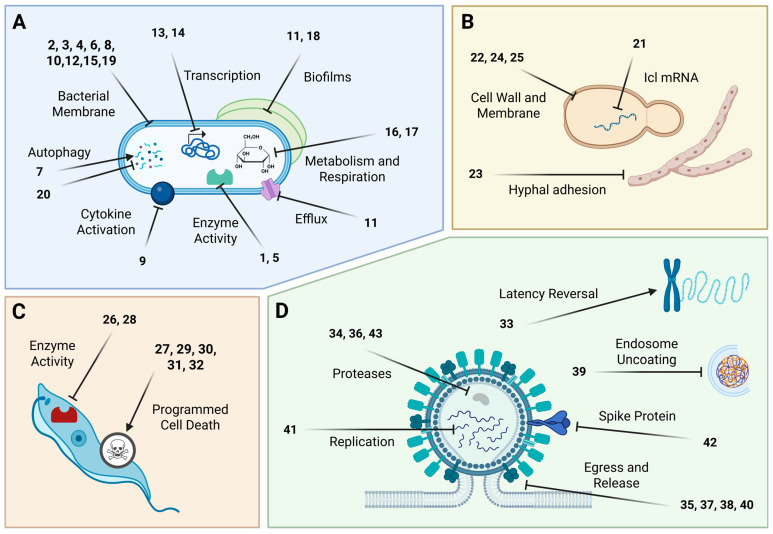
Marine pharmacology in 2022–2023: Mechanisms and pathways for marine compounds in Table 1. Compound numbers as seen in Table 1 and Figure 3 are grouped by mechanism for (**A**) antibacterial and antituberculosis, (**B**) antifungal, (**C**) antiprotozoal, and (**D**) antiviral agents. Created in BioRender. Roberts, C. (2026), https://BioRender.com/xiz3i4p.

**Figure 5:**
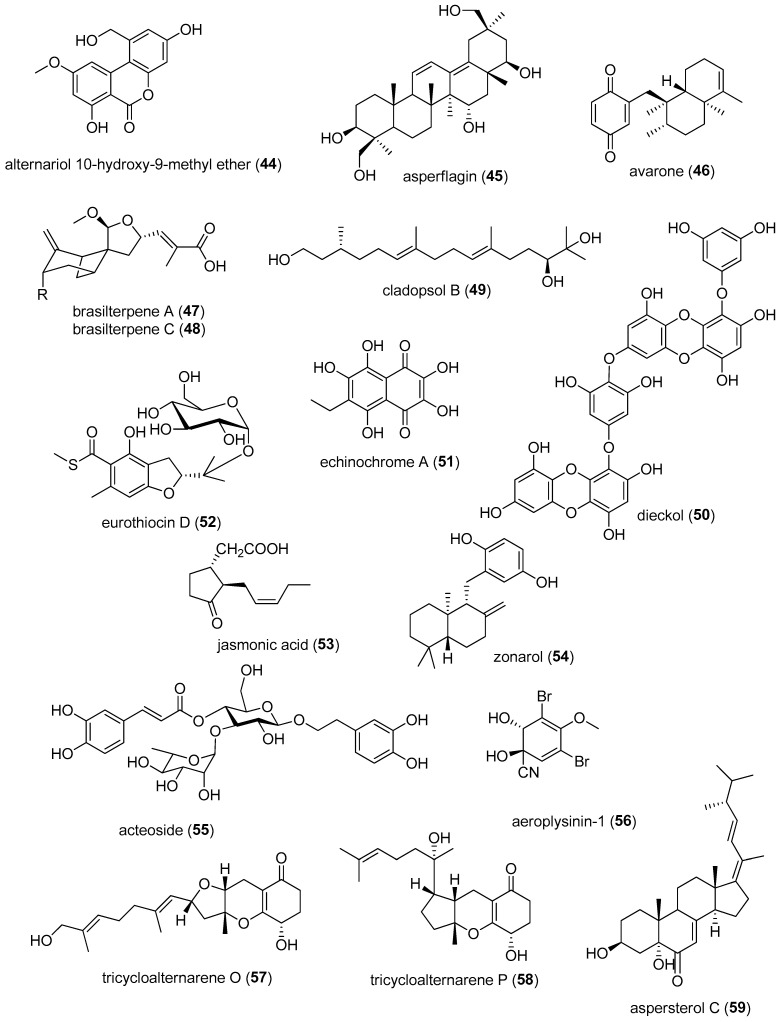

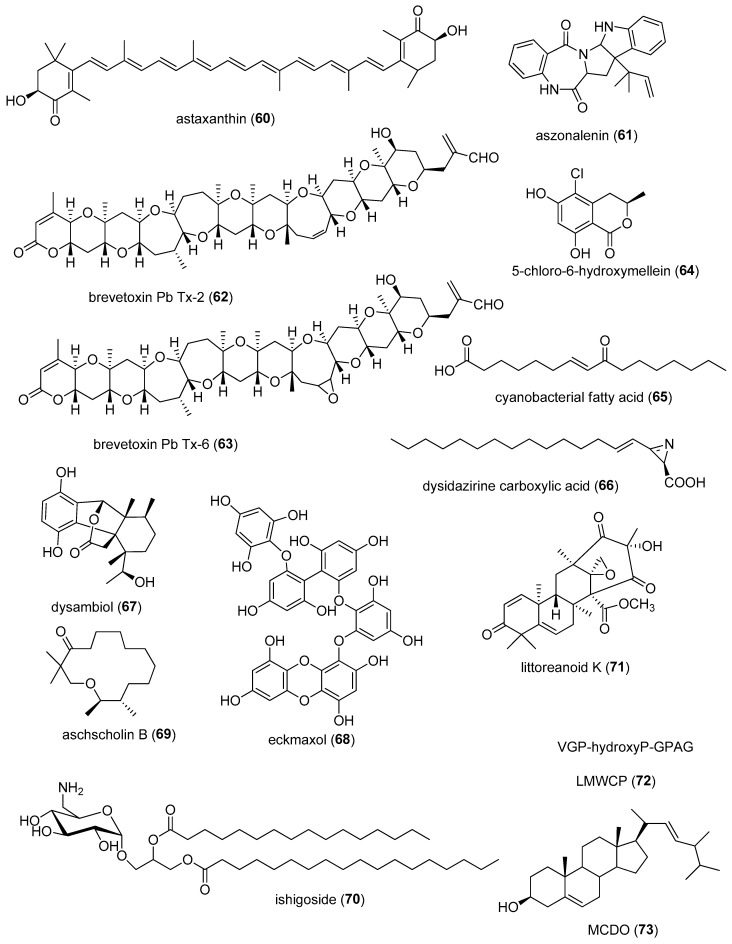

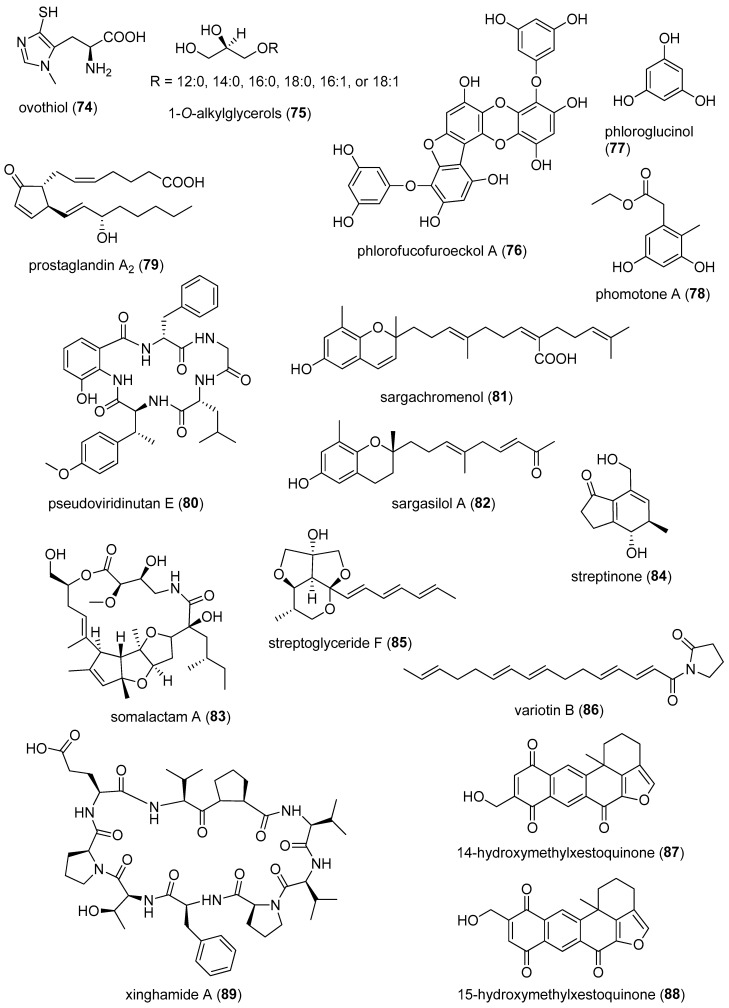

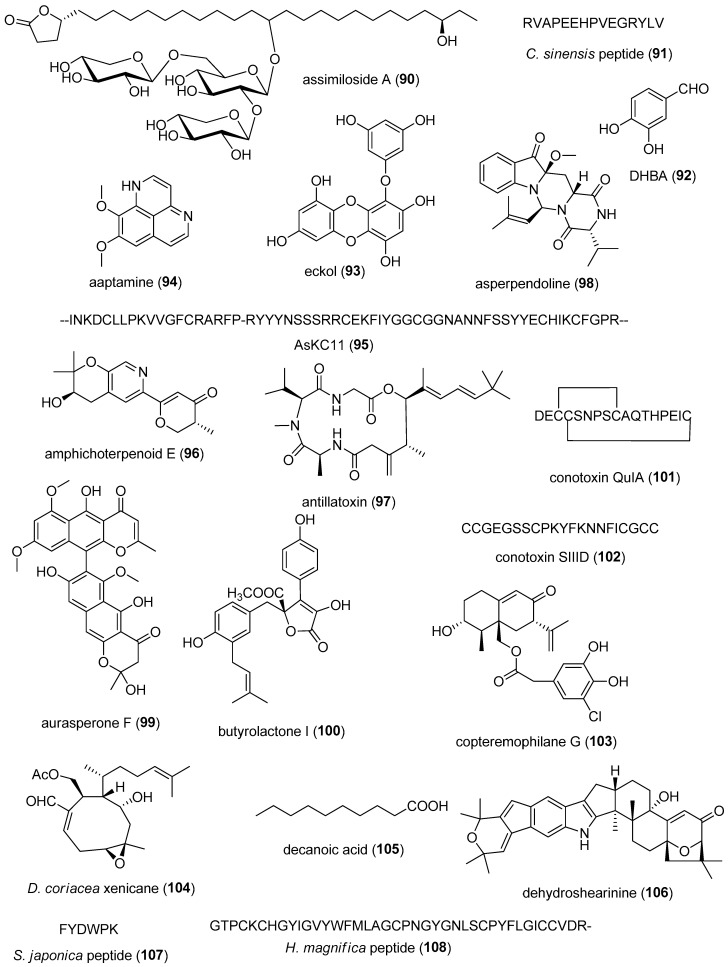

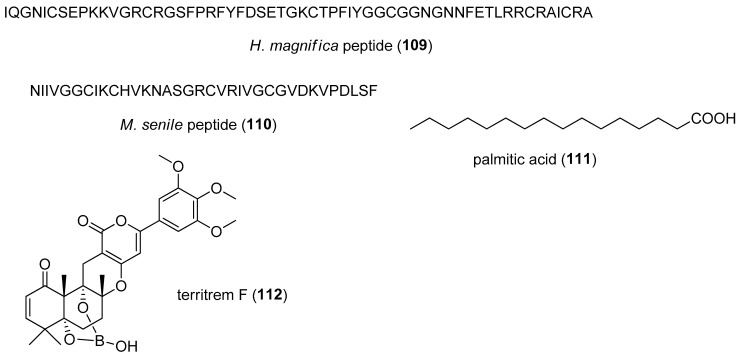
Marine pharmacology in 2022–2023: marine compounds with antidiabetic and anti-inflammatory activity and affecting the immune and nervous systems.

**Figure 6:**
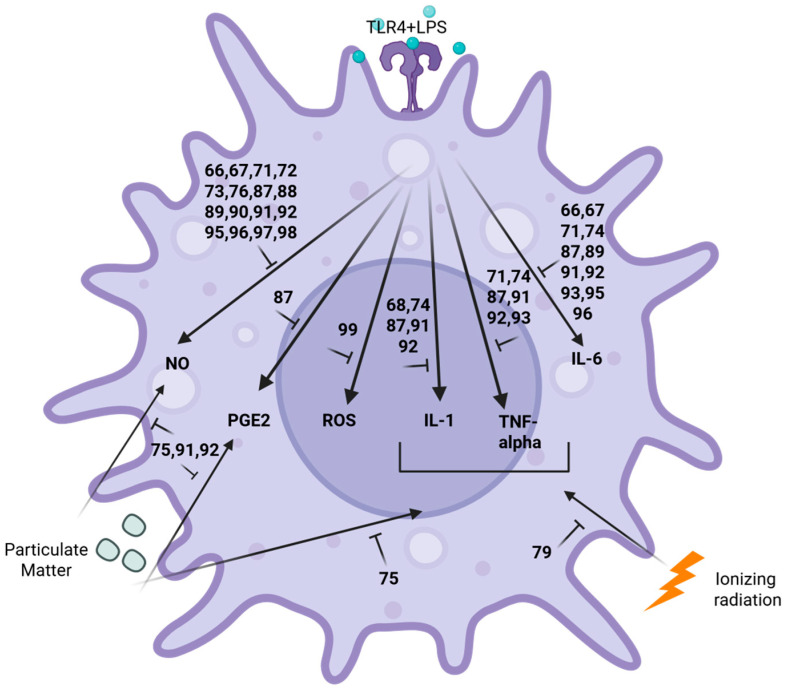
Marine pharmacology in 2022–2023: Mechanisms and pathways for anti-inflammatory marine compounds in Table 2. Compound numbers as seen in Table 2 are grouped by anti-inflammatory mechanisms on macrophages activated either by LPS, particulate matter or ionizing radiation. Created in Biorender. Swanson-Mungerson, M. (2026), https://BioRender.com/me83j4o.

**Figure 7:**
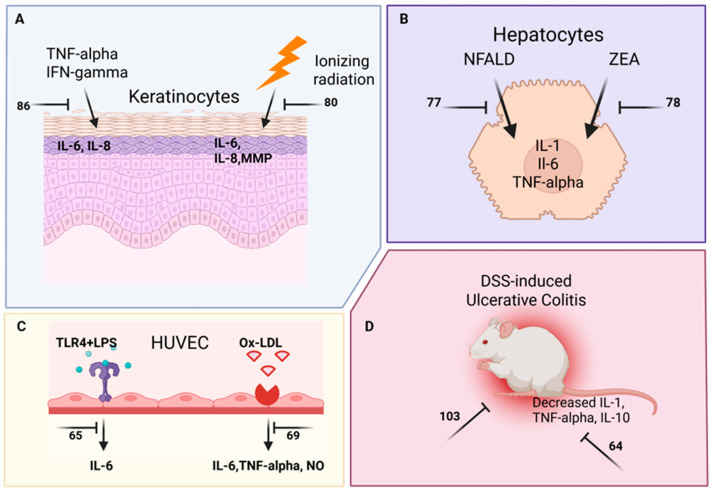
Marine pharmacology in 2022–2023: Mechanisms and pathways for anti-inflammatory marine compounds in Table 2. Compound numbers as seen in Table 2 and Figure 5 are grouped by cell type in (**A**) keratinocytes activated by either TNF-alpha and IFN-gamma or ionizing radiation, (**B**) hepatocytes in NFALD model or exposed to ZEA, (**C**) HUVECs activated by either LPS or Ox-LDL, and (**D**) DSS-induced ulcerative colitis in vivo. Created in Biorender. Swanson-Mungerson, M. (2026), https://BioRender.com/me83j4o.

**Figure 8:**
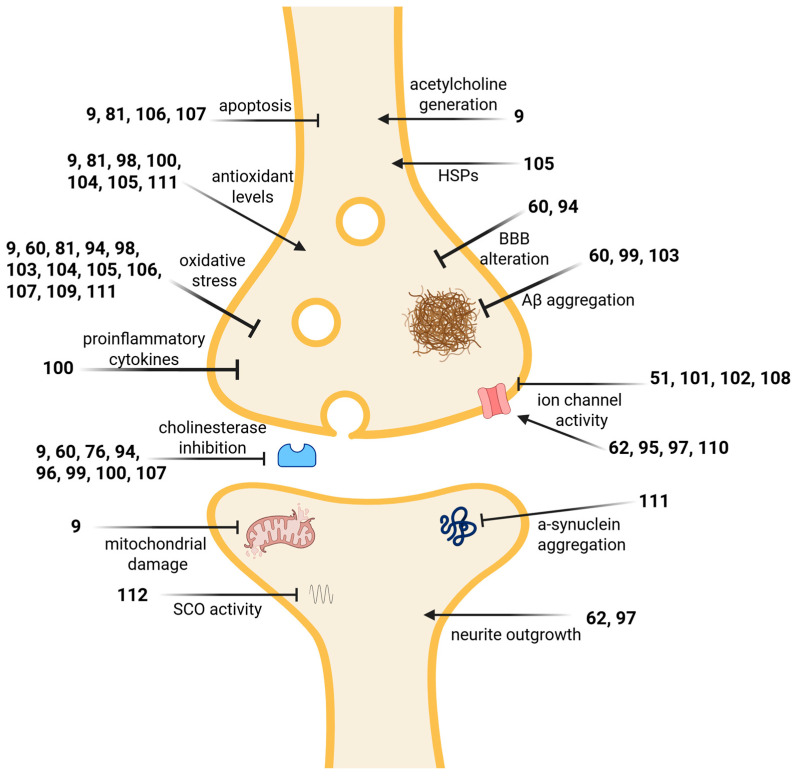
Marine pharmacology in 2022–2023: Mechanisms of marine compounds affecting the nervous system in Table 2. Compound numbers as seen in Table 2 are grouped by effects on the nervous system. Aβ, amyloid beta; BBB, blood–brain barrier; HSPs, heat shock proteins; SCO, spontaneous calcium oscillations. Created in BioRender. Pierce, M. (2026), https://BioRender.com/731qvfi.

**Figure 9:**
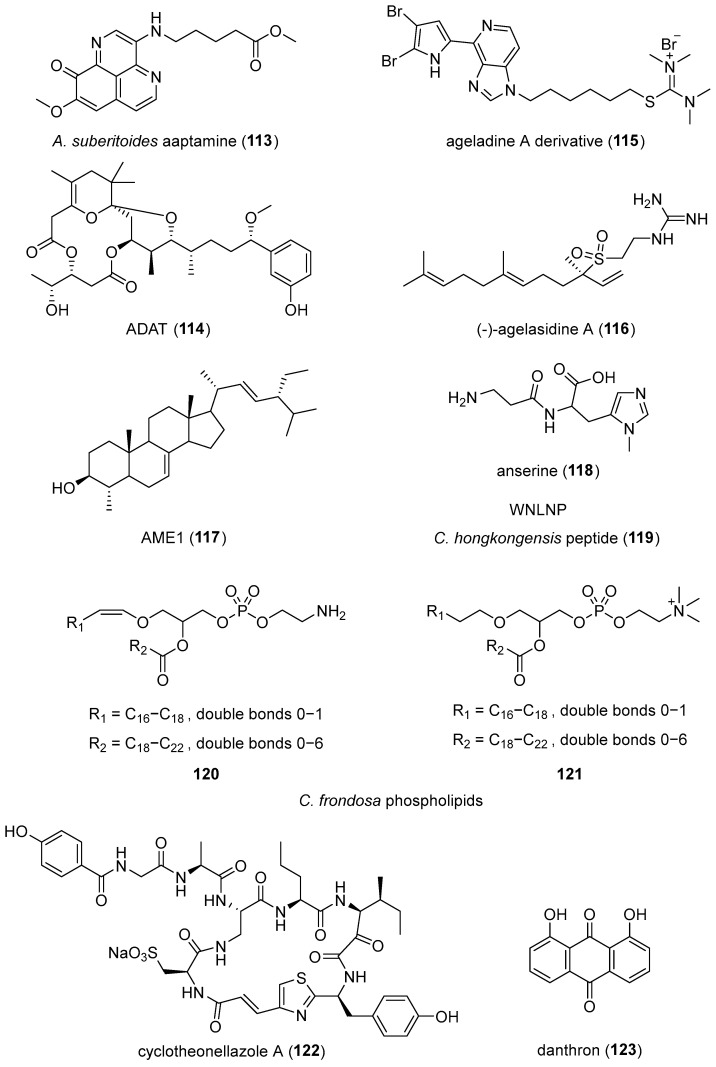

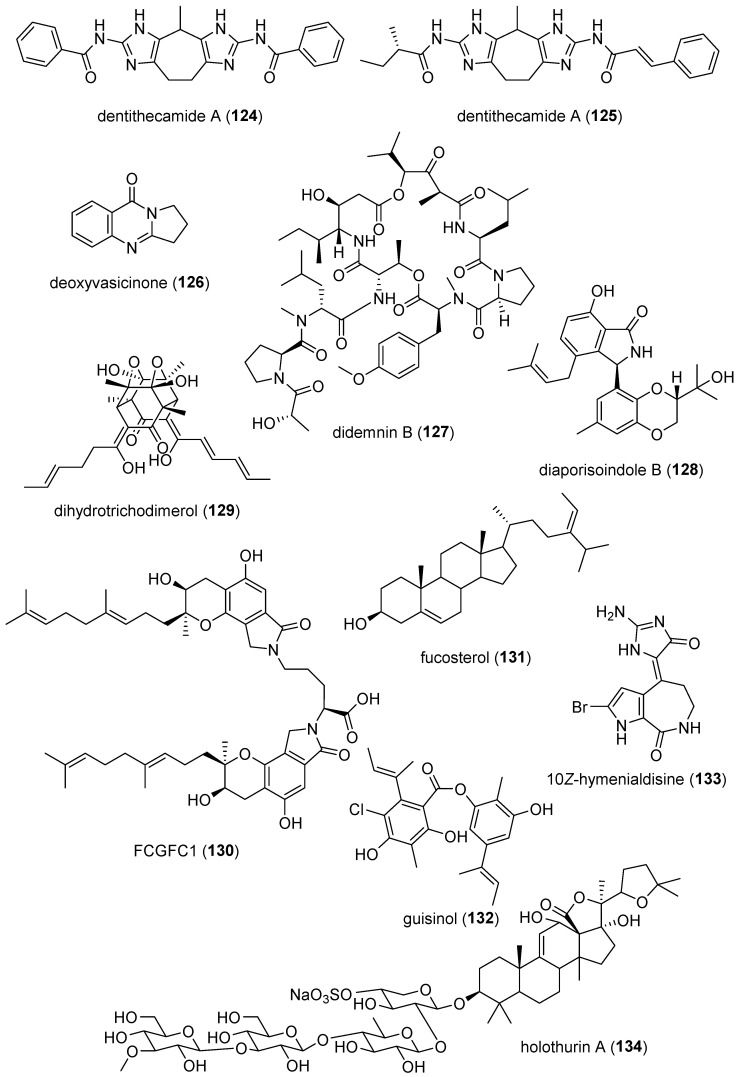

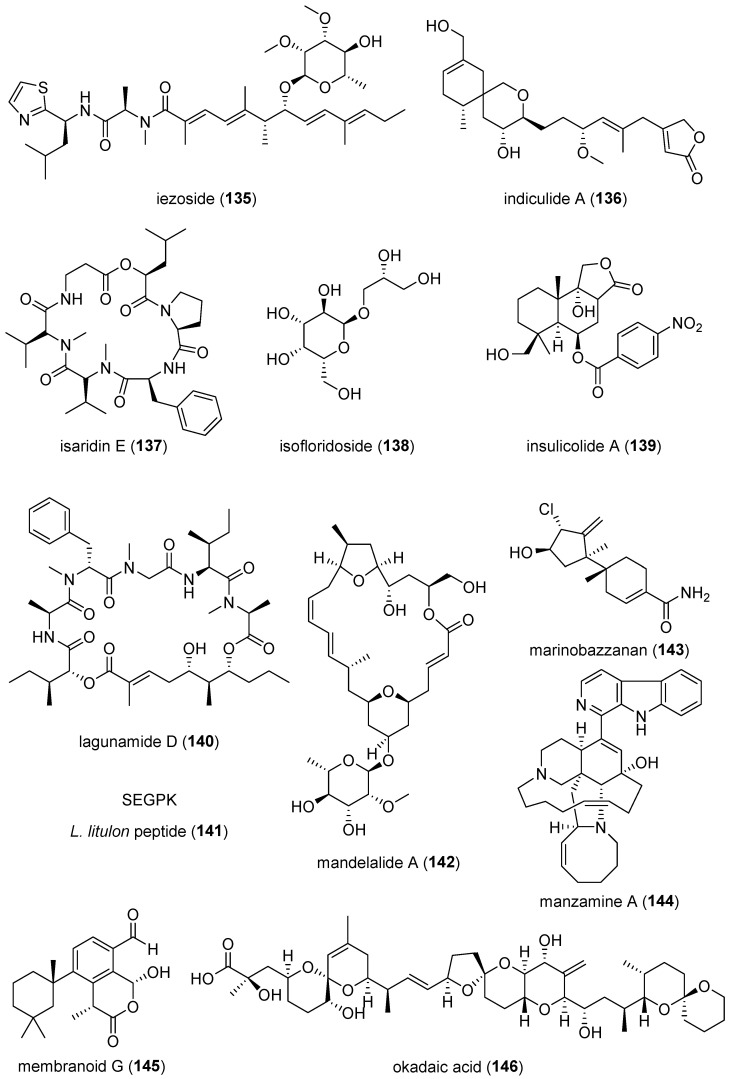

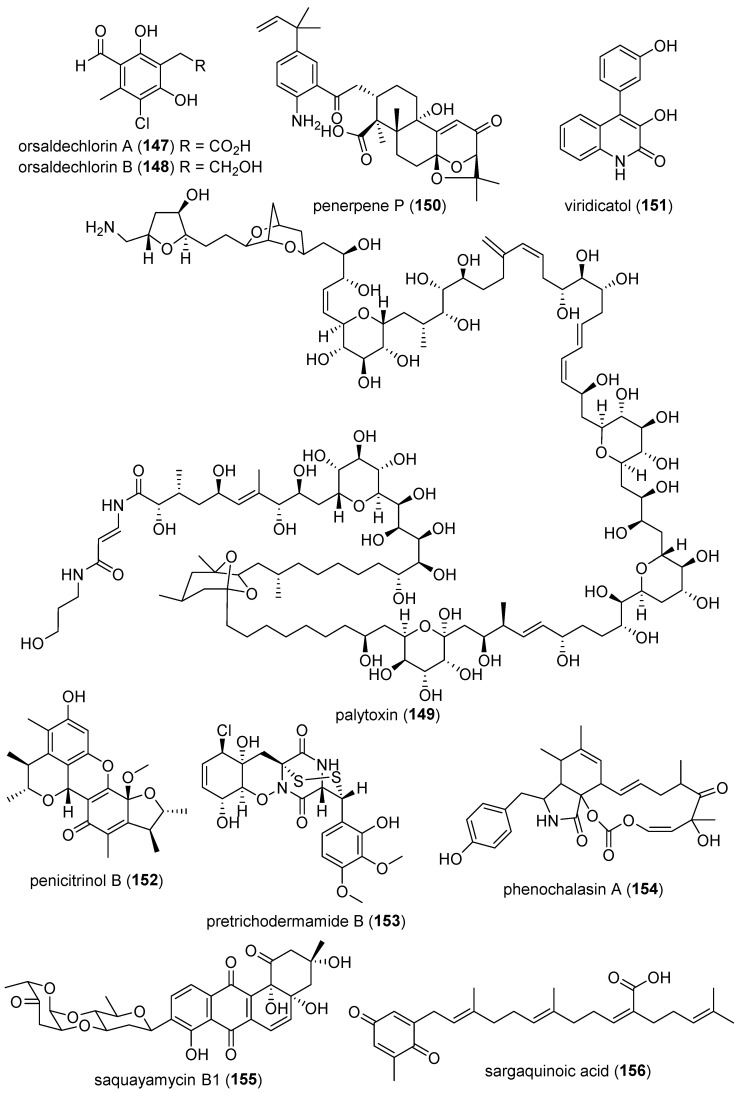

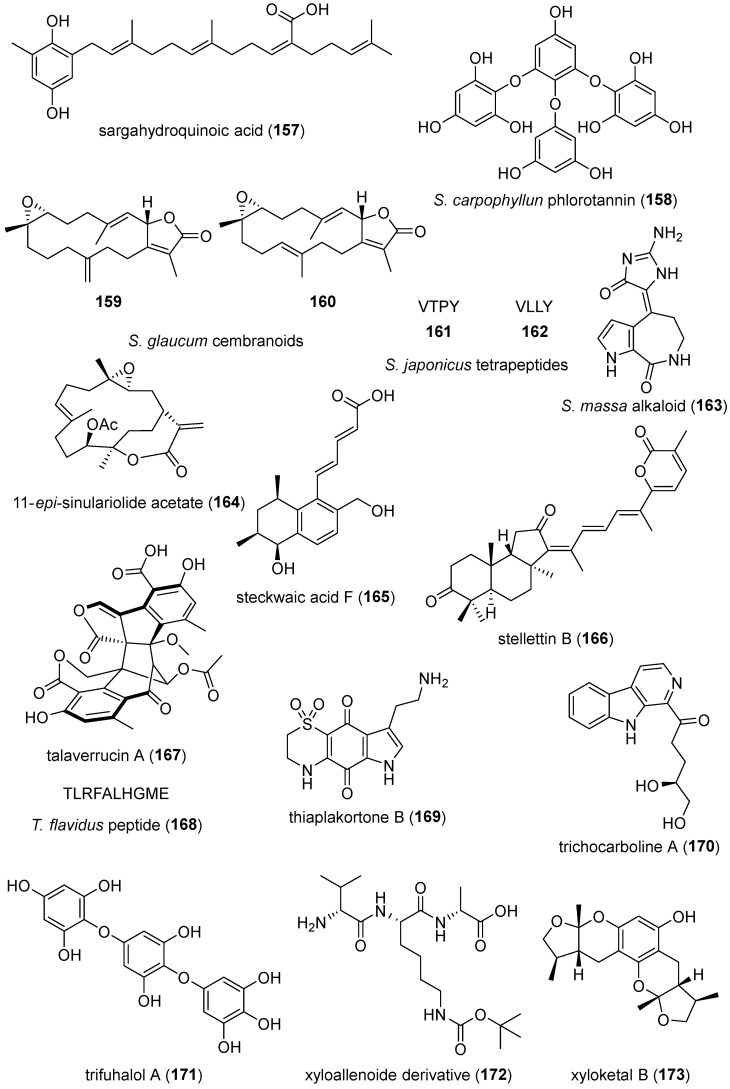
Marine pharmacology in 2022–2023: marine compounds with miscellaneous mechanisms of action.

The present review aims to present the 2022–2023 *preclinical* marine pharmacology literature, with a format similar to our previous 13 reviews of this series, which cover the period 1998–2021 [1,2,3,4,5,6,7,8,9,10,11,12,13]. To search and retrieve the peer-reviewed published literature we used the scientific electronic databases MarinLit, PubMed, PubChem, ScienceDirect, and Google Scholar. Similar to the previous reviews of this series, we have focused on the mechanism of action of structurally characterized marine chemicals, classified into six major chemical classes, namely, polyketides, terpenes, peptides, alkaloids, shikimates, and sugars, including compounds with mixed biogenetic origin, using a modification of Schmitz’s chemical classification [14]. Researchers from 40 countries (Figure 1) have contributed to the collection and identification of compounds from multiple source organisms (Figure 2). Studies on the mechanism of action of marine chemicals demonstrating antibacterial, antifungal, antiprotozoal, antituberculosis, and antiviral pharmacological activities are summarized in Table 1, and the corresponding structures and mechanisms of action are presented in Figure 3 and Figure 4, respectively. Similarly, mechanism-of-action studies with marine compounds that demonstrated activity on the immune and nervous systems activities, as well as antidiabetic, and anti-inflammatory bioactivities, are listed in Table 2, with their respective structures consolidated in Figure 5 and mechanisms of action in Figures 6–8. Marine compounds with miscellaneous mechanisms of action that were reported to affect multiple cellular and molecular targets, but currently are unassigned to a particular pharmacological category, are presented in Table 3, with their structures depicted in Figure 9.

## 2. Marine Compounds with Antibacterial, Antituberculosis, Antifungal, Antiprotozoal, and Antiviral Activities

Table 1 presents 2022–2023 mechanism-of-action studies with 43 structurally characterized marine compounds (**1**–**43**) that demonstrated antibacterial, antituberculosis, antifungal, antiprotozoal, and antiviral pharmacological activities and that are shown in Figure 3 and Figure 4.

### 2.1. Antibacterial and Antituberculosis Activity

As shown in Table 1 and Figure 3, during 2022–2023, studies with 20 structurally characterized marine natural products (**1**–**20**) isolated from bacteria, fungi, worms, sponges, and algae reported antibacterial (including antitubercular) activity via cell membrane damage, activation of autophagy, inhibition of sortase A or elastase, inhibition of the activation of IRF3, inhibition of efflux pumps, downregulation of transcription, inhibition of respiration, and binding to biofilm-forming proteins (Figure 4).

The most common mechanism of action among these studies was damage to the bacterial membrane. Chen and colleagues identified a novel sulfate-modified dibenzopyrone alterlactone 5′-O-sulfate (**2**), from the sponge-derived fungal strain *Alternaria* sp. SCSIOS02F49, with activity against foodborne bacteria. Specifically, they found that their lead compound was able to disrupt the shape and integrity of the *Staphylococcus aureus* cell membrane, as demonstrated by scanning electron microscopy and propidium iodide uptake [16]. Rončević and colleagues found that anisaxin-2S and anisaxin-2P (**3** and **4**, respectively), helical peptides from the parasitic nematode *Anisakis* spp., were bactericidal against *Klebsiella pneumoniae* and *Escherichia coli*, including drug-resistant clinical isolates, with a mechanism dependent on membrane bulging, lipid extraction, and ultimately leakage of bacterial contents from micro-ruptures. Although unstable in vivo, these peptides were notably non-toxic to animal cells, overcoming a frequent obstacle to antimicrobial peptides (AMPs) [17]. Safronova and colleagues analyzed the mechanism of another AMP, capitellacin (**6**), whose disulfide-bonded beta sheet configuration makes it resistant to proteolysis. Specifically, the authors analyzed a panel of capitellacin analogs and fragments, finding that a hydrophilic turn region led to low levels of **6** at inner bacterial membranes and reduced hemolysis of human red blood cells. While inert against human cells, **6** accumulated at the surface of bacterial cells, deforming the membrane bilayer and leading to rupture. Moreover, the compound could eliminate biofilms, particularly those from *E. coli* [19]. Panteleev and colleagues reported a third AMP, namely HfBRI-25 (**10**), with similar structure, safety and stability profile derived from the marine worm *Heteromastus filiformis*. Like **6**, peptide **10** was effective against *E. coli*, including strains producing biofilms, but spared human red blood cells and kidney cells [23]. The mechanism of action of **10** is likely similar to that of **6**, and resistance was not observed even after 21 passages in the presence of **10**. Compound **10** also showed similar IC_50_ values in several drug-resistant strains tested. Juskewitz and colleagues investigated the polyketide lulworthinone (**12**), derived from the marine fungus of the family *Lulworthiaceae*, that showed activity against Gram-positive strains, notably *B. subtilis*, altering membrane permeability and abolishing the potential of the bacterial membrane without affecting its integrity. This activity was associated with mislocalization of the cell division factor FtsZ and with aggregation of **12** [25]. Upender and colleagues assessed (Z)-13-methyltetra-4-decenoic acid ((Z)-4C-14:1, **15**), a free fatty acid isolated from the marine bacterium *Olleya marilimosa*, that inhibited both *B. subtilis* and *E. coli*, while bacterial cytological profiling revealed that *B. subtilis* treated with **15** most closely resembled that treated with colistin, linoleic acid, or daptomycin. Treated cells showed signs of loss of membrane integrity despite intact peptidoglycan layers in Gram-positive bacteria [27]. Guo and colleagues investigated the polyketide trypacidin (**19**), isolated from the marine fungus *Aspergillus fumigatus*, as a treatment for *Vibrio parahaemolyticus*, a serious challenge to the farming of seafood. They determined that this compound exerted its effects via permeabilization of the bacterial membrane and cell wall and leakage of electrolytes, nucleic acids, and proteins (including alkaline phosphatase) from target bacteria. Furthermore, pronounced morphologic changes were observed, with treated bacteria described by the authors as “severely distorted” [31]. Huang and colleagues found that the alkaloid 5′-epiequisetin (**8**), isolated from the marine fungus *Fusarium equiseti* BBG10, inhibited the growth of six strains of *Vibrio* spp.; scanning electron microscopy showed that it resulted from “corrugation” and destruction of the bacterial membrane [21].

Tian and colleagues investigated the alkaloid equisetin (**7**), first isolated from the deep-sea fungus *Fusarium equiseti* NRRL 5337, reporting a mechanism of action distinct from **8**, that cleared intracellular *S. aureus* infections through activation of host cell autophagy and increasing levels of reactive oxygen species (ROS) in host cells via modulation of mitochondria. Encouragingly, they observed antibacterial activity both in vitro and in a mouse model of *S. aureus* infection [20]. Fu and colleagues assessed the polyketide gliotoxin (**20**), isolated from marine deep-sea-derived fungus, *Aspergillus* sp. SCSIO Ind09F01, which was also capable of inducing host cell autophagy to combat infections, demonstrating that **20** activated autophagy in macrophages infected with *Mycobacterium tuberculosis*. Unlike **7**, **20** did not induce ROS in host cells, but nevertheless exerted antitubercular effects via upregulation of autophagic machinery [32].

A particular challenge in antibacterial therapy is the formation of biofilms. Oluwabusola and colleagues studied the peptide bisaprasin (**5**), obtained from the marine sponge *Aplysinella rhax*, which inhibited the quorum sensing system in *Pseudomonas aeruginosa*, specifically by partially inhibiting production of elastase. At higher doses, **5** could suppress the formation of biofilms [18]. Badran and colleagues assessed the polyketide resistoflavin (**18**), a compound from marine *Streptomyces* sp. EG1, that inhibited biofilm formation in both Gram-positive (*S. aureus*) and Gram-negative bacteria (*E. coli*). Molecular docking studies demonstrated a binding interaction between **18** and Clumping Factor B (ClfB) and the lipoprotein CsgG in *S. aureus* and *E. coli*, respectively [30]. Khan and colleagues investigated two fatty acids, myristic (**13**) and oleic acid (**14**), derived from several Irish Sea sponges, and determined their ability to hamper biofilm formation in *S. aureus*. Interestingly, **13** showed greater effects against methicillin-resistant *S. aureus* (MRSA), while **14** was more effective against a methicillin-sensitive strain (MSSA). Mechanistic studies determined that **13** inhibited transcription of the biofilm-related genes fnbA and fnbB in MRSA, and both compounds inhibited icaA in MSSA. Biofilm inhibition was not the result of bactericidal activity in either case, although bacterial growth was delayed [26]. Tran and colleagues investigated the alkaloid lanthelliformisamine C (**11**), found in the marine sponge *Suberea ianthelliformis*, which had bactericidal activity against planktonic *P. aeruginosa*, and could inhibit biofilm formation in select strains. Unlike related compounds in the same study, **11** also inhibited bacterial efflux pumps and was also metabolically stable and non-toxic to human cells at relevant concentrations [24]. These studies together point to inhibition of virulence as a promising strategy to overcome biofilm-related infections.

Additional antibacterial drugs exerted their effects via a broad collection of mechanisms. Zhuravleva and colleagues examined the effects of the polyketide acruciquinone A (**1**), from the marine fungus *Asteromyces cruciatus*, on *S. aureus* with an eye toward treatment of skin infections. They found that **1** inhibited both urease and sortase A activities in bacteria and protected human keratinocytes from damage as evidenced by reduced lactate dehydrogenase release in an infection model [15]. Su and colleagues investigated the terpenoid carotenoid fucoxanthin (**9**), derived from edible brown algae, that was shown to be useful for the treatment of sepsis. While the inhibition of interferon regulatory factor 3 (IRF3) by **9** was already known, they determined that a decrease in pro-inflammatory cytokines in an IRF3-dependent manner led to changes in the makeup of the host microbiome, with increased prevalence of *Akkermansia* spp. and Verrucomicrobiota versus *Morganella* spp. [22]. Kim and colleagues examined the alkaloid prodigiosin (**17**), a red pigment produced by marine bacterium *Hahella chejuensis*, a compound previously seen to have antibacterial, antiprotozoal, and anticancer activities. In *Cutibacterium acnes*, RNA sequencing showed changes in several metabolic pathways, including carbohydrate, energy, and nucleotide metabolism, changes which correlated with lack of growth phase. *C. acnes* also upregulated the stress response protein SigB in response to **17** [29]. Finally, Zhao and colleagues studied the polyketide penicilazaphilone (**16**), derived from the marine fungus strain *Penicillium sclerotiorum* M-22, and leveraged omics approaches to determine the mechanisms underlying the bactericidal action of **16** against *E. coli*. They found that biofilm formation was reduced, the bacterial membrane was destroyed (though to a lesser degree than with ampicillin treatment), and flagella were lost. Proteomic analysis showed these changes resulted from downregulation of respiration, nitrogen and sulfur metabolism, as well as flagellar motion, with increases in reactive oxygen species [28]. Thus **16** encompasses the variety of antibacterial mechanisms presented here.

### 2.2. Antifungal Activity

As shown in Table 1, Figure 3 and Figure 4, during 2022–2023, five studies with structurally characterized marine natural products (**21**–**25**) isolated from bacteria and fungi reported antifungal activity, via cell wall and membrane damage or inhibition of Icl or hyphal adhesion. As with antibacterial agents, the most common mechanism was damage to the cell wall or membrane. Kharat and colleagues analyzed the effects of the shikimate 2,4-di-tert-butylphenol (2,4-DTBP, **24**), derived from marine bacterium *Serratia marcescens* BKACT, on the agricultural threat *Fusarium foetens*. This study determined that 2,4-DTBP treatment led to reduced ergosterol in mycelia and cytoplasmic leakage, though at a higher dose than the reported MIC of 0.03 mg/mL. At 0.53 mM, **24** was also able to prevent spore germination [36]. Jiang and colleagues demonstrated that the peptide isaridin J (**25**), isolated from the marine fungus *Beauveria felina* SYSU-MS7908, worked by disrupting fungal mycelial growth and increasing membrane permeability in the fungus *Geotrichum citri-aurantii*, also known as sour rot, a major postharvest disease in citrus [37]. Zhu and colleagues investigated the polyketide cocultimycin A (**22**), derived from a co-culture of marine fungi *Aspergillus versicolor* IMB17–055 with *A. chevalieri*, and determined its effect on *Candida albicans*. Treatment with **22** led to shrinking and breaking of *C. albicans* cells with loss of hyphae, along with a reduction in genes associated with membrane integrity and chitin synthesis. Collectively, these changes were associated with reduced adhesion and inhibition of biofilm formation [34]. Kamauchi and colleagues assessed the polyketide dehydrocurvularin (**23**), derived from the marine fungus *Curvularia aeria*, and observed it impacted hyphal adhesion of *C. albicans*, yeast-to-hyphal transition and fungal adhesion to human cells, in part via reductions in EFG1, TEC1, ECE1, and HWP1 expression [35]. An additional novel antifungal compound, the alkaloid bacillimide (**21**), was isolated from the marine actinomycete *Streptomyces bacillaris*, and shown to inhibit the transcription, and thus activity, of *C. albicans* isocitrate lyase (ICL), a gene required for the glyoxylate cycle, a metabolic cycle notably absent in human cells. The MIC of **21** was orders of magnitude higher in the presence of glucose, indicating a need for C_2_ carbon utilization for it to be effective [33].

### 2.3. Antiprotozoal Activity

As shown in Table 1, Figure 3 and Figure 4, in 2022–2023, six studies of seven structurally characterized marine natural products (**26**–**32**), isolated from algae and cyanobacteria, reported antiprotozoal effects (antimalarial, antileishmanial, and antitrypanosomal) via induction of cell death, binding to cytochrome C reductase, or inhibition of cruzain. Four of the six studies focused on programmed cell death as a mechanism of drug action. Arberas-Jiménez and colleagues examined the sesquiterpene (+)-elatol (**27**), isolated from the red alga *Laurencia dendroidea*, and its effects on the amoeba *Naegleria fowleri*. An IC_90_ dose of **27** led to condensation of chromatin, plasma membrane permeability (but not destruction), ROS generation, and a significant drop in ATP production, all consistent with apoptotic cell death. Notably, **27** killed both trophozoite and cyst-stage amoeba with similar IC_50_s, while the IC_50_ against murine macrophages was much higher (61.52 vs. 1.08 µM, selectivity index 57) [39]. The authors note that apoptosis would prevent the release of inflammatory parasitic components as would be seen in the case of necrosis, increasing treatment safety. A second study from the same group examined several algal meroterpenoids—6Z-1′-methoxyamentadione (**29**), 1′-methoxyamentadione (**30**), and gongolarone B (**31**), isolated from the brown alga *Gongolaria abies-marina*—for their efficacy against *N. fowleri*, and observed they produced similar effects to **27** on trophozoites, with the addition of loss of mitochondrial potential [42]. San Nicolás-Hernandez and colleagues showed that **29**–**31** were also effective against *Leishmania* spp. and *Trypanosoma cruzi*, the parasites causing leishmaniasis and Chagas disease, respectively, by producing condensed chromatin, membrane permeability, and vacuoles, features of both autophagic and apoptotic cell death [41]. García-Davis and colleagues identified the terpenoid laurequinone (**32**), isolated from the red alga *Laurencia johnstonii*, as an anti-*Leishmania amazoensis* compound. Treatment with **32** resulted in loss of mitochondrial potential, flipping of phosphatidylserine to the outer layer of the membrane, and ROS accumulation, but no loss of membrane integrity or condensation of chromatin [43].

Two additional antiprotozoal agents rely on distinct mechanisms of action, characterized using structural biology and molecular docking approaches. Barbosa Da Silva and colleagues reported that the peptide gallinamide A (**28**), acquired from marine cyanobacterium *Schizothrix* sp., binds to the *Trypanosoma cruzi* cysteine protease cruzain, as do several analogs, and thus might see future development for the treatment of Chagas disease [40]. Finally, Elmaidomy and colleagues investigated the antimalarial properties of a halogenated compound (**26**) obtained from the alga *Halimeda macroloba*, and observed that it bound to and inhibited cytochrome-C reductase, leading to an IC_50_ in the low micromolar range against the protozoan parasite *Plasmodium falciparum*, which causes one of the most severe forms of malaria [38].

### 2.4. Antiviral Activity

As shown in Table 1, Figure 3 and Figure 4, during 2022–2023, 10 studies with structurally characterized marine chemicals (**33**–**43**) isolated from sponges, fungi, algae, bacteria, coral, and ascidians reported antiviral activities. Mechanisms were varied, including inhibition of viral proteases, neuraminidase, PKC, endosome uncoating, replication, and cell egress, as well as host immune response.

Given the focus in recent years on SARS-CoV-2, the virus at the heart of the COVID-19 pandemic, it is hardly surprising that many of the studies identified focused on this virus. ElNaggar and colleagues investigated the polyketide aurasperone A (**34**), isolated from the marine fungus *Aspergillus niger*, and determined the mechanism against SARS-CoV-2. In vitro assays showed efficacy against the virus, preventing virus-induced cytopathic effect (CPE) and yielding a better therapeutic window than remdesivir (selectivity index 2641.5 vs. 41.07) [45]. Moreover, molecular dynamics identified the viral protease M^pro^ (also known as 3CL^pro^) as the most likely target and suggested that binding inhibited the catalytic activity of the enzyme [45]. Alhadrami and colleagues, studying the diketopiperazine alkaloid neoechinulin A (**41**), derived from the Red Sea fungus *Aspergillus fumigatus* MR2012, also found that it potently targeted SARS-CoV-2 M^pro^, as determined by similar molecular dynamics approaches and protein assays [51]. Avalon and colleagues determined that SARS-CoV-2 viral main protease 3CL^pro^ was also the molecular target of the cyclized merosesquiterpenoid tuaimenal A (**43**), derived from the soft coral *Duva florida*. In their study, molecular docking predicted the protease target, which was then confirmed by enzyme assays with recombinant protease. The authors tested additional proteases and showed that the effect was not common across other serine and cysteine proteases [53]. Nagahawatta and colleagues demonstrated that shikimate ishophloroglucin A (**36**), isolated from brown marine algae *Ishige okamurae* and *Ecklonia cava*, inhibited both the 3CL^pro^ and PL^pro^ proteases via competitive inhibition [47]. Molecular docking of **36** with viral proteases was confirmed by in vitro assays and CPE analysis after viral exposure. The same study investigated additional marine compounds that were less potent, but some of which exhibited increased protection from cell death. An additional target in SARS-CoV-2 is the spike protein. Williams and colleagues demonstrated that dimeric biphenyl meroterpenoid thorectidiol A (**42**), isolated from the marine sponge *Dactylospongia elegans*, blocked the interaction between the receptor binding domain (RBD) of the SARS-CoV-2 viral spike protein and ACE2, the receptor required for host cell entry. Notably, the observed IC_50_ of 1 µM, derived from recombinant protein assays, was approximately six-fold lower than that observed for inhibition of the unrelated PD-1/PD-L1 interaction [52]. A final study including SARS-CoV-2 identified a unique mechanism of action and focused still more heavily on another RNA virus: Ebola. Izumida and colleagues examined the alkaloid lamellarin α 20-sulfate (**39**), known to suppress HIV infection. Using pseudotyped HIV with Ebola or SARS-CoV-2 glycoproteins, they showed that **39** could prevent viral infections of mammalian cells, without affecting attachment. In Ebola, the mechanism is believed to inhibit conformational changes of viral proteins in endosomes that facilitate uncoating [49].

Two studies focused on influenza virus. Hu and colleagues examined the alkaloid bacillibactin (**35**), from the mangrove fungus *Arthrinium* sp. SCSIO 41305, which bound and inhibited neuraminidase (NA) at the same site bound by existing influenza drugs zanamivir and oseltamivir, with an IC_50_ approximately ten-fold lower than that of oseltamivir [46]. Cao and colleagues isolated two indole-terpenoids, janthinellumnines A and B (**37**, **38**), from co-cultures of the marine-derived fungi *Penicillium janthinellium* with *Paecilomyces formosus* and investigated their effects on influenza. Both compounds inhibited infection by two flu strains, with IC_50_s ranging from 3.8 to 15.7 µM, and were shown to bind to NA via Arg371, though the IC_50_ for NA exceeded 500 µM, suggesting additional mechanisms of action against influenza still to be determined [48].

Two studies focused on HIV and Herpes simplex virus 2 (HSV-2). Wang and colleagues investigated the sesterterpenoid ansellone J (**33**), derived from the marine sponge *Phorbas* sp., and demonstrated that the compound functions as an HIV latency reversal agent, reactivating transcription of proviral RNA via PKC so that infected cells can be targeted in what is termed a “shock and kill approach to a sterilizing cure for HIV-1 infections” [44]. Sureram and colleagues characterized the peptide A-3302-B (**40**), derived from the marine *Micromonospora* sp., strain MAG 9-7, already known to possess antistaphylococcal and anti-inflammatory properties. In this study, they found that **40** exhibited antiviral activity against HSV-2, including an acyclovir-resistant strain, but not against HSV-1 or cytomegalovirus. Time-of-addition and electron microscopy experiments support a mechanism of action preventing the release of mature virus at the end of the viral replication cycle. Specific molecular targets remain to be elucidated, but the authors note that this mechanism of action makes **40** well-suited for the treatment of established, even drug-resistant infections [50].

## 3. Marine Compounds with Antidiabetic and Anti-Inflammatory Activity, and Affecting the Immune and Nervous Systems

Table 2 presents the 2022–2023 mechanism-of-action studies with structurally characterized marine compounds (**44**–**112**) shown in Figure 5 that demonstrated antidiabetic or anti-inflammatory activity and affected the immune or nervous system.

### 3.1. Antidiabetic Activity

Diabetes is a disease that affects millions of people globally and can cause “… blindness, kidney failure, heart attacks, stroke and lower limb amputation” (https://www.who.int/news-room/fact-sheets/detail/diabetes, accessed on 9 December 2025). As shown in Table 2 and Figure 5, during 2022–2023, 11 structurally characterized marine natural products (**44**–**54**) isolated from algae, bacteria, fungi, sea urchins and sponges reported novel antidiabetic mechanisms of pharmacological action targeting α-amylase and α-glucosidase, peroxisome proliferator-activated receptor ligand γ (PPAR-γ), tyrosine phosphatase 1B, the protein kinase C-iota (PKC-iota) pathway, gluconeogenesis gene expression and advanced glycation end product (AGE) receptor.

Zhang and colleagues contributed a new dibenzo-α-pyrone derivative, alternariol 1′-hydroxy-9-methyl ether (**44**), discovered in the marine-derived fungus *Alternaria alternata* LW37, that demonstrated promising antidiabetic activity resulting from potent in vitro inhibition of α-glucosidase, probably resulting from mixed-type enzyme inhibition [54]. Li and colleagues described a new fungal triterpene asperflagin (**45**), derived from the marine fungus *Aspergillus flavus*, which induced high glucose uptake in vitro without fat accumulation a “PPAR-γ partial agonist” activity [55]. Casertano and colleagues determined that the sesquiterpene quinone avarone (**46**), found in the marine sponge *Dysidea avara*, improved insulin sensitivity by potently and selectively inhibiting both protein tyrosine phosphatase 1B (PTP1B) and aldose reductase [56]. Wang and colleagues demonstrated that bergamotane sesquiterpenoid derivatives brasilterpenes A (47) and C (**48**), isolated from the marine ascomycete fungus *Paraconiothyrium brasiliense* HDN15-135, demonstrated hypoglycemic activity in vivo by suppressing gluconeogenesis gene expression [57]. Li and colleagues evaluated acyclic diterpene cladopsol B (**49**), discovered in the marine jellyfish-derived fungus *Cladosporium oxysporum*, that stimulated glucose update in vitro by moderately activating PPAR-γ as a partial agonist [58]. Chu and colleagues identified polyketide dieckol (**50**), purified from the edible brown alga *Ecklonia cava*, and assessed attenuation of diabetic nephropathy by suppression of the production of AGE by binding of (**50**) to the active site of the AGE receptor and downstream signaling pathways [59]. Pham and colleagues investigated the bioactive dark-red pigment polyketide echinochrome A (**51**), isolated from shells and spines of sea urchins, demonstrating it prevented diabetic murine nephropathy by inhibiting the PKC-iota signaling pathway and enhancing renal mitochondrial function [60]. Li and colleagues reported the antidiabetic potential of a shikimate eurothiocin D (**52**), discovered in the marine fungus *Talaromyces indigoticus* FS688, and determined potent in vitro inhibition of α-glucosidase by binding to a bioactive site “containing the active residues Asp215, Val216, Gly217 or Ser218” [61]. Dat and colleagues showed that polyketide (-)-jasmonic acid (**53**), derived from the mangrove *Rhizophora apiculata* endophytic bacterium *Bacillus* sp. RAR_M1_45, inhibited both α-amylase and α-glucosidase, observations that were “consistent with computational docking results” [62]. Truong and colleagues characterized terpene zonarol (**54**), found in the brown alga *Dictyopteris polypodioides*, as a mixed-type competitive inhibitor of α-glucosidase, as determined by enzyme kinetic studies, with antihyperglycemic and anti-type 2 diabetic activity in vivo [63].

### 3.2. Anti-Inflammatory Activity

As shown in Table 2, Figure 5, and schematically in Figure 6 and Figure 7, in 2022–2023, 35 structurally characterized marine natural products (**9**, **55**–**89**) isolated from fish, shrimp, coral, sea urchins, squid, mollusks, sponges, dinoflagellates, fungi, algae, diatoms, mangrove, bacteria and cyanobacteria were reported to have anti-inflammatory activity both in vitro and in vivo by targeting multiple signal transduction pathways without inducing cytotoxicity.

Several groups identified that marine-derived natural products mechanistically decreased lipopolysaccharide (LPS)-induced nuclear factor kappa-light-chain-enhancer of activated B cells (NF-kB) or other pathways to limit the production of pro-inflammatory cytokines. Vidal and colleagues demonstrated that the nitrogen-containing compound aeroplysinin-1 (**56**), isolated from the sponge *Aplysina aerophoba*, significantly lowered LPS-induced NF-kB and protein kinase B (AKT) activation to decrease interleukin-6 (IL-6) production [64]. Sun and colleagues investigated the terpenoid astaxanthin (**60**) and found that it blocked LPS-induced IL-1β production by decreasing the succinate dehydrogenase (SDH)–hypoxia-inducible factor-1-α (HIF-1α) axis [67]. Furthermore, Jiao and colleagues demonstrated that the secomeroterpenonid dysambiol (**67**), isolated from marine sponge *Dysidea* sp., blocked NF-kB and mitogen-activated protein kinase (MAPK) activation to decrease macrophage tumor necrosis factor-α (TNF-α), IL-1β, and IL-6 production [73]. Cho and colleagues investigated a low-molecular-weight fish collagen peptide (LMWCP) (**72**) characterized from tilapia (*Oreochromis* genus) that decreased both rat osteoarthritis and serum IL-1β, IL-6 and TNF-α levels, by affecting both activation of inflammation and apoptosis pathways [81]. Finally, Yang and colleagues investigated the polyketide somalactam A (**83**), derived from the Arctic sponge-associated actinomycete bacterium *Streptomyces somaliensis* 1107, and observed that it limited LPS-induced macrophage IL-6 and TNF-α production by limiting MAPK phosphorylation [92].

Other groups identified that marine-derived compounds blocked not only the induction of cytokines by LPS, but also the induction of nitric oxide (NO) and superoxide (SOX) production to limit the anti-inflammatory response. Zhang and colleagues reported that the phenylethanol glycoside acteoside (**55**), isolated from the leaves of the mangrove *Acanthus ilicifolius var. xiamenensis*, attenuated dextran sodium sulfate (DSS)-induced mouse chronic ulcerative colitis by blocking Janus kinase (JAK)/signal transducer and activator of transcription (STAT), inducible nitric oxide synthase (iNOS), and NF-kB signaling [63]. Liu and colleagues discovered that the meroterpenoids tricycloalternarene O and P (**57** and **58**), in the marine-derived fungus *Alternaria alternata* JJY-32, blocked LPS induction of NF-kB and subsequent NO and IL-6 production [65]. Cao and colleagues identified ergostane-type sterol aspersterol C (**59**), from the deep-sea-derived fungus *Aspergillus unguis*, as blocking LPS-mediated upregulation of both IL-6 and iNOS [66]. Ren and colleagues showed that polyketide 5-chloro-6-hydroxymellein (**64**), from the mangrove endophytic fungus, *Amorosia* sp. SCSIO41026, inhibited LPS-induced activation of the phosphoinositide 3-kinase (PI3K)/AKT pathway in vivo, resulting in macrophage NO, TNF-α and IL-6 inhibition [70]. Both Al-Awadhi and colleagues, who discovered the monounsaturated fatty acid 7(E)-9-keto-hexadec-7-enoic acid (**65**) from a cyanobacterial mat collected from Delta Shoal, Florida Keys, and Gunasekera and colleagues, who reported the terpenoid dysidazirine carboxylic acid (**66**) from the benthic marine cyanobacterium *Caldora* sp. from Fort Lauderdale, Florida, observed that these compounds blocked LPS induction of iNOS [71,72] and NADPH quinone dehydrogenase 1 expression [71]. Wang and colleagues reported a novel polyketide derivative eschscholin B (**69**), from the mangrove endophytic fungus *Daldinia escholtzii* KBJYZ-1, that decreased LPS-mediated activation of NF-kB and MAPK, resulting in diminished NO production by macrophages [75]. Additionally, Qin and colleagues isolated meroterpenoid littoreanoid K (**71**) from mangrove-derived fungus *Penicillium* sp. HLLG-122, which decreased LPS-induced iNOS and cyclooxygenase-2 (COX-2) expression and IL-6 and TNF-α production by macrophages [80]. Furthermore, Samarakoon and colleagues characterized the terpenoid MCDO (24-methylcholesta-5(6), 22-diene-3β-ol) (**73**), from the cultured marine diatom *Phaeodactylum tricornutum* Bohlin, that limited LPS-mediated NF-kB activation and subsequent macrophage NO, IL-1β and prostaglandin E_2_ (PGE_2_) production [82]. Similarly, Marasinghe and colleagues studied the shikimate phloroglucinol (**77**) purified from brown algae and found that this compound lowered LPS-mediated activation of the 5′ AMP-activated protein kinase (AMPK)/nuclear transcription factor E2-related factor antioxidant response element (Nrf2)/heme oxygenase-1 protein (Ho-1) signaling pathway to limit NO, IL-1β, IL-6, TNF-α, and PGE_2_ production in macrophages [86]. Furthermore, Wang and colleagues isolated the cytosporone derivative polyketide phomotone A (**78**) from the mangrove endophytic fungus *Phomopsis* sp. QYM13 and identified that phomotone A limited LPS-induced iNOS and COX-2 expression and subsequent macrophage NO production [87]. Additionally, Ohno and colleagues isolated prostaglandin A_2_ (**79**) from the soft coral *Lobophytum* sp. and showed it decreased LPS-mediated NF-kB activation in murine macrophage RAW264.7 cells, which resulted in decreased IL-6 and NO production [88]. Ding and colleagues also demonstrated that the cyclopentapeptide pseudoviridinutan F (**80**), resulting from molecular networking-guided isolation from the marine-derived fungus *Aspergillus pseudoviridinutans*, blocked LPS-induced iNOS expression and subsequent macrophage NO production in murine macrophage RAW264.7 cells [89]. Qi and colleagues demonstrated that the novel chromane-type meroterpenoid sargasilol A (**82**), isolated from a China Sea collection of the brown algae *Sargassum siliquastrum*, limited LPS-induced NF-kB signaling and NO, IL-1, IL-6, and TNF-α production in microglia [91]. Additionally, Shin and colleagues reported a new polyketide strepoglyceride F (**85**), from a marine-derived actinomycete *Streptomyces specialis*, that lowered LPS-induced activation of extracellular signal-regulated kinase (ERK)/c-jun N-terminal kinase (JNK)/p38 mitogen-activated protein kinases (p38K) with subsequent inhibition of NO and IL-6 production in murine macrophage RAW264.7 cells [94]. Anh and colleagues demonstrated that the new terpenoid variotin B (**86**), isolated from the deep-sea fungus *Aspergillus unquis* IV17-109, blocked murine macrophage RAW264.7 cells’ IL-6 and iNOS expression [95], and Susan and Salvador Reyes reported that xestoquinone analogs (**87** and **88**) from the marine sponge *Neopetriosia compacta* reduced murine macrophage RAW264.7 LPS-mediated Nrf2 activation, which also limited NO production [96]. Furthermore, Um and colleagues discovered a new cyclic nonapeptide xinghamide A (**89**), isolated from the bacterium *Streptomyces xinhaiensis*, which blocked LPS-induced COX-2 expression, resulting in decreased NO production in murine macrophage RAW 264.7 cells [97].

Numerous reports demonstrate the ability of marine-derived compounds to dampen responses to additional pro-inflammatory stimuli. For example, Nagahawatta and colleagues found that the phlorotannin eckmaxol (**68**) produced by the brown seaweed *Ecklonia maxima* decreased NF-kB and JNK activation induced by particulate matter, resulting in lower lung macrophage NO, PGE_2_, IL-1β, IL-6 and TNF-α production [74]. In another study, Nagahawatta and colleagues studied the sesquiterpene sargachromenol (**81**), which is “highly abundant” in the edible brown alga *Sargassum horneri*, and found that it blocked particulate matter-induced NF-kB and MAPK to limit murine macrophage RAW264.7 cytokine, PGE_2_ and NO production [90]. Denisenko and colleagues communicated that 1-*O*-alkyl-glycerols (**75**), isolated from the squid *Berryteuthis magister*, decreased plasma levels of leukotriene B4 (LTB_4_) and TXB_2_ and gradually improved patient lung function in obese asthmatic individuals after several months of treatment [84]. Lee and colleagues, who isolated the new indanone derivative streptinone (**84**) from the marine sediment-derived bacterium *Streptomyces massiliensis*, demonstrated that this compound inhibited Toll-like receptor-mediated NF-kB activation after exposure to particulate matter, resulting in decreased NO and PGE_2_ production [93]. Furthermore, the impact of ionizing radiation on inflammation induction was limited by marine-derived compounds. For example, Xiao and colleagues reported a new glyceroglycolipid ishigoside (**70**), isolated from the edible brown alga *Ishige okamurae*, that limited HaCaT keratinocyte cell line MAPK, dimeric transcription factor (AP-1) and NF-kB signaling activation after exposure to ionizing radiation, with subsequent decreases in IL-6, IL-8 and matrix metalloproteases [79]. Three studies extended the pharmacology of the terpenoid carotenoid fucoxanthin (**9**): Kang and colleagues reported that fucoxanthin (**9**) found in brown algae mitigated the inhibitory effect of ionizing radiation on the expression of sirtuin 1 (Sirt1) in murine macrophage RAW264.7 cells and the resulting decreases in IL-1β and TNF-α [78]. Ye and colleagues, in a tissue culture model of nonalcoholic fatty liver disease, demonstrated that fucoxanthin (**9**) regulated lipid metabolism, oxidative stress and inflammation via enhanced Nrf2 and AMPK signaling and attenuated liver cell production of IL-1, IL-6 and TNF-α [76]. Ben Ammar and colleagues showed that fucoxanthin (**9**) enhanced PI3K/AKT/Nrf2 signaling in the human liver cancer cell line HepG2 in response to the hepatotoxic contaminant zearalenone, which resulted in the inhibition of hepatic pro-inflammatory cytokine IL-1 β, IL-6 and TNF-α release [77]. Kirindage and colleagues reported the shikimate phlorofucofuroeckol A (**76**), “refined” from the brown algae *Ecklonia cava*, blocked TNF-α/interferon-γ (IFN-γ)-induced NF-kB and MAPK induction, resulting in decreased HaCaT keratinocyte cell line inflammatory cytokine and chemokine production [85]. Brancaccio and colleagues investigated the alkaloid ovothiol (**74**) that was purified from sea urchin *Paracentrotus lividus* eggs, which also limited TNF-α-induced ERK and JNK phosphorylation in ex vivo skin tissue biopsies to reduce IL-6, IL-8 and TNF-α production [83]. Furthermore, Liu and colleagues observed that the alkaloid aszonalenin (**61**) from the marine coral endophytic fungus *Aspergillus terreus* blocked ox-LDL-induced inflammation by inhibiting the MAPK and PI3K/AKT phosphorylation pathways to lower HUVEC NO, TNF-α and IL-6 production [68]. McCall and colleagues reported that brevetoxin analogs (**62** and **63**) derived from the dinoflagellate *Karenia brevis* promoted the expression of both M1 and M2 markers in human monocyte cell line THP-1, but they were cytotoxic to CD14+ monocytes [69].

### 3.3. Marine Compounds with Activity on the Immune System

As shown in Table 2 and Figure 5, during 2022–2023, four structurally characterized marine natural products (**51**, **90**–**93**) isolated from algae, clams, sea urchins, and sponges were reported to have activity on the immune system in vitro and in vivo by targeting signal transduction pathways.

Kudryashova and colleagues identified an “unprecedented marine glycolipid” assimiloside A (**90**) from the Northwestern Pacific marine sponge *Hymeniacidon assimilis* and found that this compound minimized murine macrophage RAW264.7 LPS-induced reactive oxygen species (ROS) increases by stimulation of lysosomal activity [98]. Several reports analyzed the immunomodulatory properties of marine-derived compounds in vivo. For in vivo studies, Zhao and colleagues reported a pentadecapeptide (**91**) isolated from the clam *Cyclina sinensis* that induced IL-1β, IL-6 and TNF-α in mice that were immunosuppressed with cyclophosphamide and could also activate the MAPK/NFκB and PI3K/Akt pathways [99]. Liao and colleagues studied the protective effects of the phlorotannin eckol (**93**) purified from the brown algae *Ecklonia cava*, and observed that it downregulated TLR4/NF-kB/STAT3 pathways to dampen dextran sulfate sodium-induced mouse chronic ulcerative colitis [102].

Additional in vivo studies addressed the impact of marine compounds on immune function, especially in allergy models. Kim and colleagues investigated the anti-allergic effect of 3,4-dihyroxybenzaldehyde (DHB) (**92**), isolated from the marine red alga *Polysiphonia morrowii*, in mouse bone marrow-derived mast cells and observed that DHB decreased levels of allergy-promoting cytokines IL-4, IL-5, IL-6, and IL-13 through inhibition of NF-kB signaling [100]. Additionally, Abdelmawgood and colleagues reported that the dark-red polyketide echinochrome A (**51**), isolated from the Mediterranean sea urchin *Paracentrotus lividus*, decreased serum IgE, IL-4 and IL-1β in an in vivo model of allergy by modulating the Kelch-like ECH-associated protein 1 (Keap1)/Nrf2 signaling pathway [101].

### 3.4. Marine Compounds Affecting the Nervous System

As shown in Table 2, Figure 5 and Figure 8, in 2022–2023, studies with 24 structurally characterized marine natural compounds (**9**, **51**, **60**, **62**, **76**, **81**, **94**–**112**) isolated from algae, cyanobacteria, fungi, sponges, sea anemones, sea cucumbers, soft corals, cone snails and sea urchins reported novel pharmacological activities for modulating neurodegenerative disorders such as Alzheimer’s disease (AD) and Parkinson’s disease (PD), neuroprotective effects related to ischemia and reperfusion, effects on pain perception, anti-epileptic potential, and modulation of ion channels.

Fifteen compounds (**9**, **60**, **76**, **81**, **94**, **96**, **98**–**100**, **103**, **105**–**107**, **109**, **111**) were shown to demonstrate promising effects for various neurodegenerative disorders, of which nine compounds (**9**, **60**, **76**, **94**, **96**, **99**, **100**, **103**, **107**) might inform the development of novel Alzheimer’s disease (AD) therapeutics. Miao and colleagues reported that the alkaloid aaptamine (**94**), isolated from the South China Sea marine sponge *Aaptos suberitoides*, inhibited butyrylcholinesterase (BChE) and acetylcholinesterase (AChE) by binding directly to the peripheral anionic site (PAS) and the catalytic active site (CAS). In AD, acetylcholine (ACh) deficiency has been shown to contribute to cognitive and behavioral symptoms, and four FDA-approved drugs for AD are AChE inhibitors, with rivastigmine inhibiting both AChE and BChE. In advanced AD, BChE is increased and has been shown to metabolize ACh. In vivo studies in an aluminum chloride-induced zebrafish model of AD demonstrated that aaptamine improved dyskinesia recovery rates similar to donepezil, an FDA-approved AChE inhibitor [104]. Park and colleagues evaluated the shikimate phlorofucofuroeckol A (**76**), a phlorotannin isolated from the marine alga *Ecklonia cava*, which exhibited mixed inhibition of both AChE and BChE using the spectrophotometric method. Molecular docking analysis revealed binding to the acyl loop region of AChE and BChE [130]. Jiang and colleagues showed that the terpenoids amphichoterpenoids D and E (**96**), isolated from the marine fungus *Amphichorda felina*, demonstrated AChE inhibition via a modified Ellman method, and molecular docking showed binding of the PAS [107], which enhances catalytic efficiency by facilitating ACh movement towards the catalytic site. Wang and colleagues also used Ellman’s method and molecular docking studies in vitro and determined that all-trans isomers of astaxanthin (**60**), an antioxidant produced in a variety of marine organisms, competitively and reversibly inhibited AChE by binding to the CAS and PAS. In AD, Ab peptides aggregate into plaques and are indicated in disease progression. In PC12 cells treated with amyloid beta (Aβ)_25–35_, treatment with all-trans astaxanthin resulted in increased cell viability, increased catalase (CAT) and superoxide dismutase (SOD) activity, and decreased AChE in a dose-dependent manner. Both SOD and CAT reduce oxidative stress, with SOD converting reactive oxygen species (ROS) into less reactive molecules and CAT converting H_2_O_2_ to molecular oxygen and water [111]. Paramakrishnan and colleagues investigated the effects of astaxanthin (**60**) in a zebrafish model of diabetic cerebrovascular disease (CVD)-associated AD. They revealed that astaxanthin treatment improved behavioral responses in light and dark chamber, color recognition, and T-maze tests, and attenuated changes in fasting blood glucose levels, tissue extravasation indicated via Evans blue dye, and brain tissue biomarker changes in nitrite, Aβ peptides, matrix metalloproteinase-13 (MMP-13), and AChE. MMP-13 alters the blood–brain barrier (BBB) and contributes to CVD progression and neurodegeneration [112]. Amyloid precursor protein (APP) is cleaved by b-site APP cleaving enzyme 1 (BACE1) to generate Aβ. Fujihara and colleagues evaluated the polyketide aurasperone F (**99**), collected off the coast of Japan from the marine fungus *Aspergillus* sp., with thioflavin-T and fluorescence resonance energy transfer (FRET) assays showing aurasperone F inhibited Aβ aggregation and BACE1 activity. In SH-SY5Y cells, a WST-8 assay showed neuroprotective effects against Aβ toxicity, suggesting therapeutic potential [113]. Nie and colleagues studied butyrolactone (**100**), isolated from the marine fungus *Aspergillus terreus* C23-3, in a zebrafish aluminum trichloride (AlCl_3_)-induced cognitive deficit model. Zebrafish pretreated with butyrolactone for 20 days prior to AlCl_3_ treatment resulted in amelioration of cognitive deficits, as evaluated in the T-maze test, concomitant with a reduction in pro-inflammatory cytokines IL-1β and TNF-α, a reduction in AChE, and a dose-dependent increase in antioxidant reduced glutathione (GSH) levels [114]. Zhang and colleagues evaluated the sesquiterpene copteremophilane G (**103**), isolated from the marine fungus *Penicillium copticola*, and showed that pretreatment increased cell viability in Aβ_25-35_-treated PC12 cells, and dose-dependently reduced lactate dehydrogenase (LDH) and malondialdehyde (MDA), which are markers for cell injury and lipid peroxidation [118]. Zhao and colleagues studied a peptide FYDWPK (**107**), derived from the sea cucumber *Stichopus japonias*, in a scopolamine-induced mouse model of dementia. The investigators observed that pretreatment with the FYDWPK peptide showed no toxicity and improved outcomes in several models used to assess murine learning and memory, by improving “oxidative imbalance, reliev(ing) choline dysfunction, and reduc(ing) hippocampal nerve cell apoptosis.” [123]. Hong and colleagues evaluated the terpenoid fucoxanthin (**9**), extracted from the seaweed *Sargassum oligocystrum*, and showed it inhibited AChE, protected cell viability in C6 cells pretreated with H_2_O_2_ or Aβ_25-35_, and increased the activity of antioxidants CAT and glutathione peroxidase (GPx), glycogen synthase kinase 3b (GSK-3b) expression, and choline acetyltransferase (CHAT) and vesicle acetylcholine transporter (VAChT), which are involved in the biosynthesis of ACh [125].

Seven compounds (**9**, **81**, **98**, **105**, **106**, **109**, **111**) displayed therapeutic potential for Parkinson’s disease (PD). Xiao and colleagues evaluated the alkaloid asperpendoline (**98**), discovered from the co-cultivated fungi of *Aspergillus ochraceus* MCCC 3A00521 and *Penicillium* sp. HUBU 0120, and showed it protected neuroblastoma cell line SH-SY5Y from H_2_O_2_-induced injury via inhibition of Keap1 expression, enabling Nrf2 translocation to the nucleus and an upregulation of detoxifying genes Ho-1 and NQO1 and resulting in the reduction of ROS, which are implicated in PD etiology [109]. Sanguanphun and colleagues studied the medium-chain fatty acid decanoic acid (**105**), isolated from the Black Sea cucumber *Holothuria leucospilota*, and showed that in a 6-hydroxydopamine (6-OHDA)-induced neurotoxic nematode *Caenorhabditis elegans* (*C. elegans*) model, decanoic acid improved behavioral function, reduced dopaminergic neurodegeneration, improved dopaminergic-dependent behaviors, and reduced oxidative stress, while increasing expression of the antioxidant SOD-3 and heat shock proteins (HSP) involved in the stress response, and thus decreased ROS involved in the progression of PD [120]. Sanguanphun and colleagues also evaluated palmitic acid (**111**), isolated from the sea cucumber *Holothuria leucospilota*, in the 6-OHDA-treated nematode *C. elegans* model, observing that treatment with palmitic acid prolonged lifespans, improved behavioral function, decreased ROS, upregulated the antioxidant genes *gst-4*, *gst-10*, and *gcs-1*, and reduced α-synuclein aggregation, findings that contributed to explaining the molecular mechanism of palmitic acid-mediated protection against PD-like pathologies [129]. Wang and colleagues evaluated the alkaloid dehydro-shearinine A (**106**), isolated from mangrove sediment-derived fungus *Penicillium* sp. UJNMF0740, in 6-OHDA-induced rat pheochromocytoma PC12 cells, and showed that it was neuroprotective as it decreased apoptosis, reduced both pro-apoptotic genes and ROS, and increased phosphorylation of the PI3K/Akt signaling pathway [121]. Han and colleagues investigated the terpenoid sargachromenol (**81**), isolated from the marine brown seaweed *Sargassum horneri*, and showed pretreatment with sargachromenol prevented glutamate-induced apoptosis in the HT-22 mouse hippocampal neuronal cell line, increased anti-apoptotic expression of BCL-2, decreased ROS production, enhanced antioxidant proteins Nrf-2 and HO-1, and inhibited phosphorylation of ERK, JNK, p38 and NF-kb, indicating therapeutic potential in neurodegenerative diseases [131]. Liu and colleagues found that in 6-ODHA-treated rat pheochromocytoma PC12 cells exposed to 200 mM levodopa (L-DOPA), pretreatment with the marine carotenoid fucoxanthin (**9**), extracted from brown seaweed, showed decreased mitochondrial damage, decreased ROS, and downregulated proteins involved in apoptosis including ERK1/2, JNK1/2, c-Jun, and cleaved caspase-3. Furthermore, in a mouse model of PD treated with L-DOPA, pretreatment with fucoxanthin improved performance on pole climbing, swimming, and suspension tests, had protective effects on dopaminergic neurons in the substantia nigra, and also reduced expression of ERK1/2, JNK1/2, and c-Jun in midbrain tissue [124]. Kvetkina and colleagues studied a Kunitz-type peptide HMIQ3c1 (**109**), derived from the sea anemone *Heteractis magnifica*, and showed that pretreatment with the peptide inhibited intracellular ROS and NO formation in Neuro-2a cells treated with paraquat and rotenone, and binding to purinergic P2X7 channels (P2X7R) affected stable calcium response in murine neuroblastoma cell line Neuro2a [127].

Two compounds (**60**, **104**) showed neuroprotective effects related to cerebral ischemia and reperfusion injury (CIRI). Park and colleagues described that pretreatment with the terpenoid astaxanthin (**60**) increased the expression of SOD enzymes (SOD1 and SOD2) and attenuated the loss of pyramidal cells in the hippocampus of gerbils with severe ischemia and reperfusion (IR) injury [110]. Qi and colleagues studied the East China Sea diterpene 18-acetoxy-6,7-epoxy-4-hydroxydictyo-19-al (**104**), isolated from the brown alga *Dicyota coriacea*, and reported that protective effects against H_2_O_2_-induced oxidative damage in PC12 cells included dose-dependent inhibition of malondialdehyde (MDA) production and increased glutathione, GPx, and SOD. Furthermore (**104**) upregulated the Nrf2-antioxidant response element (Nrf2-ARE) signaling pathway, leading to significant neuroprotection in a cerebral ischemia–reperfusion injury (CIRI) in vivo [119].

Three compounds (**94**, **108**, **110**) demonstrated effects on pain perception. Sung and colleagues reported that the alkaloid aaptamine (**94**), isolated from the marine sponge *Aaptos* sp., demonstrated analgesic effects in a rat chronic constriction injury (CCI)-induced peripheral neuropathic rat model. Aaptamine decreased CCI-induced neuronal lactate dehydrogenase A (LDHA) and nuclear translocation and attenuated vascular endothelial growth factor (VEGF) upregulation in astrocytes, suggesting the antinociceptive effects of aaptamine were mediated through LDHA and VEGF inhibition [105]. Gladkikh and colleagues showed that the native peptide toxin Hmg 1-b2 (HCR 1-b2, **108**), purified from the sea anemone *Heteractis magnifica*, inhibited the rat acid-sensing ion channel (ASIC) ASIC3 transient current. The analgesic activity of the peptide in a murine acid-induced muscle pain model was similar and comparable to diclofenac, a non-steroidal anti-inflammatory agent [126]. Logashina and colleagues investigated the peptide Ms 9a-1 (**110**), isolated from the sea anemone *Metridium senile*, and demonstrated in frog oocytes using a patch-clamp technique that it potentiates the transient receptor potential ankyrin 1 (TRPA1) channel, which is involved in pain sensation. In an allyl isothiocyanate-induced mouse model of pain, treatment with Ms 9a-1 decreased paw edema, number of paw licks, and duration of paw guarding, while in a CFA-induced inflammation model treatment with Ms 9a-1 reduced paw edema and thermal hyperalgesia [128].

One compound, territrem F (**112**), a meroterpenoid isolated from the South China Sea soft coral *Lobophytum crassum*-derived fungus *Alternaria* sp. ZH-15, was shown to have anti-epileptic potential. Wang and colleagues evaluated territrem F (**112)**, and demonstrated that it inhibited both spontaneous calcium oscillations and 4-amino-pyridine (4-AP)-induced epileptic discharges in murine primary neocortical cultures [132].

Six compounds (**51**, **62**, **95**, **97**, **101**, **102**) were shown to affect ion channels. An and colleagues demonstrated that a Kunitz peptide AsKC11 (**95**), isolated from the sea anemone *Anemonia sulcata* venom, was the first peptide to directly activate neuronal G protein-coupled inward-rectifier potassium (GIRK) channels 1/2 in a reversible manner, “resulting in larger K+ currents…” suggesting pharmacological potential in various neurological and psychiatric conditions [106]. In one study, Mehrotra and colleagues showed that in murine primary neocortical cultures, the voltage-gated sodium channel (VGSC) activator peptide antillatoxin (**97**), isolated from the cyanobacterium *Moorea producens*, stimulated release of glutamate and enhanced neurite outgrowth through an N-methyl-D-aspartate (NMDA) receptor, brain-derived neurotrophic factor (BDNF), and the tropomyosin-related kinase B (TrkB) signaling pathway and may represent a novel strategy to promote neurite outgrowth and neuronal plasticity [108]. In an additional investigation, Mehrotra and colleagues also evaluated the polyketide and voltage-gated sodium channel activator brevetoxin 2 (PbTX-2) (**62**), isolated from the dinoflagellate *Karenia brevis*, in murine primary cerebrocortical cultures, where they reported neuronal morphology changes resulting in increased neurite outgrowth that were blocked by an NMDA receptor 2b (GluN2B)-specific inhibitor ifendopril, a Rac1-selective inhibitor NSC23766, and a p21-activated kinase (PAK)-1 inhibitor IPA-3, demonstrating that PbTX-2-induced neurite outgrowth occurred via the NMDA-PAK1 pathway [115]. Wang and colleagues studied a novel a-4/7 conotoxin peptide QuIA (**101**), from the marine cone snail *Conus quercinus*, in frog oocytes expressing various nicotine receptors from mouse, rat, or human. Using a two-electrode voltage clamp technique, they showed that QuIA selectively and efficiently blocked rat α3β2 and α6/α3β4 nicotinic Ch receptor (nAChR) subtypes, which are expressed in the dorsal root ganglion and contribute to pain pathways [116]. Wang and colleagues showed the novel αM-superfamily conotoxin peptide SIIID (**102**), from the fish-hunting cone snail *Conus striatus*, reversibly inhibited human a7 nAChR in patch clamp experiments with human kidney HEK293 cells expressing a7 nAChR. The a7 nAChR has been indicated in pain, inflammatory diseases, and various cognitive symptoms associated with human neurological disorders [117]. Kim and colleagues studied the naphthoquinoid pigment polyketide echinochrome A (**51**), isolated from sea urchins, using patch clamp assays on human kidney HEK293 cells expressing selected ion channels. They showed that echinochrome A inhibited the calcium channel transient receptor potential vanilloid 3 (TRVP3) and Ora1 and, when combined with the agonists arachidonic acid (AA) or 2-aminoethoxydiphenyl borate (2-APB), facilitated the two-pore domain potassium channels TREK-1 (KCNK2), TREK-2 (KCNK10), and TRAAK (KCNK4), indicating anti-inflammatory and analgesic properties [122].

## 4. Marine Compounds with Miscellaneous Mechanisms of Action

Table 3 presents the 2022–2023 mechanism-of-action studies with structurally characterized marine compounds (**9**, **36**, **50**, **51**, **60**, **113**–**173**) shown in Figure 9 that demonstrated miscellaneous mechanisms of action.

As reported in the 2022–2023 peer-reviewed literature, Table 3 presents 65 marine compounds (**9**, **36**, **50**, **51**, **60**, **113**–**173**) shown to affect multiple cellular and molecular targets with miscellaneous mechanisms of action, but with no assignment to a particular pharmacological category. These compounds have been isolated and characterized from marine ascidians, fish, sea urchins, oysters, sea cucumbers, sponges, soft corals, hydroids, octopus, fungi, algae, bacteria and cyanobacteria, with their corresponding structures shown in Figure 9: marine South Sea sponge *Aaptos suberitoides* aaptamine derivative (**113**) inhibited cyclin-dependent kinase-2 and induced G1 cell cycle arrest [133]; marine red alga *Gracilaria coronopifolia*-derived anhydrodebromoaplysiatoxin (**114**) activated mammalian target of rapamycin (mTOR)/p70 ribosomal protein S6 kinase (p70S6K)/Forkhead box O3(FoxO3a) signaling and autophagy induction inhibition [134]; marine sponge *Agelas nakamurai*-derived alkaloid ageladine A, a pyrrole-pyridinimidazole alkaloid (**115**), suppressed angiogenesis, angiogenesis-related proteins and sirtuin 6 [135]; marine sponge *Agelas nakamurai*-derived sesquiterpene guanidine (−)-agelasidine A (**116**) induced caspase-mediated extrinsic and intrinsic apoptosis pathways [136]; marine microalga *Aurantiochytrium mangrovei* BT3-derived (24*R*)-4α-methyl-5α-stigmata-7,22-dien-3β-ol (**117**) evidenced hypolipidemic effects by stimulation of peroxisome proliferator-activated receptor-α (PPAR-α) transcriptional activity [137]; marine-fish derived histidine dipeptide anserine (**118**) protected against liver injury in vitro by regulation of the Kelch-like ECH-associated protein 1 (Keap1)–nuclear transcription factor E2 (Nrf2) and c-jun N-terminal kinase (JNK)–caspase-3 signaling pathways [138]; marine shrimp-derived xanthophyl astaxanthin (**60**) suppressed blue light-induced phototoxicity in retinal epithelium cells by ^1^O_2_ scavenging [139]; marine oyster *Crassostrea hongkongensis*-derived peptide WNLNP (**119**) demonstrated anti-skin-photoaging effects on ultraviolet (UV)B-irradiated cells in vitro through ROS and matrix metalloproteinases (MMP)-1 inhibition and mitogen-activated protein kinase (MAPK)/nuclear factor kappa-light-chain-enhancer of activated B cells (NF-κB) signaling inhibition [140]; marine sea cucumber *C. frondosa*-derived phospholipids (**120**,**121**) ameliorated hepatic steatosis and activated hypothalamic autophagy in high-fat-diet-fed mice by regulating PPAR-γ and CD36 [141]; marine sponge *Theonella affinis swinhoei*-derived peptide cyclotheonellazole A (**122**) alleviated acute lung injury in vivo by potent inhibition of elastase as well as inflammatory cell infiltration [142]; marine fungus *Chromolaenicola* sp. (HL-114-33-R04)-derived anthraquinone danthron (**123**) was shown to be a potent angiogenesis inhibitor that significantly inhibited MMP-2 secretion [143]; marine hydroid *Dentitheca habereri*-derived diacylated zoanthoxanthin alkaloid dentithecamide A and B (**124**,**125**) inhibited fusion gene of PAX3 (a developmental transcription factor) and FOXO1 (a regulator of cell cycle and apoptosis (PAX3-FOXO1) transcriptional activity while upregulating apoptosis and cytokine signaling genes [144]; marine bacterium *Streptomyces* sp. CNQ-617-derived tricyclic quinazoline alkaloid deoxyvasicinone (**126**) affected human and murine melanin synthesis in vitro by inhibiting tyrosinase and tyrosinase-related protein 1 and 2 expression [145]; marine ascidian-derived cyclic peptide didemnin B (**127**) inhibited translation by preventing aminoacyl-tRNA accommodation into the ribosomal A site by binding to GTPase elongation factor-1 alpha [146]; marine mangrove endophytic fungus *Diaporthe* sp. SYSU-HQ3-derived isoprenylisoindole alkaloid diaporisoindole B (**128**) reduced lipid accumulation in macrophage-derived foam cells by activating PPAR-γ and inhibiting mitogen-activated protein kinase (MAPK) phosphorylation [147]; marine brown alga *Eisenia bicyclis*-derived dieckol (**50**) ameliorated murine ultraviolet B-induced skin wrinkling and skin hydration by reducing collagen degradation and expression of MMP-1, -3 and -9 [148]; marine fungus *Acremonium citrinum*-derived dihydrotrichodimerol (**129**) prevented hepatic fatty liver disease in vivo by PPAR-γ activation and upregulation of sirtuin-1 [149]; marine sea urchin-derived naphthoquinone polyketide pigment echinochrome A (**51**) promoted maintenance and antioxidative regeneration of murine and human intestinal epithelium by increasing expression of LGR5 and MUC2 [150] and melanin synthesis inhibition in a murine melanoma cell line by downregulation of the cAMP response element-binding protein (CREB) signaling pathway [151]; marine fungus *Stachybotrys longispora*-derived rare pyran-isoindolone alkaloid FCGFC1 (**130**) was shown to inhibit proliferation in epidermal growth factor receptor-mutant non-small-cell lung cancer cells by triggering G0/G1 cell cycle arrest and apoptosis [152] and promoted fibrin lysis in vitro with “significant differences in the density, diameter, and distance of the fibrin pores” [153]; edible marine brown alga *Sargassum horneri*-derived fucosterol (**131**) protected tumor necrosis factor-α (TNF-α)/IFN-γ-stimulated human dermal fibroblasts against oxidative stress and inflammation by upregulating the Nrf2/heme oxygenase-1 (HO-1) and NF-κB/MAPK signaling pathways [154]; marine brown alga-derived xanthophyll carotenoid fucoxanthin (**9**) reduced murine atherosclerosis and associated pyroptosis by modulation of phosphoinositide 3-kinase (PI3K)/protein kinase B (Akt) and TLR4/NF-κB signaling activation [155] and arrested cell cycle at G0/G1 phase in small lung cancer cells by affecting the PI3K/Akt signaling pathway [156]; marine Beibu Gulf coral-derived fungus *Aspergillus unguis* GXIMD 02505 polyketide guisinol (**132**) inhibited osteoclastogenesis in vitro by blocking NF-κB p65 nuclear translocation [157]; marine sponge *Axinella carteri*-derived 10Z-hymenialdisine (**133**) suppressed angiogenesis in pancreatic cancer in vitro by inhibition of NF-κB activity [158]; marine sea cucumber *Holothuria scabra*-derived triterpenoid saponin holothurin A (**134**) modulated epithelial–mesenchymal transition in prostate cancer metastasis by inhibition of the Akt/JNK and P38MAPK signaling pathways [159]; marine cyanobacterium *Leptochromothrix valpauliae*-derived peptide–polyketide hybrid glycoside iezoside (**135**) was shown to increase in cytosolic Ca^2+^ oscillations in cancer cell lines by affecting sarcoplasmic/endoplasmic reticulum Ca^2+^-ATPase [160], and causing G_1_/S cell-cycle delay and apoptosis-signaling pathway activation [169]; marine Old Woman octopus *Cistopus indicus*-derived indiculide A (**136**) demonstrated strong inhibition of 5-lipoxygenase inhibition in vitro by a non-competitive mechanism [161]; marine fungus *Amphichorda felina* SYSU-MS7908-derived cyclodepsipeptide isaridin E (**137**) inhibited ADP-induced platelet aggregation in vitro by significant inhibition of PI3K and Akt phosphorylation and signaling pathways [162]; marine red alga-derived galactoclycerol isofloridoside (**138**) activated the sweet taste receptor T1R2/T1R3 by stimulating an increase in intracellular Ca^2+^ and extracellular signal-regulated kinase (ERK) phosphorylation [163]; marine edible brown alga *Ishige okamurae*-derived phlorotannin isophloroglucin A (**36**) inhibited osteoclast differentiation in vitro by affecting expression of nuclear factor of activated T cells 1 (NFATc1) and c-Fos expression [164], and inhibited adipogenesis in vitro and as well as protein tyrosine phosphatase 1B with high and stable binding affinity to the active site [165]; marine fungus *Aspergillus ochraceus*-derived nitrobenzoyl sesquiterpenoid insulicolide A (**139**) prevented osteoclastogenesis in vitro by inhibiting the c-Fos- and NFATc1 signaling pathway [166]; marine cyanobacteria *Dichothrix* sp. and *Lyngbya* sp.-derived cyclodepsipeptide lagunamide D (**140**) induced mitochondrial dysfunction, probably by affecting the voltage-dependent anion-selective channel protein 3 gene [167]; marine monkfish *Lophius litulon* swim bladder-derived peptide (**141**) inhibited angiotensin-1-converting enzyme and demonstrated hypotensive activity by modulating levels of nitric oxide and endothelin-1 in human umbilical vein endothelial cells [168]; marine cyanobacterium *Leptochromothrix valpauliae*-derived peptide–polyketide iezolide (**135**) potently inhibited sarcoplasmic/endoplasmic reticulum Ca^2+^-ATPase [169]; marine ascidian *Lissoclinum* sp.-derived mandelalide A (**142**) was reported to be an indirect activator of AMP-activated protein kinase (AMPK) dependent on the presence of the AMPKα subunit [170]; marine fungus *Acremonium* sp. strain CNQ-049-derived bazzanane-type sesquiterpenoid marinobazzanan (**143**) inhibited cancer-cell migration and invasion by KITENIN protein expression downregulation [171]; marine sponge-derived β-carboline alkaloid manzamine A (**144**) attenuated autophagosome degradation in breast cancer cells by receptor interacting protein-1 over-expression, resulting from AKT/mTOR pathway activation [172]; marine Antarctic sponge *Dendrilla antarctica*-derived diterpene membranoid G (**145**) strongly inhibited supercoiled DNA relaxation by irreversibly binding to DNA topoisomerase 1B [173]; marine dinoflagellate-derived lipophilic okadaic acid (**146**) downregulated cytochrome P450 enzymes through NF-κB and Janus kinase (JAK)/signal transducer and activator of transcription (STAT) signaling pathway activation in human hepatocarcinoma cells [174]; marine fungus *Acremonium sclerotigenum* GXIMD 02501-derived orsadelchlorins A and B (**147**,**148**) suppressed osteoclast differentiation by inhibiting NF-kB signaling activation [175]; marine soft coral *Palythoa* sp.-derived polyether palytoxin (**149**) induced apoptotic cell death by downregulation of anti-apoptotic myeloid cell leukemia-1 and B-cell lymphoma extra-large proteins [176]; marine soft coral-derived fungus *Aspergillus* sp. ZF-104 indole-diterpenoid penerpene P (**150**) inhibited protein tyrosine phosphatase 1B (PTP1B) by binding to the active site of PTP1B [177]; deep sea marine fungus *Penicillium solitum* MCCC3A00215-derived viridicatol (**151**) promoted osteoblast stimulated-bone formation and activation of the AKT/glycogen synthase kinase-3 beta signaling pathways [178]; marine gammarid shrimp-derived fungus *Penicillium citrinum* XIA-16 polyketone penicitrinol B (**152**) significantly inhibited ferroptosis in vitro by both lipid peroxidation reduction and HO-1 expression inhibition [179]; marine alga *Ecklonia cava*-derived phloroglucinol (**77**) protected against induction of apoptosis in vitro by Nrf2 stimulation of HO-1 activation [180]; marine fungus *Penicillium janthinellum* SH0301-derived epidithiodiketopiperazine pretrichodermamide B (**153**) inhibited the JAK/STAT3 pathway by directly binding to STAT3, resulting in cell cycle arrest and apoptosis promotion [181]; marine fungus *Phomopsis* sp. FT-0211-derived phenol-containing cytochalasin phenochalasin A (**154**) inhibited macrophage-derived lipid droplet generation in vitro by binding to cellular G-actin [182]; marine bacterium *Streptomyces* sp.-derived angucycline saquayamycin B1 (**155**) induced apoptosis of human colorectal cancer cells by inhibition of the PI3K/AKT signaling pathway [183]; marine brown alga *Sargassum macrocarpum*-derived meroterpenoid sargaquinoic acid (**156**) inhibited angiotensin-1-converting enzyme (ACE) by binding to the ACE active site [184]; marine brown alga *Sargassum serratifolium*-derived sargahydroquinoic acid (**157**) inhibited human basophil degranulation by downregulation of ERK, p38MAPK and NF-κB phosphorylation and translocation [185]; marine brown alga *Sargassum carpophyllun* phlorotannin (**158**) regulated rat basophilic leukemia cells’ release of *β*-hexosaminidase, PGD2 and TNF-α by partial inhibition of Iκb kinase phosphorylation or degradation processes [186]; marine Red Sea soft coral *Sarcophyton glaucum*-derived furanone-based cembranoids (**159**,**160**) protected against indomethacin-induced gastric injury through their mucin-preserving, antioxidant and anti-inflammatory properties [187]; marine sea cucumber *Stichopus japonicus*-derived tetrapeptides (**161**,**162**) enhanced wound healing by accelerating keratinocyte migration and promoting AKT/ERK signaling activation [188]; marine-derived sponge *Stylissa massa* pyrrole imidazole alkaloid (**163**) demonstrated aldose reductase inhibition with a binding power (K_D_) of 6.9 µM [189]; marine soft coral *Sinularia flexibilis*-derived triterpene 11-*epi*-sinulariolide (**164**) induced apoptosis in oral cancer cells in vitro by inhibition of the PI3K/Akt signaling pathway [190]; marine fungus *Penicillium steckii*-derived steckwaic F (**165**) inhibited receptor activator of NF-κB ligand-induced osteoclast differentiation [191]; marine sponge *Rhabdastrella* sp.-derived triterpene stellettin B (**166**) induced apoptosis by activation of the autophagy/DAPK2/apoptosis signaling cascade [192]; marine fungus *Talaromyces* sp. HDN151403-heterodimeric oxaphenalenone talaverrucin A (**167**) rescued zebrafish eyeless phenotype induction by inhibiting the Wnt/*β*-catenin signaling pathway [193]; marine fish *Takifugu flavidus*-derived peptide (**168**) competitively inhibited angiotensin-1-converting enzyme by binding to the active site of the enzyme [194]; marine red sponge *Plakortis lita* thiazine-derived alkaloid thiaplakortone B (**169**) prevented osteoclastogenesis in vitro by inhibiting NF-κB and MAPK signaling and calcium oscillation [195]; marine fungus *Trichoderma* sp. MCCC 3A01244 β-carboline alkaloid trichocarboline A (**170**) inhibited collagen accumulation in vitro by TGF-β/α-smooth muscle actin protein phosphorylation suppression [196]; marine brown alga *Agarum cribrosum*-derived fucol-type phlorotannin trifuhalol A (**171**) reduced lipid accumulation in human primary adipocytes by inhibiting the Wnt/β-catenin and AMPK-dependent signaling pathways [197]; marine fungus *Xylaria* sp.-derived xyloallenoide derivative (**172**) protected against human endothelial progenitor cell senescence, increased anti-aging protein sirtuin type 1 expression and modulated AMPK/Akt signaling pathways [198]; marine fungus *Xylaria* sp.-derived xyloketal B (**173**) reversed nonalcoholic fatty liver disease in vivo by activating the PPARγ signaling pathway [199].

## 5. Reviews on Marine Pharmacology and Pharmaceuticals

In 2022–2023 a number of reviews were published that covered general and/or specific areas of marine preclinical pharmacology: (a) *marine pharmacology and marine pharmaceuticals*: marine natural products and their relevant biological activities [200,201]; pharmacological profiles of marine-derived furanosteroids [202]; natural polyether ionophores and their pharmacological profile [203]; sulfur-containing marine natural products as potential therapeutic agents [204]; therapeutic potential of marine polyphenols [205]; marine chemical ecology research and development of new pharmaceuticals [206]; pharmacological properties of cyanobacterial marine natural products [207,208,209]; therapeutic applications of haloarchaea bioactive molecules [210]; pharmacological potential of brown and red algae [211,212,213]; medical and pharmaceutical applications of algae [214,215,216,217,218]; mangrove-derived natural products and therapeutic applications [219]; marine fungi-derived metabolites with pharmacological activity [220,221,222,223]; marine ascidian natural products for emerging pharmaceutical therapies [224]; marine mollusk-derived natural products and therapeutic potential [225]; marine-sponge-derived secondary metabolites and pharmacological activities [226,227]; sea cucumber saponins’ potential therapeutic activities [228]; bioactive marine natural products from Algoa Bay, South Africa and Western Australia [229,230]; the global marine pharmaceutical pipeline: approved marine-derived compounds and those in Phases I, II and III of clinical development (https://www.marinepharmacology.org/); (b) *antimicrobial, antifungal and antiviral marine pharmacology*: new antimicrobial natural products from the Nation Cancer Institute Program for Natural Product Discovery [231]; pharmacological properties of marine Bacillota (Firmicutes) and Pseudomonadota phyla [232,233]; antimicrobial natural products from marine invertebrates, sponges, fungi and bacteria [234,235,236,237]; microalgal metabolites affecting quorum-sensing-regulated microbial biofilms [238,239]; antimicrobial properties of phloroglucinol and derivatives [240]; antifungal and antibacterial activities of marine natural products [241,242]; anti-candidal marine natural products [243]; recent advances in marine-based antivirals [244,245]; marine natural products targeting dengue, zika, chikungunya and COVID-19 viruses [246,247,248,249]; (c) *antiprotozoal and antimalarial marine pharmacology*: antiprotozoal marine natural products [250]; antiparasitic marine-derived marine natural products [251,252]; antimalarial pharmacology of marine natural products [253,254]; (d) *immuno- and anti-inflammatory marine pharmacology*: anti-allergic and anti-inflammatory marine natural products [255,256]; brown algae phlorotannins’ molecular targets in inflammatory processes [211]; antioxidant properties of marine fungi and sponges [257,258]; octocoral-derived natural products against neutrophilic inflammation [259]; (e) *cardiovascular and antidiabetic marine pharmacology*: marine-derived natural products applied in cardiovascular diseases [260,261,262]; marine-derived omega-3 polyunsaturated fatty acids and polar oils in cardiovascular diseases [263]; seaweed-derived bioactive compounds for cardiovascular disease treatment [264]; antidiabetic marine natural products [265,266]; antidiabetic algal and microalgal metabolites [267,268,269,270,271]; pharmacology of type 2 diabetes mellitus by marine invertebrate-derived natural products [272]; (f) *nervous system marine pharmacology*: bryostatin-1 and promising invertebrate-derived marine natural products for neurological disorders [273,274,275]; astaxanthin for brain aging and adult neurogenesis [276]; marine bioactive natural products for treatment of neurological disorders [277,278,279]; marine natural products for Parkinson’s disease therapeutics [280,281]; marine natural products against Alzheimer’s disease: past, present and future [282]; marine natural products for eye diseases [283]; pharmacology of conotoxins and bibliometric review of the literature of 2000–2022 [284,285,286]; tetrodotoxin for treatment of cancer-related pain [287]; (g) *miscellaneous uses, molecular targets, and methodologies*: marine-derived products for human vision [283]; marine peptides as anti-aging drugs [288,289]; omega-3 fatty acids and potential efficacy in sarcopenia [290]; marine compounds for treatment of skin and soft tissue infections [291]; marine natural product enzyme inhibitor discovery by computational technologies [292]; astaxanthin modulation of signal transduction pathways [293,294]; marine sponge-derived leucettinibs as DYRK/CLK kinase inhibitors [295]; marine invertebrate-derived metalloaminopeptidase inhibitors [296]; mechanism of action of actin-interacting amphidinolides [297]; enzyme inhibitors from marine gorgonians and soft corals [298]; marine natural products as peroxisome proliferator-activated receptor inhibitors [299]; marine natural products as proteasome inhibitors [300]; marine natural products as TRPV1 channel blockers [301]; nanotechnology for the delivery of marine natural products [302]; marine toxins with potential for drug discovery [303].

## 6. Conclusions

This review covering the peer-reviewed marine pharmacology literature published in 2022–2023 is the 14th contribution to the marine *preclinical* pharmacology pipeline review series that was initiated by AMSM in 1998 [1,2,3,4,5,6,7,8,9,10,11,12,13]. This marine pharmacology review series consolidates selected peer-reviewed preclinical marine pharmacological literature published during 2022–2023. The published preclinical marine pharmacology with structurally characterized marine natural compounds involved mechanism-of-action studies by chemists and pharmacologists from 40 countries, namely, Armenia, Australia, Belgium, Botswana, Brazil, Canada, China, Croatia, Czech Republic, Egypt, France, Germany, India, Ireland, Italy, Japan, Jordan, Kuwait, Libya, Luxembourg, Malaysia, Mexico, the Netherlands, Norway, Papua New Guinea, Philippines, Portugal, Russian Federation, Saudi Arabia, South Korea, Spain, Sri Lanka, Switzerland, Thailand, Taiwan, Tunisia, Turkiye, the United Kingdom, Vietnam, and the United States of America (Figure 1). Thus, during 2022–2023 the marine *preclinical* pharmaceutical pipeline provided novel marine chemical leads for the marine *clinical* pharmaceutical pipeline. As currently shown on the marine pharmaceutical pipeline website (https://www.marinepharmacology.org/, accessed on 1 March 2026) there are currently 17 marine-derived pharmaceuticals that have been approved for clinical use by either the U.S. Food and Drug Administration, Australia, Japan, China or Europe, and 29 compounds in either Phase I, II or III of *clinical* pharmaceutical development.

## Figures and Tables

**Table 1:** Marine pharmacology in 2022–2023: mechanism-of action-studies with marine compounds demonstrating antibacterial, antituberculosis, antifungal, antiprotozoal and antiviral activities.

Drug Class	Compound/Organism ^a^	Chemistry	Pharmacologic Activity	IC_50_ ^b^	MMOA ^c^	Country ^d^	References
Antibacterial	Acruciquinone A (**1**)/fungus	Polyketide ^e^	*Staphylococcus aureus* inhibition	>50 µM ^+^	Urease and sortase A inhibition	RUS	[15]
Antibacterial	Alterlactone 5′-*O*-sulfate (**2**)/ fungus	Polyketide ^e^	*S. aureus* inhibition	62.5 μg/mL ^+^	Cell membrane damage	CHN, IND	[16]
Antibacterial	Anisaxin-2S & -2P (**3** & **4**)/ nematode	Peptide ^f^	*Klebsiella pneumoniae* and *Escherichia coli* inhibition	0.25–0.5 µM ^+^	Cell membrane damage	CZE, HRV, ITA, NLD	[17]
Antibacterial	Bisaprasin (**5**)/sponge	Peptide ^f^	*Pseudomonas aeruginosa* quorum sensing inhibition	2.41–3.53 µM	Elastase inhibition	AUS, BWA, GBR, SGP	[18]
Antibacterial	Capitellacin (**6**)/worm	Peptide ^f^	*E. coli* and *Acinetobacter baumanii* inhibition	0.25–0.5 µM ^+^	Cell membrane damage	RUS	[19]
Antibacterial	Equisetin (**7**)/fungus	Alkaloid ^f^	*S. aureus* inhibition	4 μg/mL *	Autophagy activation	CHN	[20]
Antibacterial	5′-epiequisetin (**8**)/fungus	Alkaloid ^f^	*Vibrio* sp. inhibition	86 μg/mL ^+^	Cell membrane damage	CHN	[21]
Antibacterial	Fucoxanthin (**9**)/alga	Terpenoid ^g^	Sepsis inhibition in vivo	1 mg/kg/day	IRF3 activation inhibition	CHN	[22]
Antibacterial	*Heteromastus filiformis* peptide (**10**)/worm	Peptide ^f^	*E. coli* inhibition	0.125–1 µM ^+^	Cell membrane damage	CHN, RUS	[23]
Antibacterial	Lanthelliformisamine C (**11**)/sponge	Alkaloid ^f^	*P. aeruginosa* inhibition	53.1 μg/mL ^+^	Efflux pump inhibition, biofilm inhibition	AUS	[24]
Antibacterial	Lulworthinone (**12**)/fungus	Polyketide ^e^	*Bacillus subtilis* 168 inhibition	8 μg/mL *	Cell membrane damage	NOR	[25]
Antibacterial	Myristic and oleic acid (**13**,**14**)/sponge	Fatty acid ^i^	MRSA and MSSA inhibition	6.25 μg/mL ^+^	Gene transcription downregulation	FRA, IRL	[26]
Antibacterial	*Olleya marilimosa* fatty acid (**15**)/bacterium	Fatty acid ^i^	*B. subtilis* inhibition	32 μg/mL ^+^	Cell membrane damage	USA	[27]
Antibacterial	Penicilazaphilone C (**16**)/ fungus	Polyketide ^e^	*E. coli* inhibition	8 μg/mL ^+^	Aerobic and anaerobic respiration inhibition	CHN	[28]
Antibacterial	Prodigiosin (**17**)/bacterium	Alkaloid ^f^	*Cutibacterium acnes* inhibition	25 μg/mL ^+^	Altered metabolism and increased SigB	S. KOR	[29]
Antibacterial	Resistoflavin (**18**)/bacterium	Polyketide ^e^	*S. aureus* and *E. coli* biofilm inhibition	200 μg/mL ^+^	Binding to biofilm-forming protein	EGY	[30]
Antibacterial	Trypacidin (**19**)/fungus	Polyketide ^e^	*Vibrio parahaemolyticus* inhibition	31.2 μg/mL ^+^	Cell membrane damage	CHN	[31]
Antituberculosis	Gliotoxin (**20**)/fungus	Polyketide ^e^	*Mycobacterium tuberculosis* H37Rv inhibition	0.5 μM *	Autophagy inhibition	CHN	[32]
Antifungal	Bacillimide (**21**)/bacterium	Alkaloid ^f^	*Candida albicans* growth inhibition	44.24 μM	Icl mRNA inhibition	S. KOR	[33]
Antifungal	Cocultimycin A (**22**)/fungus	Polyketide ^e^	*C. albicans* growth inhibition	1.56 μg/mL	Cell wall and membrane damage	CHN	[34]
Antifungal	Dehydrocurvularin (**23**)/fungus	Polyketide ^e^	*C. albicans* growth inhibition	62.5 μg/mL *	Hyphal adhesion morphogenesis gene inhibition	JPN	[35]
Antifungal	2,4-di-*tert*-butylphenol (**24**)/ bacterium	Shikimate ^h^	*Fusarium foetens* hyphal growth inhibition	30 μg/mL	Cell membrane damage	IND	[36]
Antifungal	Isaridin J (**25**)/fungus	Peptide ^f^	*Geotrichum citri-aurantii* inhibition	56.8 μg/mL	Cell membrane damage	CHN	[37]
Antiprotozoal	*Halimeda macroloba* halogenated compound (**26**)/alga	Polyketide ^e^	*Plasmodium falciparum* inhibition	3.2 µg/mL	Cytochrome-C reductase binding	DEU, EGY, SAU, TUN	[38]
Antiprotozoal	(+)-elatol (**27**)/alga	Terpenoid ^g^	*Naegleria fowleri* trophozoite inhibition	1.08 μM	Programmed cell death induction	BRA, ESP	[39]
Antiprotozoal	Gallinamide A (**28**)/ cyanobacterium	Peptide ^f^	*Trypanosoma cruzi* amastigote inhibition	14.7 nM	Cruzain inhibition	AUS, BRA, USA	[40]
Antiprotozoal	*Gongolaria abies-marina* meroterpenoids (**29**, **30**, **31**)/alga	Terpenoid ^g^	*T. cruzi* and *Leishmania amazonensis* inhibition	3.2–4.9 μM	Programmed cell death induction	ESP, MEX	[41]
Antiprotozoal	*G. abies-marine* meroterpenoids (**29**, **30**, **31)** alga	Terpenoid ^g^	*N. fowleri* inhibition	13–22 μM	Programmed cell death induction	ESP	[42]
Antiprotozoal	Laurequinone (**32**)/alga	Terpenoid ^g^	*L. amazonensis* inhibition	1.9 μM	Programmed cell death induction	ESP, MEX	[43]
Antiviral	Ansellone J (**33)**/sponge	Terpenoid ^g^	HIV latency reversal stimulation	1 μM *	Proposed binding to PKC	CAN, NLD, USA	[44]
Antiviral	Aurasperone A (**34**)/fungus	Polyketide ^e^	SARS-CoV-2 inhibition	12.25 μM	M^pro^ protease inhibition	EGY	[45]
Antiviral	Bacillibactin (**35**)/fungus	Alkaloid ^f^	N1-type NA inhibition	1.92 μM	NA catalytic pocket binding	CHN	[46]
Antiviral	Ishophloroglucin A (**36**)/alga	Shikimate ^h^	SARS-CoV-2 3CL^pro^ and PL^pro^ inhibition	0.48, 1.4 μM	Competitive enzyme inhibition	CAN, S. KOR	[47]
Antiviral	Janthinellumnines A & B (**37**, **38**)/fungus	Terpenoid ^g^	Influenza virus inhibition	3.8–15.7 μM	Neuraminidase inhibition	CHN	[48]
Antiviral	Lamellarin α 20-sulfate (**39**)/ ascidian	Alkaloid ^f^	Ebola and SARS-CoV-2 inhibition	150 μM *	Viral endosome uncoating inhibition	JPN	[49]
Antiviral	*Micromonospora* sp. peptide A-3302-B (**40**)/bacterium	Peptide ^f^	HSV-2 inhibition	14 μM	HSV-2 cell egress inhibition	CHE, ITA, THA,	[50]
Antiviral	Neoechinulin A (**41**)/fungus	Alkaloid ^f^	SARS-CoV-2 protease inhibition	0.47 μM	Viral replication inhibition	EGY, SAU, USA	[51]
Antiviral	Thorectidiol A (**42**)/sponge	Terpenoid ^g^	SARS-CoV-2 spike protein inhibition	1 μM	Inhibition of Spike RBD interaction with host ACE2	CAN, NLD, PNG, USA,	[52]
Antiviral	Tuaimenal A (**43**)/soft coral	Terpenoid ^g^	SARS-CoV-2 inhibition	21 μM	3CL^pro^ protease inhibition	IRL, USA	[53]

**^a^** **Organism**: *Kingdom Animalia*: worm (Phylum Annelida); ascidian (Phylum Chordata); coral (Phylum Cnidaria); nematode (Phylum Nematoda); sponge (Phylum Porifera); *Kingdom Monera*: bacterium, cyanobacterium (Phylum Cyanobacteria); *Kingdom Fungi*: fungus; *Kingdom Plantae*: alga. **^b^ IC_50_**: concentration of a compound required for 50% inhibition in vitro; *: estimated IC_50_; ^+^ MIC: minimum inhibitory concentration. **^c^** **MMOA**: molecular mechanism of action. **^d^** **Country**: AUS: Australia; BWA: Botswana; BRA: Brazil; CAN: Canada, CHE: Switzerland; CHN: China; CZE: Czech Republic; DEU: Germany; EGY: Egypt; ESP: Spain; FRA: France; GBR: United Kingdom; HRV: Croatia; IND: India; IRL: Ireland; ITA: Italy; JPN: Japan; MEX: Mexico; NLD: Netherlands; NOR: Norway; PNG: Papua New Guinea; RUS: Russia; SAU: Saudi Arabia; SGP: Singapore; S. KOR: South Korea; THA: Thailand; TUN: Tunisia; USA: United States of America. **Chemistry:** **^e^** polyketide; **^f^** nitrogen-containing compound; **^g^** terpene; **^h^** shikimate; **^i^** fatty acid. **Abbreviations:** HIV-1: human immunodeficiency virus type-1; icl: isocitrate lyase; IRF3: interferon regulatory factor 3; MRSA: methicillin-resistant *Staphylococcus aureus*; MSSA: methicillin-susceptible *Staphylococcus aureus*.

**Table 2:** Marine pharmacology in 2022–2023: mechanism-of-action studies with marine compounds with antidiabetic and anti-inflammatory activity and affecting the immune and nervous system.

Drug Class	Compound/Organism ^a^	Chemistry	Pharmacological Activity	IC_50_ ^b^	MMOA ^c^	Country/ Region ^d^	References
Antidiabetic	Alternariol 1′-hydroxy-9-methyl ether (**44**)/fungus	Polyketide ^e^	α-glucosidase inhibition	6.27 µM	Mixed-type inhibition	CHN	[54]
Antidiabetic	Asperflagin (**45**)/fungus	Terpenoid ^f^	Glucose uptake stimulation in vitro	20 µM *	PPAR-γ partial agonist	S. KOR	[55]
Antidiabetic	Avarone (**46**)/sponge	Terpenoid ^f^	PTP1B and AKR1B1 inhibition	6.7; 0.08	PTP1B competitive inhibition	ITA, TUR	[56]
Antidiabetic	Brasilterpenes A (**47**) & C (**48**)/alga	Terpenoid ^f^	Diabetic zebrafish hypoglycemic activity	10 µM *	Gluconeogenesis suppression	CHN	[57]
Antidiabetic	Cladopsol B (**49**) fungus	Terpenoid ^f^	Glucose uptake stimulation in vitro	20 µM *	PPAR-γ partial agonist	CHN, S. KOR	[58]
Antidiabetic	Dieckol (**50**)/alga	Polyketide ^e^	In vitro decrease in AGE formation	5 µM *	Decreased RAGE protein expression	S. KOR	[59]
Antidiabetic	Echinochrome A (**51**)/sea urchin	Polyketide ^e^	Prevention of murine diabetic nephropathy	3 mg/kg/day **	PKCi/p38 MAPK signaling inhibition	VNM, RUS	[60]
Antidiabetic	Eurothiocin D (**52**)/fungus	Shikimate ^h^	α-glucosidase inhibition	5.4 µM	Docking studies completed	CHN	[61]
Antidiabetic	(−)-jasmonic acid (**53**)/ bacterium	Polyketide ^e^	α-amylase and α-glucosidase inhibition	0.29 mM	Docking studies completed	VNM	[62]
Antidiabetic	Zonarol (**54**)/alga	Terpenoid ^f^	α-glucosidase inhibition	6.03 mg/mL	Competitive-type inhibition kinetics	VNM	[63]
Anti-inflammatory	Acteoside (**55**)/mangrove	Shikimate ^h^	Murine ulcerative colitis inhibition through decreased IL-1, TNF-α, and increased IL-10	100 mg/kg **	JAK/STAT, iNOS and NF-kB signaling inhibition	CHN	[64]
Anti-inflammatory	Aeroplysinin-1 (**56**)/sponge	Nitrogen-containing ^g^	IL-6 expression decrease	3 µM *	NF-kB, IKK and Akt inhibition	ESP	[65]
Anti-inflammatory	Tricycloalternarene O and P (**57** and **58**)/fungus	Terpenoid ^f^	Macrophage NO and IL-6 inhibition	14–19 μM	NF-kB inhibition	CHN	[66]
Anti-inflammatory	Aspersterol C (**59**)/fungus	Terpenoid ^f^	Macrophage NO and IL-6 inhibition	11.6 μM	IL-6 and iNOS expression decrease	S. KOR	[67]
Anti-inflammatory	Astaxanthin (**60**)/shrimp	Terpenoid ^f^	Macrophage IL-1β release inhibition	10 μM *	SDH-HIF-1-α axis inhibition	JPN	[68]
Anti-inflammatory	Aszonalenin (**61**)/fungus	Alkaloid ^g^	HUVEC NO, TNF-α, IL-6 inhibition	>10 μM *	MAPK and PI3K/AKT pathway phosphorylation inhibition	CHN	[69]
Anti-inflammatory	Brevetoxin analogs (**62** and **63**)/dinoflagellate	Polyketide ^e^	Upregulation of THP-1 monocyte M1/M2 markers	1 μM *	Apoptosis and necrosis induction	USA	[70]
Anti-inflammatory	5-chloro-6-hydroxymellein (**64**)/fungus	Polyketide ^e^	Macrophage NO, TNF-α, IL-6 inhibition	10 μM *	PI3K/AKT pathway inhibition	CHN	[71]
Anti-inflammatory	Cyanobacterial fatty acid (**65**)/cyanobacterium	Polyketide ^e^	Macrophage NO inhibition	3.2 μM *	iNOS and Nqo1 expression decrease	CHN, KWT, USA	[72]
Anti-inflammatory	Dysidazirine carboxylic acid (**66**)/cyanobacterium	Terpenoid ^f^	Macrophage NO inhibition	50 μM	iNOS expression decrease	BRA, USA	[73]
Anti-inflammatory	Dysambiol (**67**)/sponge	Terpenoid ^f^	Macrophage TNF-α, IL-1β, IL-6 inhibition	5 μM *	Decreased NF-kB and MAPK activation	CHN	[74]
Anti-inflammatory	Eckmaxol (**68**)/alga	Polyketide ^e^	Macrophage NO, PGE_2_, IL-1β, IL-6, and TNF-α inhibition	>42 μM *	Decreased NF-kB/JNK activation	CAN, S. KOR	[75]
Anti-inflammatory	Eschscholin B (**69**)/fungus	Polyketide ^e^	Macrophage NO inhibition	19.3 μM	Decreased NF-kB and MAPK activation	CHN	[76]
Anti-inflammatory	Fucoxanthin (**9**)/alga	Terpenoid ^f^	Attenuation of liver cell IL-1, IL-6, and TNF-α	0.5 µg/mL *	Increased NRF2 and AMPK signaling	CHN	[77]
Anti-inflammatory	Fucoxanthin (**9**)/alga	Terpenoid ^f^	Inhibition of hepatic IL-1β, IL-6 and TNF-α	25 μM *	Activation of PI3K/Akt/NRF2 signaling	EGY, JOR, SAU, TUN	[78]
Anti-inflammatory	Fucoxanthin (**9**)/alga	Terpenoid ^f^	Inhibition of IR-induced macrophage IL-1β and TNF-α	5 μM *	Enhanced Sirt1 expression	S. KOR	[79]
Anti-inflammatory	Ishigoside (**70**)/alga	Terpenoid ^f^	HaCaT cell MMP, IL-6, and IL-8 production inhibition	50 μM *	Decreased MAPK, AP-1, and NF-kB activation	CHN	[80]
Anti-inflammatory	Littoreanoid K (**71**)/fungus	Terpenoid ^f^	Macrophage NO, IL-6, and TNF-α inhibition	19.02 μM	iNOS and COX-2 expression decrease	CHN	[81]
Anti-inflammatory	LMWCP (**72**)/fish	Peptide ^g^	Inflammation suppression in rat osteoarthritis	400 mg/kg **	Decreased serum IL-1β, IL-6, and TNF-α	S. KOR	[82]
Anti-inflammatory	MCDO (**73**)/diatom	Terpenoid ^f^	Macrophage NO, IL-1β and PGE_2_ inhibition	>12.5 μM *	iNOS and COX-2 expression decrease	LKA, S. KOR	[83]
Anti-inflammatory	Ovothiol (**74**)/sea urchins	Alkaloid ^g^	IL-6, IL-8, and TNF-α inhibition from ex vivo skin tissue	2 μM *	ERK and JNK phosphorylation inhibition in HaCaT cell line	ESP, ITA, PRT	[84]
Anti-inflammatory	1-*O*-alkyl-glycerols (**75**)/squid	Fatty Acids ^i^	Improved obese asthma patients’ lung function and plasmalogen synthesis	0.4 g/day **	Decreased plasma levels of LTB4 and TXB2	RUS	[85]
Anti-inflammatory	Phlorofucofuroeckol A (**76**)/alga	Shikimate ^h^	HaCaT cell inflammatory cytokine and chemokine inhibition	31.3 μM *	NF-kB and MAPK inhibition	S. KOR	[86]
Anti-inflammatory	Phloroglucinol (**77**)/alga	Shikimate ^h^	Macrophage NO, IL-1β, IL-6, TNF-α and PGE_2_ inhibition	>10 μM *	Activation of Regulation of AMPK/Nrf2 /HO-1 signaling	S. KOR	[87]
Anti-inflammatory	Phomotone A (**78**)/fungus	Polyketide ^e^	Macrophage NO inhibition	10 μM	iNOS and COX-2 expression decrease	CHN	[88]
Anti-inflammatory	Prostaglandin A_2_ (**79**)/soft coral	Fatty Acids ^i^	Macrophage NO and IL-6 inhibition	3.2, 9 μM	Decreased NF-kB activation	JPN	[89]
Anti-inflammatory	Pseudoviridinutan F (**80**)/ fungus	Peptide ^g^	Macrophage NO inhibition	20 μM *	iNOS expression decrease	CHN	[90]
Anti-inflammatory	Sargachromanol (**81**)/alga	Terpenoid ^f^	Particulate matter-induced macrophage cytokine, PGE_2_ and NO inhibition	15.6 µM *	NF-kB, and MAPK inhibition	JPN, S. KOR, LKA	[91]
Anti-inflammatory	Sargasilol A (**82**)/alga	Terpenoid ^f^	Microglia NO, IL-1, IL-6, and TNF-α inhibition	2 μM	NF-kB signaling inhibition	CHN	[92]
Anti-inflammatory	Somalactam A (**83**)/ bacterium	Polyketide ^e^	Macrophage IL-6 and TNF-α release inhibition	0.2, 5.7 μM	MAPK phosphorylation inhibition	CHN, USA	[93]
Anti-inflammatory	Streptinone (**84**)/ bacterium	Shikimate ^h^	Macrophage NO and PGE_2_ inhibition	5 μM *	Decreased NF-kB activation	S. KOR	[94]
Anti-inflammatory	Streptoglyceride F (**85**)/ bacterium	Polyketide ^e^	Macrophage NO and IL-6 inhibition	20 μM *	Decreased ERK, JNK, p38K activation	S. KOR	[95]
Anti-inflammatory	Variotin B (**86**)/fungus	Terpenoid ^f^	Macrophage NO and IL-6 inhibition	20 μM	IL-6 and iNOS expression decrease	S. KOR	[96]
Anti-inflammatory	*Neopetriosia compacta* xestoquinone analogs (**87** and **88**)/sponge	Polyketide ^e^	Macrophage NO release inhibition	2.5, 4 μM	Nrf2 activation	PHL	[97]
Anti-inflammatory	Xinghamide A (**89**)/ bacterium	Peptide ^g^	Macrophage NO inhibition	100 μM *	COX-2 expression decrease	S. KOR	[98]
Immune system	Assimiloside A (**90**)/sponge	Fatty Acids ^i^	Murine macrophage ROS increase	0.01 µM *	Lysosomal activity stimulation	RUS	[99]
Immune system	*Cyclina sinensis* peptide (**91**)/mollusk	Peptide ^g^	Increased IL-1β, IL-6, and TNF-α in immunosuppressed mice	50 mg/kg **	Increased MAPK/NF-kB and PI3K/AKT activation	CHN	[100]
Immune system	DHB (**92**)/alga	Polyketide ^e^	IgE/BSA-stimulated mast cells degranulation and cytokine inhibition	31.3 µM *	NF-kB and IL-4, IL-5, IL-6, IL-13, and TNF-α decrease	S. KOR	[101]
Immune system	Echinochrome A (**51**)/ sea urchin	Polyketide ^e^	Reduction of IgE, IL-4 and IL-1β	0.1 mg/kg **	Regulation of Keap1/Nrf2 signaling	EGY, LBY, SAU	[102]
Immune system	Eckol (**93**)/alga	Polyketide ^e^	Inhibition chronic murine ulcerative colitis	0.5–1.0 mg/kg	Downregulation of TLR4/NF-kB/STAT3 pathway	CHN	[103]
Nervous system	Aaptamine (**94**)/sponge	Alkaloid ^g^	BChE and AChE inhibition	4.6 µM	Mixed-type inhibition	CHN	[104]
Nervous system	Aaptamine (**94**)/sponge	Alkaloid ^g^	Alleviates rat neuropathic pain	30 µg/day **	VEGF and LDHA downregulation	TWN	[105]
Nervous system	*Anemonia sulcata* AsKC11 (**95**)/sea anemone	Peptide ^g^	Enhanced K+ currents in oocyte-expressing GIRK1/2 channels	81 µM *	Inactive at Kv1.1-1.4 channels	AUS, BEL, DEU, NOR, RUS	[106]
Nervous system	Amphichoterpenoid E (**96**)/fungus	Terpenoid ^f^	AChE inhibition	11.6 µM	PAS-binding determined by docking analysis	CHN	[107]
Nervous system	Antillatoxin (**97**)/ cyanobacterium	Peptide ^g^	Neurite outgrowth stimulation and BDNF release	30 nM	NMDAR-BDNF-TrkB-dependent mechanism	CHN, USA	[108]
Nervous system	Asperpendoline (**98**)/ fungus	Alkaloid ^g^	Reduction of human neuroblastoma ROS release	50 µM *	Regulation of Keap1/Nrf2 signaling	CHN	[109]
Nervous system	Astaxanthin (**60**)/shrimp	Terpenoid ^f^	Reduction of ischemia-induced hippocampal neuronal loss	100 mg/kg **	Increased SOD expression	S. KOR	[110]
Nervous system	Astaxanthin (**60**)/shrimp	Terpenoid ^f^	AChE inhibition and enhanced SOD activity	8.64 µM	Reversible and competitive inhibition	CHN	[111]
Nervous system	Astaxanthin (**60**)/shrimp	Terpenoid ^f^	Amelioration of zebrafish cognitive dysfunction	10–30 mg/mL **	MMP-13 and AChE inhibition	IND, MYS, SAU, USA	[112]
Nervous system	Aurasperone F (**99**)/fungus	Polyketide ^e^	Dose-dependent Aβ aggregation inhibition	8.1 µM	BACE1 inhibition	JPN	[113]
Nervous system	Butyrolactone (**100**)/fungus	Mixed	Amelioration of AlCl_3_-induced zebrafish cognitive deficits	25–100 mg/kg **	Decreased pro-inflammatory cytokines	CHN	[114]
Nervous system	Brevetoxin-2 (**62**)/dinoflagellate	Polyketide ^e^	Neurite outgrowth stimulation	30 nM	GluN2B-NMDAR and PAK1 pathway	USA	[115]
Nervous system	*Conus quercinus* conotoxin QuIA (**101**)/cone snail	Peptide ^g^	α3β2 nAChR inhibition	0.0056 µM	α6/α3β4 nAChR high-affinity binding	CHN	[116]
Nervous system	SIIID conotoxin (**102**)/cone snail	Peptide ^g^	Human α7 nAChR inhibition	1 µM	Reversible inhibition	CHN	[117]
Nervous system	Copteremophilane G (**103**)/fungus	Terpenoid ^f^	PC12 neurons increased viability	20 µM *	MDA levels reduced	CHN	[118]
Nervous system	*Dicyota coriacea* xenicane diterpene (**104**)/alga	Terpenoid ^f^	Antioxidant protection to PC12 neurons	2.5 µM *	Nrf2/ARE pathway activation	CHN	[119]
Nervous system	Decanoic acid (**105**)/sea cucumber	Fatty Acids ^i^	Neuroprotection of *C. elegans* parkinsonism in vivo model	5–25 µg/mL *	Reduction of intracellular ROS	THA	[120]
Nervous system	Dehydroshearinine A (**106**)/fungus	Alkaloid ^g^	Reduction of PC12 neuronal apoptosis	100 µM *	Downregulation PI3K/AKT pathway	CHN	[121]
Nervous system	Echinochrome A (**51**)/sea urchin	Polyketide ^e^	TRPV3 and Orai1 channel inhibition	2.1, 2.4 µM	TREK-1 & TRAAK facilitation	RUS, S. KOR	[122]
Nervous system	*Stychopus japonica* peptide (**107**)/sea cucumber	Peptide ^g^	Attenuation of murine cognitive impairment	0.3–1.2 mg/day/ mouse **	Cholinergic dysfunction reduction	CHN	[123]
Nervous system	Fucoxanthin (**9**)/alga	Terpenoid ^f^	Reduced L-DOPA-induced neurotoxicity	100 mg/kg/day **	ROS expression suppression and ERK/JNK-c-Jun inhibition	CHN	[124]
Nervous system	Fucoxanthin (**9**)/alga	Terpenoid ^f^	AChE inhibition and enhanced oxidative stress protection	130 µg/mL	Regulation of PI3K/AKT pathway	IND, VNM	[125]
Nervous system	*Heteractis magnifica* peptide (**108**)/sea anemone	Peptide ^g^	Inhibition of human ASIC ion channels	14.1, 14.6 µM	In vivo analgesic and anti-inflammatory	RUS	[126]
Nervous system	*H. magnifica* peptide (**109**)/sea anemone	Peptide ^g^	Reduction of Neuro-2a intracellular ROS induction	0.01 µM *	ATP-induced P2X7R activation inhibited	ARM, BEL, RUS	[127]
Nervous system	*Metridium senile* peptide (**110**)/sea anemone	Peptide ^g^	In vivo murine hyperalgesia modulation	0.3 mg/kg **	TRPA1 channel activation	RUS	[128]
Nervous system	Palmitic acid (**111**)/sea cucumber	Fatty Acids ^i^	Attenuation of *C. elegans* neurodegeneration	5 µg/mL	Antioxidant gene upregulation	THA	[129]
Nervous system	Phlorofucofuroeckol A (**76**)/alga	Polyketide ^e^	AChE and BChE inhibition	4.4, 6.4 µM	Mixed inhibition mode	S. KOR	[130]
Nervous system	Sargachromanol (**81**)/alga	Terpenoid ^f^	Attenuation of glutamate-induced neuronal cell death	7.4 µM *	ROS and p38MAPK/JNK phosphorylation decrease	S. KOR	[131]
Nervous system	Territrem (**112**)/fungus	Mixed	Neocortical murine neurons SCO inhibition	15 µM	Inhibition of 4-AP triggered SCO	CHN	[132]

**^a^** **Organism**: *Kingdom Animalia*: fish (Phylum Chordata), shrimp (Phylum Arthropoda); coral, sea anemone (Phylum Cnidaria); sea cucumber, sea urchin (Phylum Echinodermata); cone snail, squid, mollusk (Phylum Mollusca); sponge (Phylum Porifera); *Kingdom Chromista*: dinoflagellate; *Kingdom Fungi*: fungus; *Kingdom Plantae*: alga; diatoms, mangrove; *Kingdom Monera*: bacterium; cyanobacterium (Phylum Cyanobacteria). **^b^** **IC_50_**: concentration of a compound required for 50% inhibition; *: apparent IC_50_; ** in vivo study. **^c^** **MMOA:** molecular mechanism of action. **^d^** **Country/Region:** ARM: Armenia; AUS: Australia; BEL: Belgium; BRA: Brazil; CAN: Canada; CHN: China; DEU: Germany; EGY: Egypt; ESP: Spain; IND, India; ITA: Italy; JOR: Jordan; JPN: Japan; KWT: Kuwait; LBY: Libya; LKA: Sri Lanka; MYS: Malaysia; NOR: Norway; PHL: Philippines; PRT: Portugal; RUS: Russia; SAU: Saudi Arabia; S. KOR: South Korea; THA: Thailand; TUN: Tunisia; TUR: Turkiye; TWN: Taiwan; USA: United States of America; VNM: Vietnam. **Chemistry:** **^e^** polyketide; **^f^** terpene; **^g^** nitrogen-containing compound; **^h^** shikimate; **^i^** fatty acid. **Abbreviations:** 4-AP: 4-aminopyridine; Aβ: amyloid-β peptide; Ach: acetylcholine; AChE: acetylcholinesterase; AGE: advanced glycation end products; AKR1B1: aldose reductase; AKT: protein kinase B; AMPK: 5′ AMP-activated protein kinase; AP-1: dimeric transcription factor; ASIC: acid-sensing ion channel; ATP: adenosine triphosphate; BChE: butyrylcholinesterase; BDB: 3-Bromo-4,5-Dihydroxybenzaldehyde; Akt: also known as protein kinase B, a serine/threonine protein kinase; BACE1: β-Secretase; BDNF: brain-derived neurotrophic factor; BSA: bovine serum albumin; COX: cyclooxygenase; ERK: extracellular signal-regulated kinase; FFA: free fatty acid; GIRK: G protein-coupled inwardly rectifying potassium channels; HaCaT: human keratinocyte cell line; HIF-1-α: hypoxia-inducible factor-1-α; HO-1: heme oxygenase-1 protein; HUVEC: human umbilical vein endothelial cells; IgE: Immunoglobulin E; IKK: IkappaB kinase; IL: interleukin; iNOS: inducible nitric oxide synthase; IR: ionizing radiation; JAK: Janus kinase; JNK: c-jun N-terminal kinase; Keap1: Kelch-like ECH-associated protein 1; Kv: voltage-gated potassium channel; LDHA: lactate dehydrogenase A; L-DOPA: L-3,4-dihydroxyphenylalanine; LPS: lipopolysaccharide; LTB4: leukotriene B4; MAPK: mitogen-activated protein kinase; MMP: matrix metalloproteinase; nAChR: nicotinic acetylcholine receptor; MCDO: 24-Methylcholesta-5(6), 22-Diene-3β-ol; MDA: malondialdehyde; NAFLD: nonalcoholic fatty liver disease; NF-κB: nuclear factor kappa-light-chain-enhancer of activated B cells; NMDAR: Glutamate N-methyl-D-aspartate receptor; NO: nitric oxide; Nq01: NADPH quinone dehydrogenase 1; Nrf2-ARE: nuclear transcription factor E2-related factor antioxidant response element; Orai1: calcium release-activated calcium channel protein 1; P2X7R: ATP-sensitive cation P2X7 receptor; PAK: p21-Activated kinase 1; PAS: peripheral anionic site; PBMC: PGE_2_: prostaglandin E_2_; PI3K: phosphoinositide 3-kinase; PK: protein kinase; PPAR-γ: peroxisome proliferator-activated receptor ligand; PTP1B: tyrosine phosphatase 1B; p38: p38 mitogen-activated protein kinases; RAGE: receptor for AGE protein; ROS: reactive oxygen species; SCO: synchronous Ca^2+^ oscillations; SDH: succinate dehydrogenase; SIRT1: Sirtuin1; SOD: superoxide dismutase; STAT: signal transducer and activator of transcription; TLR4: toll-like receptor 4; TNF-α: tumor necrosis factor-α; TRAAK: mammalian neuronal mechano-gated K+ channel; TREK-1: potassium channel subfamily K member 2; TrkB: tropomyosin-related kinase B; TRPA1: transient receptor potential ankyrin 1; TRPV: transient receptor potential vanilloid channel; TXB2: thromboxane B2; VEGF: vascular endothelial growth factor.

**Table 3:** Marine pharmacology in 2022–2023: marine compounds with miscellaneous mechanisms of action.

Compound/Organism ^a^	Chemistry	Pharmacological Activity	IC_50_ ^b^	MMOA ^c^	Country/ Region ^d^	References
*A. suberitoides* aaptamine (**113**)/sponge	Alkaloid ^g^	Cell cycle G1 period inhibition	3 µg/mL *	Phospho-CDK2 protein inhibition	CHN	[133]
ADAT (**114**)/alga	Polyketide ^e^	Autophagy inhibition	10 μM *	mTOR, p70S6K, FoxO3a signaling activation	CHN	[134]
Ageladine A derivative (**115**)/sponge	Alkaloid ^g^	HUVEC migration and tube formation inhibition	6.25 μM *	Sirtuin 6 inhibition	CHN	[135]
(−)-agelasidine A (**116**)/sponge	Terpenoid ^f^	Apoptosis induction	70 μM *	ER stress-related protein upregulation	TWN	[136]
AME1 (**117**)/alga	Terpenoid ^f^	Hepatic cholesterol and TG reduction	1 μM *	PPAR-α activation	VNM	[137]
Anserine (**118**)/fish	Peptide ^g^	Protective effect on TBHP-induced in vitro liver injury	40 mM *	Keap1-Nrf2 signaling regulation	CHN	[138]
Astaxanthin (**60**)/shrimp	Terpenoid ^f^	Blue light-induced ARPE-19 cell apoptosis inhibition	2 μM *	Scavenging ^1^O_2_ directly	JPN	[139]
*Crassostrea hongkongensis* peptide (**119**)/oyster	Peptide ^g^	UVB-induced ROS release inhibition in vitro	20 μM *	MAPK signaling downregulation	CHN	[140]
*C. frondosa* phospholipids (**120**,**121**)/sea cucumber	Fatty acid ^i^	Hepatic steatosis improvement	150 mg/kg **	Regulation hypothalamic PPAR-γ and CD36	CHN	[141]
Cyclotheonellazole A (**122**)/sponge	Peptide ^g^	Acute murine lung injury alleviation	30 mg/kg/day **	Neutrophil elastase inhibition	CHN	[142]
Danthron (**123**)/fungus	Polyketide ^e^	HUVEC angiogenesis inhibition	10 μM *	MMP-2 secretion inhibition	ESP	[143]
Dentithecamides A and B (**124**,**125**)/hydroid	Alkaloid ^g^	PAX3-FOXO1 transcription inhibition	13, 17 μM	Apoptosis and cytokine gene upregulation	CHN, USA	[144]
Deoxyvasicinone (**126**)/bacterium	Alkaloid ^g^	Human and murine melanin synthesis inhibition	125 μM *	TYR, TRP-1 and TRP-2 expression inhibition	S. KOR, USA	[145]
Didemnin B (**127**)/ascidian	Peptide ^g^	tRNA ribosomal A site accommodation inhibition	4.5 nM	eEF1A conformational change inhibition	GBR, USA	[146]
Diaporisoindole B (**128**)/fungus	Alkaloid ^g^	Lipid reduction in human macrophages	5 μM *	PPAR-γ activation and MAPK inhibition	CHN	[147]
Dieckol (**50**)/alga	Polyketide ^e^	Murine UVB-induced skin wrinkling amelioration	5, 10 mg/kg	MMP expression inhibition	S. KOR	[148]
Dihydrotrichodimerol (**129**)/fungus	Polyketide ^e^	Hepatic liver accumulation inhibition in vivo	3 mg/kg	PPAR-γ activation and upregulation of SIRT-1	CHN	[149]
Echinochrome A(**51**)/sea urchin	Polyketide ^e^	Intestinal epithelium regeneration	10 μM *	LGR5 and MUC2 expression increase	RUS, S. KOR	[150]
Echinochrome A (**51**)/sea urchin	Polyketide ^e^	Melanin synthesis inhibition	10 μM *	CREB signaling inhibition	RUS, S. KOR	[151]
FCGFC1 (**130**)/fungus	Alkaloid ^g^	NSCLC PC9 cell line growth inhibition	9, 41 μM	GO/G1 cell cycle arrest and apoptosis	CHN	[152]
FCGFC1 (**130**)/fungus	Alkaloid ^g^	Enhanced fibrinolytic activity	6 μM *	Lower density fibrin clots with pores	CHN	[153]
Fucosterol (**131**)/alga	Terpenoid ^f^	Oxidative stress protection in TNF-α/IFN-γ-stimulated HDF	60 μM *	Nrf2/HO-1 signaling upregulation	S. KOR	[154]
Fucoxanthin (**9**)/alga	Terpenoid ^f^	Reduction of murine atherosclerosis	1.8 µg/day **	PI3K/Akt signaling activation	CHN	[155]
Fucoxanthin (**9**)/alga	Terpenoid ^f^	Lung cancer cell inhibition	17–41 µM	PI3K/Akt signaling inactivation	CHN	[156]
Guisinol (**132**)/fungus	Polyketide ^e^	Osteoclastogenesis inhibition	5 µM *	NF-κB p65 nuclear translocation inhibition	CHN	[157]
10Z-hymenialdisine (**133**)/sponge	Alkaloid ^g^	Angiogenesis inhibition	5 µM *	NF-κB and angiogenic factor inhibition	JPN	[158]
Holothurin A (**134**)/sea cucumber	Terpenoid ^f^	EMT inhibition	0.5 µM *	P38 MAPK signaling inhibition	THA	[159]
Iezoside (**135**)/cyanobacterium	Polyketide ^e^	Increase in cytosolic Ca^2+^ oscillations	0.32 µM	SERCA inhibition	USA	[160]
Indiculide A (**136**)/octopus	Polyketide ^e^	5-lipoxygenase inhibition	2.57 µM	Non-competitive inhibition	IND	[161]
Isaridin E (**137**)/fungus	Peptide ^g^	ADP-induced platelet aggregation inhibition	26.1 μM	P-selectin CD62p decreased expression	CHN	[162]
Isofloridoside (**138**)/alga	Sugar	Taste receptor T1R2/T1R3 activation	10 mM *	Intracellular Ca^2+^ increase	JPN	[163]
Isophloroglucin A (**36**)/alga	Polyketide ^e^	Inhibition of osteoclast differentiation	5 µg/mL	NFATc1 and c-Fos expression inhibition	S. KOR	[164]
Isophloroglucin A (**36**)/alga	Polyketide ^e^	Adipogenesis inhibition	12.5 µM *	PTP1B inhibition	S. KOR	[165]
Insulicolide A (**139**)/ fungus	Terpenoid ^f^	Inhibition of osteoclast formation	1 µM *	c-Fos and NFATc1 translocation inhibition	CHN	[166]
Lagunamide D (**140**)/ cyanobacterium	Peptide ^g^	Mitochondrial dysfunction	>10 nM	VDAC3 gene affected	USA	[167]
*Lophius litulon* peptide (**141**)/fish	Peptide ^g^	ACE inhibition	0.63 mg/mL	NO and ET-1 inhibition	CHN	[168]
Iezolide (**135**)/ cyanobacterium	Polyketide ^e^	SERCA inhibition	10 nM *	Cell-cycle delay and apoptosis-signaling activation	JPN	[169]
Mandelalide A (**142**)/ ascidian	Terpenoid ^f^	AMPK activation	30 nM *	AMPKα subunit required for activation	FRA, USA	[170]
Marinobazzanan (**143**)/fungus	Terpenoid ^f^	Cell migration inhibition	2.5 µM *	KITENIN protein inhibition	S. KOR, USA	[171]
Manzamine A (**144**)/sponge	Alkaloid ^g^	Prevention of autophagosome degradation	4 µM *	AKT/mTOR pathway inhibition	CHN, USA	[172]
Membranoid G (**145**)/sponge	Terpenoid ^f^	DNA topoisomerase 1B inhibition	80 µM *	Irreversible DNA binding	ITA, USA	[173]
Okadaic acid (**146**)/dinoflagellates	Terpenoid ^f^	Inhibition of CYP enzymes	33 nM *	NF-κB and JAK/STAT signaling activation	DEU	[174]
Orsaldechlorin A & B (**147**, **148**)/fungus	Polyketide ^e^	Osteoclastogenesis inhibition	15 μM *	NF-kB pathway inhibition	CHN	[175]
Palytoxin (**149**)/coral	Polyketide ^e^	Apoptotic cell death induction	30 pM	Anti-apoptotic Mcl-1 protein expression inhibition	BEL, FRA; S. KOR, LUX, USA	[176]
Penerpene P (**150**)/fungus	Terpenoid ^f^	PTP1B inhibition	14.3 μM	Binding to active site by docking	CHN	[177]
Viridicatol (**151**)/fungus	Alkaloid ^g^	Osteoblast-stimulated bone formation	4.4 µM	AKT/GSK-3β signaling pathway activation	CHN	[178]
Penicitrinol B (**152**)/ fungus	Polyketide ^e^	Ferroptosis inhibition in vitro	2 µM	Lipid peroxidation and HO-1 expression reduction	CHN	[179]
Phloroglucinol (**77**)/alga	Polyketide ^e^	Apoptosis inhibition in vitro	10 µg/mL	Mitochondrial damage inhibition	S. KOR	[180]
Pretrichodermamide B (**153**)/fungus	Alkaloid ^g^	JAK/STAT3 pathway inhibition	1.19 µM	STAT3 binding and phosphorylation inhibition	CHN, USA	[181]
Phenochalasin A (**154**)/fungus	Alkaloid ^g^	Macrophage-derived lipid droplet formation inhibition	0.61 µM	G-actin binding	JPN	[182]
Saquayamycin B1 (**155**)/bacterium	Polyketide ^e^	Human CRC inhibition	0.2–0.8 µM	PI3K/AKT signaling pathway regulation	CHN	[183]
Sargaquinoic acid (**156**)/alga	Terpenoid ^f^	ACE inhibition	140 µM	ACE-active site docking completed	S. KOR	[184]
Sargahydroquinoic acid (**157**)/alga	Terpenoid ^f^	Basophil degranulation inhibition	0.05 µM	ERK, p38 and NF-kB pathway downregulation	S. KOR	[185]
*Sargassum carpophyllun* phlorotannin (**158**)/alga	Polyketide ^e^	PGD2 and TNF-α secretion inhibition in vitro	35.9 µM	Partial Iκb phosphorylation inhibition	JPN	[186]
*Sarcophyton glaucum* cembranoids (**159**, **160**)/soft coral	Terpenoid ^f^	INDO-induced peptic ulcer protection	6 mg/kg **	Enhanced mucin and antioxidant properties	SAU	[187]
*Stichopus japonicus* tetrapeptides (**161**,**162**)/sea cucumber	Peptide ^g^	Keratinocyte migration enhanced	1 µM *	AKT/ERK signaling activation	CHN	[188]
*Stylissa massa* alkaloid (**163**)/sponge	Alkaloid ^g^	Aldose reductase inhibition	13.1 µM	K_D_ towards ALR2 6.9 µM	CHN	[189]
11-*epi*-sinulariolide acetate (**164**)/soft coral	Terpenoid ^f^	In vitro apoptosis induction	10 μM *	Regulated by FOXO proteins	TWN	[190]
Steckwaic acid F (**165**)/fungus	Polyketide ^e^	Osteoclast differentiation inhibition	2 μM *	NF-kB pathway inhibition	CHN	[191]
Stellettin B (**166**)/sponge	Terpenoid ^f^	In vitro apoptosis induction	0.1 μM *	PI3K/Akt and mTOR signaling inhibition	TWN	[192]
Talaverrucin A (**167**)/ fungus	Polyketide ^e^	Zebrafish eyeless phenotype rescue	20 μM *	Wnt/β-catenin pathway inhibition	CHN	[193]
*Takifugu flavidus* peptide (**168**)/fish	Peptide ^g^	ACE inhibition	93.5 μM *	Competitive inhibition of ACE-binding site	CHN, USA	[194]
Thiaplakortone B (**169**)/sponge	Alkaloid ^g^	Osteoclastogenesis inhibition in vitro	2.5 μM	NFATc1 activation inhibition	AUS, CHN	[195]
Trichocarboline A (**170**)/fungus	Alkaloid ^g^	Collagen accumulation inhibition	10 μM *	Smad2/3 protein phosphorylation inhibition	CHN, USA	[196]
Trifuhalol A (**171**)/alga	Polyketide ^e^	Lipid accumulation inhibition	22 μM *	Adipogenic gene transcription inhibition	USA	[197]
Xyloallenoide derivative (**172**)/fungus	Alkaloid ^g^	Human EPC senescence prevention	1 μM *	Telomerase and SIRT1 expression increased	CHN	[198]
Xyloketal B (**173**)/fungus	Alkaloid ^g^	Hepatic steatosis inhibition	20, 40 mg/kg **	PPAR-α/PGC1α signaling activation	CHN	[199]

**^a^** **Organism**: *Kingdom Animalia*: ascidian, fish (Phylum Chordata), coral, hydroid (Phylum Cnidaria), sea cucumber, sea urchin (Phylum Echinodermata), oyster, octopus (Phylum Mollusca), sponge (Phylum Porifera); *Kingdom Fungi*: fungus; *Kingdom Plantae*: alga; *Kingdom Monera*: bacterium; cyanobacterium (Phylum Cyanobacteria). **^b^** **IC_50_**: concentration of a compound required for 50% inhibition in vitro; *: estimated IC_50_; ** in vivo study. **^c^** **MMOA**: molecular mechanism of action. **^d^** **Country/Region:** AUS: Australia; BEL: Belgium; CHN: China; DEU: Germany; ESP: Spain; FRA: France; GBR: United Kingdom; IND, India; ITA: Italy; JPN: Japan; LUX: Luxembourg; RUS: Russian Federation; SAU: Saudi Arabia; S. KOR: South Korea; THA: Thailand; TWN: Taiwan; USA: United States of America; VNM: Vietnam. **Chemistry:** **^e^** polyketide; **^f^** terpene; **^g^** nitrogen-containing compound; **^i^** fatty acid. **Abbreviations:** ACE: angiotensin-1-converting enzyme; Akt: protein kinase B; AMPK: AMP-activated protein kinase; CDK2: cyclin-dependent kinase-2; CRC: human colorectal cancer; CREB: cAMP response element-binding protein; eEF1A: GTPase elongation factor 1-alpha; EMT: epithelial–mesenchymal transition; EPC: endothelial progenitor cells; ERK: extracellular signal-regulated kinase; ET-1: endothelin-1; FOXO3: Forkhead box O3 transcription factor; GSK-3β: Glycogen synthase kinase-3 beta; HDF: human dermal fibroblasts; HO-1: heme oxygenase-1; HUVEC: human umbilical vein endothelial cells; IFN: interferon; INDO: indomethacin; JAK: Janus kinase; JNK: c-jun N-terminal kinase; Keap1: Kelch-like ECH-associated protein 1; MAPK: mitogen-activated protein kinase; MMP: matrix metalloproteinases; mTOR: mammalian target of rapamycin; NFATc1: nuclear factor of activated T cells 1; NF-κB: nuclear factor kappa-light-chain-enhancer of activated B cells; NO: nitric oxide; Nrf2: nuclear factor-erythroid 2-related factor 2; NSCLC: non-small-cell lung cancer; PAX3-FOXO1: fusion gene of PAX3 (a developmental transcription factor) and FOXO1 (a regulator of cell cycle and apoptosis); PGD2: prostaglandin D2; PI3K: Phosphoinositide 3-kinase; PPAR: peroxisome proliferator-activated receptor; PTP: protein tyrosine phosphatase; ROS: reactive oxygen species; SERCA: sarcoplasmic/endoplasmic reticulum Ca^2+^-ATPase; SIRT1: sirtuin 1; STAT: signal transducer and activator of transcription; TIR: taste receptor type 1 member; UV: ultraviolet; TBHP: Tert-Butyl Hydroperoxide; TG: triglyceride; TNF-α: tumor necrosis factor α; TYR: tyrosinase; TRP: tyrosinase-related protein; VDAC3: voltage-dependent anion-selective channel protein 3; UV: ultraviolet.

## Data Availability

Not applicable.

## References

[1] Mayer A.M.S., Lehmann V.K.B. (2000). Marine pharmacology in 1998: Marine compounds with antibacterial, anticoagulant, antifungal, anti-inflammatory, anthelmintic, antiplatelet, antiprotozoal, and antiviral activities; with actions on the cardiovascular, endocrine, immune, and nervous systems; and other miscellaneous mechanisms of action. Pharmacologist.

[2] Mayer A.M., Hamann M.T. (2002). Marine pharmacology in 1999: Compounds with antibacterial, anticoagulant, antifungal, anthelmintic, anti-inflammatory, antiplatelet, antiprotozoal and antiviral activities affecting the cardiovascular, endocrine, immune and nervous systems, and other miscellaneous mechanisms of action. Comp. Biochem. Physiol. C Toxicol. Pharmacol..

[3] Mayer A.M.S., Hamann M.T. (2004). Marine pharmacology in 2000: Marine compounds with antibacterial, anticoagulant, antifungal, anti-inflammatory, antimalarial, antiplatelet, antituberculosis, and antiviral activities; affecting the cardiovascular, immune, and nervous systems and other miscellaneous mechanisms of action. Mar. Biotechnol..

[4] Mayer A.M., Hamann M.T. (2005). Marine pharmacology in 2001–2002: Marine compounds with anthelmintic, antibacterial, anticoagulant, antidiabetic, antifungal, anti-inflammatory, antimalarial, antiplatelet, antiprotozoal, antituberculosis, and antiviral activities; affecting the cardiovascular, immune and nervous systems and other miscellaneous mechanisms of action. Comp. Biochem. Physiol. C Toxicol. Pharmacol..

[5] Mayer A.M., Rodriguez A.D., Berlinck R.G., Hamann M.T. (2007). Marine pharmacology in 2003–2004: Marine compounds with anthelmintic antibacterial, anticoagulant, antifungal, anti-inflammatory, antimalarial, antiplatelet, antiprotozoal, antituberculosis, and antiviral activities; affecting the cardiovascular, immune and nervous systems, and other miscellaneous mechanisms of action. Comp. Biochem. Physiol. C Toxicol. Pharmacol..

[6] Mayer A.M., Rodriguez A.D., Berlinck R.G., Hamann M.T. (2009). Marine pharmacology in 2005–2006: Marine compounds with anthelmintic, antibacterial, anticoagulant, antifungal, anti-inflammatory, antimalarial, antiprotozoal, antituberculosis, and antiviral activities; affecting the cardiovascular, immune and nervous systems, and other miscellaneous mechanisms of action. Biochim. Biophys. Acta.

[7] Mayer A.M., Rodriguez A.D., Berlinck R.G., Fusetani N. (2011). Marine pharmacology in 2007–2008: Marine compounds with antibacterial, anticoagulant, antifungal, anti-inflammatory, antimalarial, antiprotozoal, antituberculosis, and antiviral activities; affecting the immune and nervous system, and other miscellaneous mechanisms of action. Comp. Biochem. Physiol. C Toxicol. Pharmacol..

[8] Mayer A.M., Rodriguez A.D., Taglialatela-Scafati O., Fusetani N. (2013). Marine Pharmacology in 2009–2011: Marine Compounds with Antibacterial, Antidiabetic, Antifungal, Anti-Inflammatory, Antiprotozoal, Antituberculosis, and Antiviral Activities; Affecting the Immune and Nervous Systems, and other Miscellaneous Mechanisms of Action. Mar. Drugs.

[9] Mayer A.M.S., Rodriguez A.D., Taglialatela-Scafati O., Fusetani N. (2017). Marine Pharmacology in 2012–2013: Marine Compounds with Antibacterial, Antidiabetic, Antifungal, Anti-Inflammatory, Antiprotozoal, Antituberculosis, and Antiviral Activities; Affecting the Immune and Nervous Systems, and Other Miscellaneous Mechanisms of Action. Mar. Drugs.

[10] Mayer A.M.S., Guerrero A.J., Rodriguez A.D., Taglialatela-Scafati O., Nakamura F., Fusetani N. (2019). Marine Pharmacology in 2014–2015: Marine Compounds with Antibacterial, Antidiabetic, Antifungal, Anti-Inflammatory, Antiprotozoal, Antituberculosis, Antiviral, and Anthelmintic Activities; Affecting the Immune and Nervous Systems, and Other Miscellaneous Mechanisms of Action. Mar. Drugs.

[11] Mayer A.M.S., Guerrero A.J., Rodriguez A.D., Taglialatela-Scafati O., Nakamura F., Fusetani N. (2021). Marine Pharmacology in 2016–2017: Marine Compounds with Antibacterial, Antidiabetic, Antifungal, Anti-Inflammatory, Antiprotozoal, Antituberculosis and Antiviral Activities; Affecting the Immune and Nervous Systems, and Other Miscellaneous Mechanisms of Action. Mar. Drugs.

[12] Mayer A.M.S., Pierce M.L., Howe K., Rodríguez A.D., Taglialatela-Scafati O., Nakamura F., Fusetani N. (2022). Marine pharmacology in 2018: Marine compounds with antibacterial, antidiabetic, antifungal, anti-inflammatory, antiprotozoal, antituberculosis and antiviral activities; affecting the immune and nervous systems, and other miscellaneous mechanisms of action. Pharmacol. Res..

[13] Mayer A.M.S., Mayer V.A., Swanson-Mungerson M., Pierce M.L., Rodríguez A.D., Nakamura F., Taglialatela-Scafati O. (2024). Marine Pharmacology in 2019–2021: Marine Compounds with Antibacterial, Antidiabetic, Antifungal, Anti-Inflammatory, Antiprotozoal, Antituberculosis and Antiviral Activities; Affecting the Immune and Nervous Systems, and Other Miscellaneous Mechanisms of Action. Mar. Drugs.

[14] Schmitz F.J., Bowden B.F., Toth S.I. (1993). Antitumor and Cytotoxic Compounds from Marine Organisms. Pharmaceutical and Bioactive Natural Products.

[15] Zhuravleva O.I., Chingizova E.A., Oleinikova G.K., Starnovskaya S.S., Antonov A.S., Kirichuk N.N., Menshov A.S., Popov R.S., Kim N.Y., Berdyshev D.V. (2023). Anthraquinone Derivatives and Other Aromatic Compounds from Marine Fungus *Asteromyces cruciatus* KMM 4696 and Their Effects against *Staphylococcus aureus*. Mar. Drugs.

[16] Chen Y., Liu C., Kumaravel K., Nan L., Tian Y. (2022). Two New Sulfate-Modified Dibenzopyrones with Anti-foodborne Bacteria Activity From Sponge-Derived *Fungus Alternaria* sp. SCSIOS02F49. Front. Microbiol..

[17] Rončević T., Gerdol M., Mardirossian M., Maleš M., Cvjetan S., Benincasa M., Maravić A., Gajski G., Krce L., Aviani I. (2022). Anisaxins, helical antimicrobial peptides from marine parasites, kill resistant bacteria by lipid extraction and membrane disruption. Acta Biomater..

[18] Oluwabusola E.T., Katermeran N.P., Poh W.H., Goh T.M.B., Tan L.T., Diyaolu O., Tabudravu J., Ebel R., Rice S.A., Jaspars M. (2022). Inhibition of the Quorum Sensing System, Elastase Production and Biofilm Formation in *Pseudomonas aeruginosa* by Psammaplin A and Bisaprasin. Molecules.

[19] Safronova V.N., Panteleev P.V., Sukhanov S.V., Toropygin I.Y., Bolosov I.A., Ovchinnikova T.V. (2022). Mechanism of Action and Therapeutic Potential of the β-Hairpin Antimicrobial Peptide Capitellacin from the Marine Polychaeta *Capitella teleta*. Mar. Drugs.

[20] Tian J., Chen S., Liu F., Zhu Q., Shen J., Lin W., Zhu K. (2022). Equisetin Targets Intracellular *Staphylococcus aureus* through a Host Actng Strategy. Mar. Drugs.

[21] Huang B., Peng S., Liu S., Zhang Y., Wei Y., Xu X., Gao C., Liu Y., Luo X. (2022). Isolation, Screening, and Active Metabolites Identification of Anti-Vibrio Fungal Strains derived from the Beibu Gulf Coral. Front. Microbiol..

[22] Su J., Guan B., Su Q., Hu S., Wu S., Tong Z., Zhou F. (2023). Fucoxanthin Ameliorates Sepsis via Modulating Microbiota by Targeting IRF3 Activation. Int. J. Mol. Sci..

[23] Panteleev P.V., Safronova V.N., Duan S., Komlev A.S., Bolosov I.A., Kruglikov R.N., Kombarova T.I., Korobova O.V., Pereskokova E.S., Borzilov A.I. (2023). Novel BRICHOS-Related Antimicrobial Peptides from the Marine Worm *Heteromastus filiformis*: Transcriptome Mining, Synthesis, Biological Activities, and Therapeutic Potential. Mar. Drugs.

[24] Tran T.M.T., Addison R.S., Davis R.A., Rehm B.H.A. (2023). Bromotyrosine-Derived Metabolites from a Marine Sponge Inhibit *Pseudomonas aeruginosa* Biofilms. Int. J. Mol. Sci..

[25] Juskewitz E., Mishchenko E., Dubey V.K., Jenssen M., Jakubec M., Rainsford P., Isaksson J., Andersen J.H., Ericson J.U. (2022). Lulworthinone: In Vitro Mode of Action Investigation of an Antibacterial Dimeric Naphthopyrone Isolated from a Marine Fungus. Mar. Drugs.

[26] Khan N.A., Barthes N., McCormack G., O’Gara J.P., Thomas O.P., Boyd A. (2023). Sponge-derived fatty acids inhibit biofilm formation of MRSA and MSSA by down-regulating biofilm-related genes specific to each pathogen. J. Appl. Microbiol..

[27] Upender I., Yoshida O., Schrecengost A., Ranson H., Wu Q., Rowley D.C., Kishore S., Cywes C., Miller E.L., Whalen K.E. (2023). A marine-derived fatty acid targets the cell membrane of Gram-positive bacteria. J. Bacteriol..

[28] Zhao H., Ji R., Zha X., Xu Z., Lin Y., Zhou S. (2022). Investigation of the bactericidal mechanism of Penicilazaphilone C on *Escherichia coli* based on 4D label-free quantitative proteomic analysis. Eur. J. Pharm. Sci..

[29] Kim H.J., Lee M.S., Jeong S.K., Lee S.J. (2023). Transcriptomic analysis of the antimicrobial activity of prodigiosin against *Cutibacterium acnes*. Sci. Rep..

[30] Badran A.R. (2023). Isolation and structure elucidation of aromatic polyketides from marine actinomycete with antibiofilm activity against *Staphylococcus aureus* and *Escherichia coli*. Egypt. J. Chem..

[31] Guo S., Zhang Z., Xu X., Cai J., Wang W., Guo L. (2022). Antagonistic activity and mode of action of trypacidin from marine-derived *Aspergillus fumigatus* against *Vibrio parahaemolyticus*. 3 Biotech.

[32] Fu J., Luo X., Lin M., Xiao Z., Huang L., Wang J., Zhu Y., Liu Y., Tao H. (2023). Marine-Fungi-Derived Gliotoxin Promotes Autophagy to Suppress *Mycobacteria tuberculosis* Infection in Macrophages. Mar. Drugs.

[33] Chung B., Hwang J.Y., Park S.C., Kwon O.S., Cho E., Lee J., Lee H.S., Oh D.C., Shin J., Oh K.B. (2022). Inhibitory Effects of Nitrogenous Metabolites from a Marine-Derived *Streptomyces bacillaris* on Isocitrate Lyase of *Candida albicans*. Mar. Drugs.

[34] Zhu X., Wang A., Zheng Y., Li D., Wei Y., Gan M., Li Y., Si S. (2023). Anti-Biofilm Activity of Cocultimycin A against *Candida albicans*. Int. J. Mol. Sci..

[35] Kamauchi H., Furukawa M., Kiba Y., Kitamura M., Usui K., Katakura M., Takao K., Sugita Y. (2022). Antifungal activity of dehydrocurvularin for *Candida* spp. through the inhibition of adhesion to human adenocarcinoma cells. J. Antibiot..

[36] Kharat B.A., Said M.S., Dastager S.G. (2022). Antifungal compound from marine *Serratia marcescens* BKACT and its potential activity against *Fusarium* sp.. Int. Microbiol..

[37] Jiang M., Chen S., Lu X., Guo H., Yin X., Li H., Dai G., Liu L. (2023). Integrating Genomics and Metabolomics for the Targeted Discovery of New Cyclopeptides with Antifungal Activity from a Marine-Derived Fungus *Beauveria felina*. J. Agric. Food Chem..

[38] Elmaidomy A.H., Zahran E.M., Soltane R., Alasiri A., Saber H., Ngwa C.J., Pradel G., Alsenani F., Sayed A.M., Abdelmohsen U.R. (2022). New Halogenated Compounds from *Halimeda macroloba* Seaweed with Potential Inhibitory Activity against Malaria. Molecules.

[39] Arberas-Jiménez I., Nocchi N., Chao-Pellicer J., Sifaoui I., Soares A.R., Díaz-Marrero A.R., Fernández J.J., Piñero J.E., Lorenzo-Morales J. (2023). Chamigrane-Type Sesquiterpenes from *Laurencia dendroidea* as Lead Compounds against *Naegleria fowleri*. Mar. Drugs.

[40] Barbosa Da Silva E., Sharma V., Hernandez-Alvarez L., Tang A.H., Stoye A., O’Donoghue A.J., Gerwick W.H., Payne R.J., McKerrow J.H., Podust L.M. (2022). Intramolecular Interactions Enhance the Potency of Gallinamide A Analogues against *Trypanosoma cruzi*. J. Med. Chem..

[41] Nicolás-Hernández D.S., Rodríguez-Expósito R.L., López-Arencibia A., Bethencourt-Estrella C.J., Sifaoui I., Salazar-Villatoro L., Omaña-Molina M., Fernández J.J., Díaz-Marrero A.R., Piñero J.E. (2023). Meroterpenoids from *Gongolaria abies-marina* against Kinetoplastids: In Vitro activity and Programmed Cell Death Study. Pharmaceuticals.

[42] Arberas-Jiménez I., Rodríguez-Expósito R.L., San Nicolás-Hernández D., Chao-Pellicer J., Sifaoui I., Díaz-Marrero A.R., Fernández J.J., Piñero J.E., Lorenzo-Morales J. (2023). Marine Meroterpenoids Isolated from *Gongolaria abies-marina* Induce Programmed Cell Death in *Naegleria fowleri*. Pharmaceuticals.

[43] García-Davis S., López-Arencibia A., Bethencourt-Estrella C.J., San Nicolás-Hernández D., Viveros-Valdez E., Díaz-Marrero A.R., Fernández J.J., Lorenzo-Morales J., Piñero J.E. (2023). Laurequinone, a Lead Compound against *Leishmania*. Mar. Drugs.

[44] Wang M., Sciorillo A., Read S., Divsalar D.N., Gyampoh K., Zu G., Yuan Z., Mounzer K., Williams D.E., Montaner L.J. (2022). Ansellone J, a Potent in Vitro and ex Vivo HIV-1 Latency Reversal Agent Isolated from a *Phorbas* sp. Marine Sponge. J. Nat. Prod..

[45] ElNaggar M.H., Abdelwahab G.M., Kutkat O., GabAllah M., Ali M.A., El-Metwally M.E.A., Sayed A.M., Abdelmohsen U.R., Khalil A.T. (2022). Aurasperone A Inhibits SARS CoV-2 In Vitro: An Integrated In Vitro and In Silico Study. Mar. Drugs.

[46] Hu Y., Zhao K., Zhu J., Qi X., Chen Q., Song Y., Pang X., Liu Y., Wang J. (2023). New neuraminidase inhibitory alkaloids from the mangrove soil-derived fungus *Arthrinium* sp. SCSIO41305. J. Ocean Univ. China.

[47] Nagahawatta D.P., Liyanage N.M., Je J.G., Jayawardhana H.H.A.C., Jayawardena T.U., Jeong S.H., Kwon H.J., Choi C.S., Jeon Y.J. (2022). Polyphenolic Compounds Isolated from Marine Algae Attenuate the Replication of SARS-CoV-2 in the Host Cell through a Multi-Target Approach of 3CL^pro^ and PL^pro^. Mar. Drugs.

[48] Cao F., Liu X.M., Wang X., Zhang Y.H., Yang J., Li W., Luo D.Q., Liu Y.F. (2023). Structural diversity and biological activities of indole-diterpenoids from *Penicillium janthinellum* by co-culture with *Paecilomyces formosus*. Bioorg. Chem..

[49] Izumida M., Kotani O., Hayashi H., Smith C., Fukuda T., Suga K., Iwao M., Ishibashi F., Sato H., Kubo Y. (2022). Unique Mode of Antiviral Action of a Marine Alkaloid against Ebola Virus and SARS-CoV-2. Viruses.

[50] Sureram S., Arduino I., Ueoka R., Rittà M., Francese R., Srivibool R., Darshana D., Piel J., Ruchirawat S., Muratori L. (2022). The Peptide A-3302-B Isolated from a Marine Bacterium *Micromonospora* sp. Inhibits HSV-2 Infection by Preventing the Viral Egress from Host Cells. Int. J. Mol. Sci..

[51] Alhadrami H.A., Burgio G., Thissera B., Orfali R., Jiffri S.E., Yaseen M., Sayed A.M., Rateb M.E. (2022). Neoechinulin A as a Promising SARS-CoV-2 M^pro^ Inhibitor: In Vitro and In Silico Study Showing the Ability of Simulations in Discerning Active from Inactive Enzyme Inhibitors. Mar. Drugs.

[52] Williams D.E., Cassel J., Zhu J.L., Yang J.X., de Voogd N.J., Matainaho T., Salvino J.M., Wang Y.A., Montaner L.J., Tietjen I. (2023). Thorectidiol A Isolated from the Marine Sponge *Dactylospongia elegans* Disrupts Interactions of the SARS-CoV-2 Spike Receptor Binding Domain with the Host ACE2 Receptor. J. Nat. Prod..

[53] Avalon N.E., Nafie J., De Marco Verissimo C., Warrensford L.C., Dietrick S.G., Pittman A.R., Young R.M., Kearns F.L., Smalley T., Binning J.M. (2022). Tuaimenal A, a Meroterpene from the Irish Deep-Sea Soft Coral *Duva florida*, Displays Inhibition of the SARS-CoV-2 3CLpro Enzyme. J. Nat. Prod..

[54] Zhang J., Zhang B., Cai L., Liu L. (2022). New Dibenzo-α-pyrone Derivatives with α-Glucosidase Inhibitory Activities from the Marine-Derived Fungus *Alternaria alternata*. Mar. Drugs.

[55] Li D.D., Wang Y., Kim E., Hong J., Jung J.H. (2022). A New Fungal Triterpene from the Fungus *Aspergillus flavus* Stimulates Glucose Uptake without Fat Accumulation. Mar. Drugs.

[56] Casertano M., Genovese M., Santi A., Pranzini E., Balestri F., Piazza L., Del Corso A., Avunduk S., Imperatore C., Menna M. (2023). Evidence of Insulin-Sensitizing and Mimetic Activity of the Sesquiterpene Quinone Avarone, a Protein Tyrosine Phosphatase 1B and Aldose Reductase Dual Targeting Agent from the Marine Sponge *Dysidea avara*. Pharmaceutics.

[57] Wang W., Shi Y., Liu Y., Zhang Y., Wu J., Zhang G., Che Q., Zhu T., Li M., Li D. (2022). Brasilterpenes A-E, Bergamotane Sesquiterpenoid Derivatives with Hypoglycemic Activity from the Deep Sea-Derived Fungus *Paraconiothyrium brasiliense* HDN15-135. Mar. Drugs.

[58] Li D.D., Luo X., Ying W., La Kim E., Hong J., Lee J.H., Jung J.H. (2023). Peroxisome Proliferator Activated Receptor-γ Agonistic Compounds from the Jellyfish-Derived Fungus *Cladosporium oxysporum*. Chem. Biodivers..

[59] Cho C.H., Yoo G., Kim M., Kurniawati U.D., Choi I.W., Lee S.H. (2023). Dieckol, Derived from the Edible Brown Algae *Ecklonia cava*, Attenuates Methylglyoxal-Associated Diabetic Nephropathy by Suppressing AGE-RAGE Interaction. Antioxidants.

[60] Pham T.K., Nguyen T.H.T., Yun H.R., Vasileva E.A., Mishchenko N.P., Fedoreyev S.A., Stonik V.A., Vu T.T., Nguyen H.Q., Cho S.W. (2023). Echinochrome A Prevents Diabetic Nephropathy by Inhibiting the PKC-Iota Pathway and Enhancing Renal Mitochondrial Function in db/db Mice. Mar. Drugs.

[61] Li M., Li S., Hu J., Gao X., Wang Y., Liu Z., Zhang W. (2021). Thioester-Containing Benzoate Derivatives with α-Glucosidase Inhibitory Activity from the Deep-Sea-Derived Fungus *Talaromyces indigoticus* FS688. Mar. Drugs.

[62] Dat T. (2022). The study on biological activity and molecular docking of secondary metabolites from *Bacillus* sp. isolated from the mangrove plant *Rhizophora apiculata* Blume. Reg. Stud. Mar. Sci..

[63] Truong T.P.T., Tran T.M., Dai T.X.T., Tran C.L. (2023). Antihyperglycemic and anti-type 2 diabetic activity of marine hydroquinone isolated from brown algae (*Dictyopteris polypodioides*). J. Tradit. Complement. Med..

[64] Zhang Y., Shen J., Ma X., Yao M., Zhang Y., Cao D. (2022). Anti-inflammatory and antioxidant activities of acteoside isolated from *Acanthus ilicifolius* var. *Xiamenensis*. Appl. Biol. Chem..

[65] Vidal I., Castilla L., Marrero A.D., Bravo-Ruiz I., Bernal M., Manrique I., Quesada A.R., Medina M., Martínez-Poveda B. (2022). The Sponge-Derived Brominated Compound Aeroplysinin-1 Impairs the Endothelial Inflammatory Response through Inhibition of the NF-κB Pathway. Mar. Drugs.

[66] Liu G., Liu D., Li Z., Jiao J., Hou X., Zhang X., Che Q., Zhu T., Li D., Zhang G. (2022). Overexpression of transcriptional regulator and tailoring enzyme leads to the discovery of anti-inflammatory meroterpenoids from marine-derived fungus *Alternaria alternata* JJY-32. Front. Mar. Sci..

[67] Cao V.A., Kwon J.H., Kang J.S., Lee H.S., Heo C.S., Shin H.J. (2022). Aspersterols A-D, Ergostane-Type Sterols with an Unusual Unsaturated Side Chain from the Deep-Sea-Derived Fungus *Aspergillus unguis*. J. Nat. Prod..

[68] Sun L., Kim S., Mori R., Miyaji N., Nikawa T., Hirasaka K. (2022). Astaxanthin Exerts Immunomodulatory Effect by Regulating SDH-HIF-1α Axis and Reprogramming Mitochondrial Metabolism in LPS-Stimulated RAW264.7 Cells. Mar. Drugs.

[69] Liu Y., Li Y., Chen M., Liang J., Zhang Y., Qian Z.J. (2022). Mechanism of two alkaloids isolated from coral endophytic fungus for suppressing angiogenesis in atherosclerotic plaque in HUVEC. Int. Immunopharmacol..

[70] McCall J.R., Sausman K.T., Keeler D.M., Brown A.P., Turrise S.L. (2022). Immune Modulating Brevetoxins: Monocyte Cytotoxicity, Apoptosis, and Activation of M1/M2 Response Elements Is Dependent on Reactive Groups. Mar. Drugs.

[71] Ren X., Chen C., Ye Y., Xu Z., Zhao Q., Luo X., Liu Y., Guo P. (2022). Anti-inflammatory compounds from the mangrove endophytic fungus *Amorosia* sp. SCSIO 41026. Front. Microbiol..

[72] Al-Awadhi F.H., Simon E.F., Liu N., Ratnayake R., Paul V.J., Luesch H. (2023). Discovery and Anti-Inflammatory Activity of a Cyanobacterial Fatty Acid Targeting the Keap1/Nrf2 Pathway. Mar. Drugs.

[73] Gunasekera S.P., Kokkaliari S., Ratnayake R., Sauvage T., Dos Santos L.A.H., Luesch H., Paul V.J. (2022). Anti-Inflammatory Dysidazirine Carboxylic Acid from the Marine Cyanobacterium *Caldora* sp. Collected from the Reefs of Fort Lauderdale, Florida. Molecules.

[74] Jiao W.H., Li J.X., Liu H.Y., Luo X.C., Hu T.Y., Shi G.H., Xie D.D., Chen H.F., Cheng B.H., Lin H.W. (2023). Dysambiol, an Anti-inflammatory Secomeroterpenoid from a *Dysidea* sp. Marine Sponge. Org. Lett..

[75] Nagahawatta D.P., Liyanage N.M., Jayawardhana H.H.A.C., Jayawardena T.U., Lee H.G., Heo M.S., Jeon Y.J. (2022). Eckmaxol Isolated from *Ecklonia maxima* Attenuates Particulate-Matter-Induced Inflammation in MS-S Lung Macrophage. Mar. Drugs.

[76] Wang G., Yin Z., Wang S., Yuan Y., Chen Y., Kang W. (2022). Diversified Polyketides With Anti-inflammatory Activities From Mangrove Endophytic Fungus *Daldinia eschschholtzii* KBJYZ-1. Front. Microbiol..

[77] Ye J., Zheng J., Tian X., Xu B., Yuan F., Wang B., Yang Z., Huang F. (2022). Fucoxanthin Attenuates Free Fatty Acid-Induced Nonalcoholic Fatty Liver Disease by Regulating Lipid Metabolism/Oxidative Stress/Inflammation via the AMPK/Nrf2/TLR4 Signaling Pathway. Mar. Drugs.

[78] Ben Ammar R., Zahra H.A., Abu Zahra A.M., Alfwuaires M., Abdulaziz Alamer S., Metwally A.M., Althnaian T.A., Al-Ramadan S.Y. (2023). Protective Effect of Fucoxanthin on Zearalenone-Induced Hepatic Damage through Nrf2 Mediated by PI3K/AKT Signaling. Mar. Drugs.

[79] Kang H., Kim S.C., Oh Y. (2023). Fucoxanthin Abrogates Ionizing Radiation-Induced Inflammatory Responses by Modulating Sirtuin 1 in Macrophages. Mar. Drugs.

[80] Xiao Z., Yang S., Liu Y., Zhou C., Hong P., Sun S., Qian Z.J. (2022). A novel glyceroglycolipid from brown algae *Ishige okamurae* improve photoaging and counteract inflammation in UVB-induced HaCaT cells. Chem. Biol. Interact..

[81] Qin Y.Y., Li W.S., Zhang X., Yu Z.X., Li X.B., Chen H.Y., Lv Y.R., Han C.R., Chen G.Y. (2023). Meroterpenoids Possessing Diverse Rearranged Skeletons with Anti-Inflammatory Activity from the Mangrove-Derived Fungus *Penicillium* sp. HLLG -122. Chin. J. Chem..

[82] Cho W., Park J., Kim J., Lee M., Park S.J., Kim K.S., Jun W., Kim O.K., Lee J. (2023). Low-Molecular-Weight Fish Collagen Peptide (Valine-Glycine-Proline-Hydroxyproline-Glycine-Proline-Alanine-Glycine) Prevents Osteoarthritis Symptoms in Chondrocytes and Monoiodoacetate-Injected Rats. Mar. Drugs.

[83] Samarakoon K.W., Kuruppu A.I., Ko J.Y., Lee J.H., Jeon Y.J. (2023). Structural Characterization and Anti-Inflammatory Effects of 24-Methylcholesta-5(6), 22-Diene-3β-ol from the Cultured Marine Diatom *Phaeodactylum tricornutum* Attenuate Inflammatory Signaling Pathways. Mar. Drugs.

[84] Brancaccio M., Milito A., Viegas C.A., Palumbo A., Simes D.C., Castellano I. (2022). First evidence of dermo-protective activity of marine sulfur-containing histidine compounds. Free Radic. Biol. Med..

[85] Denisenko Y., Novgorodtseva T., Antonyuk M., Yurenko A., Gvozdenko T., Kasyanov S., Ermolenko E., Sultanov R. (2023). 1-O-alkyl-glycerols from Squid *Berryteuthis magister* Reduce Inflammation and Modify Fatty Acid and Plasmalogen Metabolism in Asthma Associated with Obesity. Mar. Drugs.

[86] Kirindage K., Jayasinghe A.M., Han E., Kim K., Wang L., Heo S., Jung K., Ahn G. (2023). Phlorofucofuroeckol-a Refined by Edible Brown Algae *Ecklonia Cava* Indicates Anti-Inflammatory Effects on Tnf-α/IFN-γ-Stimulated HaCat Keratinocytes and 12-O-Tetradecanoylphorbol 13-Acetate-Induced Ear Edema in Balb/C Mice. Funct. Foods.

[87] Marasinghe C.K., Jung W.K., Je J.Y. (2023). Phloroglucinol possesses anti-inflammatory activities by regulating AMPK/Nrf2/HO-1 signaling pathway in LPS-stimulated RAW264.7 murine macrophages. Immunopharmacol. Immunotoxicol..

[88] Wang G., Yuan Y., Li Z., Zhu J., She Z., Chen Y. (2023). Cytosporones with Anti-Inflammatory Activities from the Mangrove Endophytic Fungus *Phomopsis* sp. QYM-13. Mar. Drugs.

[89] Ohno O., Mizuno E., Miyamoto J., Hoshina T., Sano T., Matsuno K. (2022). Inhibition of Lipopolysaccharide-Induced Inflammatory Signaling by Soft Coral-Derived Prostaglandin A_2_ in RAW264.7 cells. Mar. Drugs.

[90] Ding W., Tian D., Chen M., Xia Z., Tang X., Zhang S., Wei J., Li X., Yao X., Wu B. (2023). Molecular Networking-Guided Isolation of Cyclopentapeptides from the Hydrothermal Vent Sediment Derived Fungus *Aspergillus pseudoviridinutans* TW58-5 and their Anti-inflammatory Effects. J. Nat. Prod..

[91] Nagahawatta D.P., Kim H.S., Jee Y.H., Jayawardena T.U., Ahn G., Namgung J., Yeo I.K., Sanjeewa K.K.A., Jeon Y.J. (2021). Sargachromenol Isolated from *Sargassum horneri* Inhibits Particulate Matter-Induced Inflammation in Macrophages through Toll-like Receptor-Mediated Cell Signaling Pathways. Mar. Drugs.

[92] Qi Y., Wang Z., Zhang J., Tang S., Zhu H., Jiang B., Li X., Wang J., Sun Z., Zhao M. (2023). Anti-Neuroinflammatory Meroterpenoids from a Chinese Collection of the Brown Alga *Sargassum siliquastrum*. J. Nat. Prod..

[93] Yang F., Sang M., Lu J.R., Zhao H.M., Zou Y., Wu W., Yu Y., Liu Y.W., Ma W., Zhang Y. (2023). Somalactams A-D: Anti-inflammatory Macrolide Lactams with Unique Ring Systems from an Arctic Actinomycete Strain. Angew. Chem. Int. Ed. Engl..

[94] Lee H.S., Nagahawatta D.P., Jeon Y.J., Lee M.A., Heo C.S., Park S.J., Shin H.J. (2023). Streptinone, a New Indanone Derivative from a Marine-Derived *Streptomyces massiliensis*, Inhibits Particulate Matter-Induced Inflammation. Mar. Drugs.

[95] Shin H.J., Heo C.S., Anh C.V., Yoon Y.D., Kang J.S. (2022). Streptoglycerides E-H, Unsaturated Polyketides from the Marine-Derived Bacterium *Streptomyces specialis* and Their Anti-Inflammatory Activity. Mar. Drugs.

[96] Anh C.V., Yoon Y.D., Kang J.S., Lee H.S., Heo C.S., Shin H.J. (2022). Nitrogen-Containing Secondary Metabolites from a Deep-Sea Fungus *Aspergillus unguis* and Their Anti-Inflammatory Activity. Mar. Drugs.

[97] Susana S.R., Salvador-Reyes L.A. (2022). Anti-Inflammatory Activity of Monosubstituted Xestoquinone Analogues from the Marine Sponge *Neopetrosia compacta*. Antioxidants.

[98] Um S., Lee J., Kim S.J., Cho K.A., Kang K.S., Kim S.H. (2023). Xinghamide A, a New Cyclic Nonapeptide Found in *Streptomyces xinghaiensis*. Mar. Drugs.

[99] Kudryashova E.K., Makarieva T.N., Shubina L.K., Guzii A.G., Popov R.S., Menshov A.S., Berdyshev D.V., Pislyagin E.A., Menchinskaya E.S., Grebnev B.B. (2023). Assimiloside A, a Glycolipid with Immunomodulatory Activity from the Northwestern Pacific Marine Sponge *Hymeniacidon assimilis*. J. Nat. Prod..

[100] Zhao R., Jiang X.X., Zhao Q.L., Ye H.W., Lin Y., Huang J., Tang Y.P. (2022). Immunoenhancing Effects of *Cyclina sinensis* Pentadecapeptide through Modulation of Signaling Pathways in Mice with Cyclophosphamide-induced Immunosuppression. Mar. Drugs.

[101] Kim E.A., Han E.J., Kim J., Fernando I.P.S., Oh J.Y., Kim K.N., Ahn G., Heo S.J. (2022). Anti-Allergic Effect of 3,4-Dihydroxybenzaldehyde Isolated from *Polysiphonia morrowii* in IgE/BSA-stimulated Mast Cells and a Passive Cutaneous Anaphylaxis Mouse Model. Mar. Drugs.

[102] Abdelmawgood I.A., Mahana N.A., Badr A.M., Mohamed A.S., Al Shawoush A.M., Atia T., Abdelrazak A.E., Sakr H.I. (2023). Echinochrome Ameliorates Physiological, Immunological, and Histopathological Alterations Induced by Ovalbumin in Asthmatic Mice by Modulating the Keap1/Nrf2 Signaling Pathway. Mar. Drugs.

[103] Liao M., Wei S., Hu X., Liu J., Wang J. (2023). Protective Effect and Mechanisms of Eckol on Chronic Ulcerative Colitis Induced by Dextran Sulfate Sodium in Mice. Mar. Drugs.

[104] Miao S., He Q., Li C., Wu Y., Liu M., Chen Y., Qi S., Gong K. (2022). Aaptamine—A dual acetyl—And butyrylcholinesterase inhibitor as potential anti-Alzheimer’s disease agent. Pharm. Biol..

[105] Sung C.S., Cheng H.J., Chen N.F., Tang S.H., Kuo H.M., Sung P.J., Chen W.F., Wen Z.H. (2023). Antinociceptive Effects of Aaptamine, a Sponge Component, on Peripheral Neuropathy in Rats. Mar. Drugs.

[106] An D., Pinheiro-Junior E.L., Béress L., Gladkikh I., Leychenko E., Undheim E.A.B., Peigneur S., Tytgat J. (2022). AsKC11, a Kunitz Peptide from *Anemonia sulcata*, Is a Novel Activator of G Protein-Coupled Inward-Rectifier Potassium Channels. Mar. Drugs.

[107] Jiang M., Guo H., Wu Q., Yuan S., Liu L. (2022). Two New Picoline-Derived Meroterpenoids with Anti-Acetylcholinesterase Activity from Ascidian-Derived Fungus *Amphichorda felina*. Molecules.

[108] Mehrotra S., Pierce M.L., Cao Z., Jabba S.V., Gerwick W.H., Murray T.F. (2022). Antillatoxin-Stimulated Neurite Outgrowth Involves the Brain-Derived Neurotrophic Factor (BDNF)—Tropomyosin Related Kinase B (TrkB) Signaling Pathway. J. Nat. Prod..

[109] Xiao X., Tong Z., Zhang Y., Zhou H., Luo M., Hu T., Hu P., Kong L., Liu Z., Yu C. (2022). Novel Prenylated Indole Alkaloids with Neuroprotection on SH-SY5Y Cells against Oxidative Stress Targeting Keap1-Nrf2. Mar. Drugs.

[110] Park J.H., Lee T.K., Kim D.W., Ahn J.H., Lee C.H., Kim J.D., Shin M.C., Cho J.H., Lee J.C., Won M.H. (2022). Astaxanthin Confers a Significant Attenuation of Hippocampal Neuronal Loss Induced by Severe Ischemia-Reperfusion Injury in Gerbils by Reducing Oxidative Stress. Mar. Drugs.

[111] Wang X., Zhang T., Chen X., Xu Y., Li Z., Yang Y., Du X., Jiang Z., Ni H. (2022). Simultaneous Inhibitory Effects of All-Trans Astaxanthin on Acetylcholinesterase and Oxidative Stress. Mar. Drugs.

[112] Paramakrishnan N., Lim K.G., Paramaswaran Y., Ali N., Waseem M., Shazly G.A., Bin Jardan Y.A., Muthuraman A. (2023). Astaxanthin: A Marine Drug That Ameliorates Cerebrovascular-Damage-Associated Alzheimer’s Disease in a Zebrafish Model via the Inhibition of Matrix Metalloprotease-13. Mar. Drugs.

[113] Fujihara K., Hashimoto T., Sasaki H., Koyama K., Kinoshita K. (2023). Inhibition of Aβ aggregation by naphtho-γ-pyrone derivatives from a marine-derived fungus, *Aspergillus* sp. MPUC239. J. Nat. Med..

[114] Nie Y., Yang J., Zhou L., Yang Z., Liang J., Liu Y., Ma X., Qian Z., Hong P., Kalueff A.V. (2022). Marine fungal metabolite butyrolactone I prevents cognitive deficits by relieving inflammation and intestinal microbiota imbalance on aluminum trichloride-injured zebrafish. J. Neuroinflamm..

[115] Mehrotra S., Pierce M.L., Dravid S.M., Murray T.F. (2022). Stimulation of Neurite Outgrowth in Cerebrocortical Neurons by Sodium Channel Activator Brevetoxin-2 Requires Both N-Methyl-D-aspartate Receptor 2B (GluN2B) and p21 Protein (Cdc42/Rac)-Activated Kinase 1 (PAK1). Mar. Drugs.

[116] Wang L., Wu X., Zhu X., Zhangsun D., Wu Y., Luo S. (2022). A Novel α4/7-Conotoxin QuIA Selectively Inhibits α3β2 and α6/α3β4 Nicotinic Acetylcholine Receptor Subtypes with High Efficacy. Mar. Drugs.

[117] Wang H. (2022). Synthesis and characterization of αM-conotoxin SIIID, a reversible human α7 nicotinic acetylcholine receptor antagonist. Toxicon.

[118] Zhang J., Liu D., Fan A., Huang J., Lin W. (2022). Eremophilane-Type Sesquiterpenes from a Marine-Derived Fungus *Penicillium Copticola* with Antitumor and Neuroprotective Activities. Mar. Drugs.

[119] Qi Y., Liu G., Fang C., Jing C., Tang S., Li G., Wang C., Zhu H., Zhao M., Sun Z. (2023). Antioxidant and Neuroprotective Xenicane Diterpenes from the Brown Alga *Dictyota coriacea*. ACS Omega.

[120] Sanguanphun T., Sornkaew N., Malaiwong N., Chalorak P., Jattujan P., Niamnont N., Sobhon P., Meemon K. (2022). Neuroprotective effects of a medium chain fatty acid, decanoic acid, isolated from *H. leucospilota* against Parkinsonism in *C. elegans* PD model. Front. Pharmacol..

[121] Wang X.X., Chen Z.L., Zhang J.S., Liu H.S., Ma R.P., Liu X.P., Li M.Y., Ge D., Bao J., Zhang H. (2023). Indole Diterpenes from Mangrove Sediment-Derived Fungus *Penicillium* sp. UJNMF9740 Protect PC12 cells againt 6-OHDA-Induced neurotoxicity via Regulating the PI3K/Akt Pathway. Mar. Drugs.

[122] Kim S.E., Chung E.D.S., Vasileva E.A., Mishchenko N.P., Fedoreyev S.A., Stonik V.A., Kim H.K., Nam J.H., Kim S.J. (2023). Multiple Effects of Echinochrome A on Selected Ion Channels Implicated in Skin Physiology. Mar. Drugs.

[123] Zhao Y., Lu Z., Xu X., Sun N., Lin S. (2022). Sea Cucumber-Derived Peptide Attenuates Scopolamine-Induced Cognitive Impairment by Preventing Hippocampal Cholinergic Dysfunction and Neuronal Cell Death. J. Agric. Food Chem..

[124] Liu J., Lu Y., Tang M., Shao F., Yang D., Chen S., Xu Z., Zhai L., Chen J., Li Q. (2022). Fucoxanthin Prevents Long-Term Administration l-DOPA-Induced Neurotoxicity through the ERK/JNK-c-Jun System in 6-OHDA-Lesioned Mice and PC12 Cells. Mar. Drugs.

[125] Hong D.D., Thom L.T., Ha N.C., Thu N.T.H., Hien H.T.M., Tam L.T., Dat N.M., Duc T.M., Tru N.V., Hang N.T.M. (2023). Isolation of Fucoxanthin from *Sargassum oligocystum* Montagne, 1845 Seaweed in Vietnam and Its Neuroprotective Activity. Biomedicines.

[126] Gladkikh I.N., Klimovich A.A., Kalina R.S., Kozhevnikova Y.V., Khasanov T.A., Osmakov D.I., Koshelev S.G., Monastyrnaya M.M., Andreev Y.A., Leychenko E.V. (2023). Anxiolytic, Analgesic and Anti-Inflammatory Effects of Peptides Hmg 1b-2 and Hmg 1b-4 from the Sea Anemone *Heteractis magnifica*. Toxins.

[127] Kvetkina A., Pislyagin E., Menchinskaya E., Yurchenko E., Kalina R., Kozlovskiy S., Kaluzhskiy L., Menshov A., Kim N., Peigneur S. (2022). Kunitz-Type Peptides from Sea Anemones Protect Neuronal Cells against Parkinson’s Disease Inductors via Inhibition of ROS Production and ATP-Induced P2X7 Receptor Activation. Int. J. Mol. Sci..

[128] Logashina Y.A., Lubova K.I., Maleeva E.E., Palikov V.A., Palikova Y.A., Dyachenko I.A., Andreev Y.A. (2022). Analysis of Structural Determinants of Peptide MS 9a-1 Essential for Potentiating of TRPA1 Channel. Mar. Drugs.

[129] Sanguanphun T., Promtang S., Sornkaew N., Niamnont N., Sobhon P., Meemon K. (2023). Anti-Parkinson Effects of *Holothuria leucospilota*-Derived Palmitic Acid in *Caenorhabditis elegans* Model of Parkinson’s Disease. Mar. Drugs.

[130] Park S.-R., Kim Y.H., Yang S.Y. (2023). Enzyme kinetics and molecular docking investigation of acetylcholinesterase and butyrylcholinesterase inhibitors from the marine alga *Ecklonia cava*. Nat. Prod. Product. Sci..

[131] Han E.J., Zhang C., Kim H.S., Kim J.Y., Park S.M., Jung W.K., Ahn G., Cha S.H. (2022). Sargachromenol Isolated from *Sargassum horneri* Attenuates Glutamate-Induced Neuronal Cell Death and Oxidative Stress through inhibition of MAPK/NF-κB and Activation of Nrf2/HO-1 Signaling Pathway. Mar. Drugs.

[132] Wang H.L., Li R., Zhao M., Wang Z.Y., Tang H., Cao Z.Y., Zheng G.L., Zhang W. (2023). A Drimane Meroterpenoid Borate as a Synchronous Ca+ Oscillation Inhibitor from the Coral-Associated Fungus *Alternaria* sp. ZH-15. J. Nat. Prod..

[133] He Q.Q., Man Y.Q., Sun K.L., Yang L.J., Wu Y., Du J., Chen W.W., Dai J.J., Ni N., Miao S. (2022). Aaptamine derivatives with CDK2 inhibitory activities from the South China Sea sponge *Aaptos suberitioides*. Nat. Prod. Res..

[134] Feng L., Lu C.K., Wu J., Chan L.L., Yue J. (2023). Identification of Anhydrodebromoaplysiatoxin as a Dichotomic Autophagy Inhibitor. Mar. Drugs.

[135] Song N., Tang Y., Wang Y., Guan X., Yu W., Jiang T., Lu L., Gu Y. (2023). A SIRT6 Inhibitor, Marine-Derived Pyrrole-Pyridinimidazole Derivative 8a, Suppresses Angiogenesis. Mar. Drugs.

[136] Lu I.T., Lin S.C., Chu Y.C., Wen Y., Lin Y.C., Cheng W.C., Sheu J.H., Lin C.C. (2022). (-)-Agelasidine A Induces Endoplasmic Reticulum Stress-Dependent Apoptosis in Human Hepatocellular Carcinoma. Mar. Drugs.

[137] Hoang T., Luu T.T., Ngo T.H.T., Nguyen T.M.H., Tran H.G., Nguyen T.T.O., Chau V.M., Hong D.D. (2022). Hypolipidaemic effects of (24R)-4α-methyl-5α-stigmasta-7,22-dien-3β-ol derived from *Aurantiochytrium mangrovei* BT3 in the HEPG2 cell line. Appl. Biochem. Microbiol..

[138] Chen M., Luo J., Ji H., Song W., Zhang D., Su W., Liu S. (2023). The Preventive Mechanism of Anserine on Tert-Butyl Hydroperoxide-Induced Liver Injury in L-02 Cells via Regulating the Keap1-Nrf2 and JNK-Caspase-3 Signaling Pathways. Mar. Drugs.

[139] Kitao M., Yamaguchi A., Tomioka T., Kai K., Kamei Y., Sugimoto K., Akagawa M. (2023). Astaxanthin protects human ARPE-19 retinal pigment epithelium cells from blue light-induced phototoxicity by scavenging singlet oxygen. Free Radic. Res..

[140] Peng Z., Gao J., Su W., Cao W., Zhu G., Qin X., Zhang C., Qi Y. (2022). Purification and Identification of Peptides from Oyster *Crassostrea hongkongensis* Protein Enzymatic Hydrolysates and Their Anti-Skin Photoaging Effects on UVB-Irradiated HaCaT Cells. Mar. Drugs.

[141] Wang X., Cong P., Wu L., Ma Y., Wang Z., Jiang T., Xu J. (2022). Sea cucumber ether-phospholipids improve hepatic steatosis and enhance hypothalamic autophagy in high-fat diet-fed mice. J. Nutr. Biochem..

[142] Cui Y., Zhang M., Xu H., Zhang T., Zhang S., Zhao X., Jiang P., Li J., Ye B., Sun Y. (2022). Elastase Inhibitor Cyclotheonellazole A: Total Synthesis and In Vivo Biological Evaluation for Acute Lung Injury. J. Med. Chem..

[143] Vidal I., Torres-Vargas J.A., Sánchez J.M., Trigal M., García-Caballero M., Medina M., Quesada A.R. (2023). Danthron, an Anthraquinone Isolated from a Marine Fungus, Is a New Inhibitor of Angiogenesis Exhibiting Interesting Antitumor and Antioxidant Properties. Antioxidants.

[144] Jiang W., Tian X., Wang D., Bokesch H.R., Thomas C.L., Woldemichael G.M., Gryder B.E., Wei J.S., Song Y.K., Chou H.C. (2022). Dentithecamides A-H, Diacylated Zoanthoxanthin Derivatives with PAX3-FOXO1 Inhibitory Activity from the Hydroid *Dentitheca haberi*. J. Nat. Prod..

[145] Lee S.E., Kim M.J., Hillman P.F., Oh D.C., Fenical W., Nam S.J., Lim K.M. (2022). Deoxyvasicinone with Anti-Melanogenic Activity from Marine-Derived *Streptomyces* sp. CNQ-617. Mar. Drugs.

[146] Juette M.F., Carelli J.D., Rundlet E.J., Brown A., Shao S., Ferguson A., Wasserman M.R., Holm M., Taunton J., Blanchard S.C. (2022). Didemnin B and ternatin-4 differentially inhibit conformational changes in eEF1A required for aminoacyl-tRNA accommodation into mammalian ribosomes. eLife.

[147] Liu H., Xie H., Li C., Wang L., Chen Q., Ouyang X., Yan C. (2022). Diaporisoindole B Reduces Lipid Accumulation in THP-1 Macrophage Cells via MAPKs and PPARγ-LXRα Pathways and Promotes the Reverse Cholesterol Transport by Upregulating SR-B1 and LDLR in HepG2 Cells. J. Nat. Prod..

[148] Kim J.M., Chung K.S., Yoon Y.S., Jang S.Y., Heo S.W., Park G., Jang Y.P., Ahn H.S., Shin Y.K., Lee S.H. (2022). Dieckol Isolated from *Eisenia bicyclis* Ameliorates Wrinkling and Improves Skin Hydration via MAPK/AP-1 and TGF-β/Smad Signaling Pathways in UVB-Irradiated Hairless Mice. Mar. Drugs.

[149] Liu J., Gao S., Zhou W., Chen Y., Wang Z., Zeng Z., Zhou H., Lin T. (2023). Dihydrotrichodimerol Purified from the Marine Fungus *Acremonium citrinum* NAFLD by Targeting PPARα. J. Nat. Prod..

[150] Ahn J.S., Shin Y.Y., Oh S.J., Song M.H., Kang M.J., Park S.Y., Nguyen P.T., Nguyen D.K., Kim H.K., Han J. (2022). Implication of Echinochrome A in the Plasticity and Damage of Intestinal Epithelium. Mar. Drugs.

[151] Choi M.R., Lee H., Kim H.K., Han J., Seol J.E., Vasileva E.A., Mishchenko N.P., Fedoreyev S.A., Stonik V.A., Ju W.S. (2022). Echinochrome A Inhibits Melanogenesis in B16F10 Cells by Downregulating CREB Signaling. Mar. Drugs.

[152] Feng J., Li S., Zhang B., Duan N., Zhou R., Yan S., Elango J., Liu N., Wu W. (2022). FGFC1 Exhibits Anti-Cancer Activity via Inhibiting NF-κB Signaling Pathway in *EGFR*-Mutant NSCLC cells. Mar. Drugs.

[153] Gao C., Tang S., Zhang H., Zhang T., Bao B., Zhu Y., Wu W. (2022). A Novel Marine Pyran-Isoindolone Compound Enhances Fibrin Lysis Mediated by Single-Chain Urokinase-Type Plasminogen Activator. Mar. Drugs.

[154] Kirindage K.G.I.S., Jayasinghe A.M.K., Han E.J., Jee Y., Kim H.J., Do S.G., Fernando I.P.S., Ahn G. (2022). Fucosterol Isolated from Dietary Brown Alga Sargassum horneri Protects TNF-α/IFN-γ-Stimulated Human Dermal Fibroblasts Via Regulating Nrf2/Ho-1 and NF-κB/MAPK Pathways. Antioxidants.

[155] Cui S., Wu H., He Q., Wang L., Yi X., Feng G., Wu Q., Tao B., Han D., Hu Q. (2023). Fucoxanthin alleviated atherosclerosis by regulating PI3K/AKT and TLR4/NFκB mediated pyroptosis in endothelial cells. Int. Immunopharmacol..

[156] Fang X., Zhu Y., Zhang T., Li Q., Fan L., Li X., Jiang D., Lin J., Zou L., Ren J. (2022). Fucoxanthin Inactivates the PI3K/Akt Signaling Pathway to Mediate Malignant Biological Behaviors of Non-Small Cell Lung Cancer. Nutr. Cancer.

[157] Zhang Y., Li Z., Huang B., Liu K., Peng S., Liu X., Gao C., Liu Y., Tan Y., Luo X. (2022). Anti-Osteoclastogenic and Antibacterial Effects of Chlorinated Polyketides from the Beibu Gulf Coral-Derived Fungus *Aspergillus unguis* GXIMD 02505. Mar. Drugs.

[158] Ueda G., Matsuo Y., Murase H., Aoyama Y., Kato T., Omi K., Hayashi Y., Imafuji H., Saito K., Tsuboi K. (2022). 10Z-Hymenialdisine inhibits angiogenesis by suppressing NF-κB activation in pancreatic cancer cell lines. Oncol. Rep..

[159] Janta S., Pranweerapaiboon K., Vivithanaporn P., Plubrukarn A., Chairoungdua A., Prasertsuksri P., Apisawetakan S., Chaithirayanon K. (2023). Holothurin A Inhibits RUNX1-Enhanced EMT in Metastasis Prostate Cancer via the Akt/JNK and P38 MAPK Signaling Pathway. Mar. Drugs.

[160] Kokkaliari S., Luo D., Paul V.J., Luesch H. (2023). Isolation and Biological Activity of Iezoside and Iezoside B, SERCA Inhibitors from Floridian Marine Cyanobacteria. Mar. Drugs.

[161] Kunnappilly Paulose S., Chakraborty K. (2022). Oxaspiro Indiculides from Old Woman *Octopus Cistopus indicus* as Dual Inhibitors of Inducible Cyclooxygenase and Lipoxygenase. Chem. Biodivers..

[162] Pan N., Li Z.C., Li Z.H., Chen S.H., Jiang M.H., Yang H.Y., Liu Y.S., Hu R., Zeng Y.W., Dai L.H. (2021). Antiplatelet and Antithrombotic Effects of Isaridin E Isolated from the Marine-Derived Fungus via Downregulating the PI3K/Akt Signaling Pathway. Mar. Drugs.

[163] Akishino M., Aoki Y., Baba H., Asakawa M., Hama Y., Mitsutake S. (2022). Red algae-derived isofloridoside activates the sweet taste receptor T1R2/T1R3. Food Biosci..

[164] Cho S.H., Kim H.S., Jung H.Y., Park J.I., Jang Y.J., Ahn J., Kim K.N. (2023). Effect of Ishophloroglucin A Isolated from *Ishige okamurae* on In Vitro Osteoclastogenesis and Osteoblastogenesis. Mar. Drugs.

[165] Im S.T., Kim H.S., Jung W.K., Lee S.H. (2022). Ishophloroglucin A, a potent PTP1B inhibitor isolated from brown alga *Ishige okamurae* inhibits adipogenesis in 3T3-L1 adipocytes. Fitoterapia.

[166] Tan Y., Ke M., Li Z., Chen Y., Zheng J., Wang Y., Zhou X., Huang G., Li X. (2021). A Nitrobenzoyl Sesquiterpenoid Insulicolide A Prevents Osteoclast Formation via Suppressing c-Fos-NFATc1 Signaling Pathway. Front. Pharmacol..

[167] Luo D., Ratnayake R., Atanasova K.R., Paul V.J., Luesch H. (2023). Targeted and functional genomics approaches to the mechanism of action of lagunamide D, a mitochondrial cytotoxin from marine cyanobacteria. Biochem. Pharmacol..

[168] Hu Y.D., Xi Q.H., Kong J., Zhao Y.Q., Chi C.F., Wang B. (2023). Angiotensin-I-Converting Enzyme (ACE)-Inhibitory Peptides from the Collagens of Monkfish *Lophius litulon* Swim Bladders: Isolation, Characterization, Molecular Docking, Analysis and Activity Evaluation. Mar. Drugs.

[169] Kurisawa N., Iwasaki A., Teranuma K., Dan S., Toyoshima C., Hashimoto M., Suenaga K. (2022). Structural Determination, Total Synthesis, and Biological Activity of Iezoside, a Highly Potent Ca^2+^-ATPase Inhibitor from the Marine Cyanobacterium *Leptochromothrix valpuliae*. J. Am. Chem. Soc..

[170] Mattos D.R., Wan X., Serrill J.D., Nguyen M.H., Humphreys I.R., Viollet B., Smith A.B., McPhail K.L., Ishmael J.E. (2022). The Marine-Derived Macrolactone Mandelalide A Is an Indirect Activator of AMPK. Mar. Drugs.

[171] Pulat S., Hillman P.F., Kim S., Asolkar R.N., Kim H., Zhou R., Taş İ., Gamage C.D.B., Varlı M., Park S.Y. (2023). Marinobazzanan, a Bazzanane-Type Sesquiterpenoid, Suppresses the Cell Motility and Tumorigenesis in Cancer Cells. Mar. Drugs.

[172] Wang X., Liu Y., Qin H., Qi G., Chen X., Lyu Y., Han Y. (2023). RIP1 Mediates Manzamine-A-Induced Secretory Autophagy in Breast Cancer. Mar. Drugs.

[173] Ottaviani A., Welsch J., Agama K., Pommier Y., Desideri A., Baker B.J., Fiorani P. (2022). From Antarctica to cancer research: A novel human DNA topoisomerase 1B inhibitor from Antarctic sponge *Dendrilla antarctica*. J. Enzym. Inhib. Med. Chem..

[174] Wuerger L.T.D., Kudiabor F., Alarcan J., Templin M., Poetz O., Sieg H., Braeuning A. (2023). Okadaic Acid Activates JAK/STAT Signaling to Affect Xenobiotic Metabolism in HepaRG Cells. Cells.

[175] Lu H., Tan Y., Zhang Y., Li Z., Chen J., Gao C., Liu Y., Luo X. (2022). Osteoclastogenesis inhibitory phenolic derivatives produced by the Beibu Gulf coral-associated fungus *Acremonium sclerotigenum* GXIMD 02501. Fitoterapia.

[176] Kim J., Ji S., Lee J.Y., Lorquin J., Orlikova-Boyer B., Cerella C., Mazumder A., Muller F., Dicato M., Detournay O. (2023). Marine Polyether Phycotoxin Palytoxin Induces Apoptotic Cell Death via Mcl-1 and Bcl-2 Downregulation. Mar. Drugs.

[177] Zhang F., Yang L., Xie Q.Y., Guo J.C., Ma Q.Y., Dai L.T., Zhou L.M., Dai H.F., Kong F.D., Luo D.Q. (2023). Diverse indole-diterpenoids with protein tyrosine phosphatase 1B inhibitory activities from the marine coral-derived fungus *Aspergillus* sp. ZF-104. Phytochemistry.

[178] Xie C.L., Yue Y.T., Xu J.P., Li N., Lin T., Ji G.R., Yang X.W., Xu R. (2023). Penicopeptide A (PPA) from the deep-sea-derived fungus promotes osteoblast-mediated bone formation and alleviates ovariectomy-induced bone loss by activating the AKT/GSK-3β/β-catenin signaling pathway. Pharmacol. Res..

[179] Yu H.Y., Li Y., Zhang M., Zou Z.B., Hao Y.J., Xie M.M., Li L.S., Meng D.L., Yang X.W. (2023). Chemical Constituents of the Deep-sea Gammarid Shrimp-Derived Fungus *Penicillium citrinum* XIA-16. Chem. Biodivers..

[180] Park C., Cha H.J., Hwangbo H., Ji S.Y., Kim D.H., Kim M.Y., Bang E., Hong S.H., Kim S.O., Jeong S.J. (2023). Phloroglucinol Inhibits Oxidative-Stress-Induced Cytotoxicity in C2C12 Murine Myoblasts through Nrf-2-Mediated Activation of HO-1. Int. J. Mol. Sci..

[181] Li R., Zhou Y., Zhang X., Yang L., Liu J., Wightman S.M., Lv L., Liu Z., Wang C.Y., Zhao C. (2023). Identification of marine natural product Pretrichodermamide B as a STAT3 inhibitor for efficient anticancer therapy. Mar. Life Sci. Technol..

[182] Kobayashi K., Matsuda D., Tomoda H., Ohshiro T. (2022). Binding of phenochalasin A, an inhibitor of lipid droplet formation in mouse macrophages, on G-actin. Drug Discov. Ther..

[183] Li J., Han N., Zhang H., Xie X., Zhu Y., Zhang E., Ma J., Shang C., Yin M., Xie W. (2022). Saquayamycin B_1_ Suppresses Proliferation, Invasion, and Migration by Inhibiting PI3K/AKT Signaling Pathway in Human Colorectal Cancer Cells. Mar. Drugs.

[184] Ko S.C., Kim J.Y., Lee J.M., Yim M.J., Kim H.S., Oh G.W., Kim C.H., Kang N., Heo S.J., Baek K. (2023). Angiotensin I-Converting Enzyme (ACE) Inhibition and Molecular Docking Study of Meroterpenoids Isolated from Brown Alga, *Sargassum macrocarpum*. Int. J. Mol. Sci..

[185] Seong Choi K., Shin T.S., Chun J., Ahn G., Jeong Han E., Kim M.J., Kim J.B., Kim S.H., Kho K.H., Heon Kim D. (2022). Sargahydroquinoic acid isolated from *Sargassum serratifolium* as inhibitor of cellular basophils activation and passive cutaneous anaphylaxis in mice. Int. Immunopharmacol..

[186] Matsui T., Ito C., Itoigawa M., Shibata T. (2022). Three phlorotannins from *Sargassum carpophyllum* are effective against the secretion of allergic mediators from antigen-stimulated rat basophilic leukemia cells. Food Chem..

[187] Bawakid N.O., Alorfi H.S., Alqarni N.M., Abdel-Naim A.B., Alarif W.M. (2023). Cembranoids from the Red Sea soft coral *Sarcophyton glaucum* protect against indomethacin-induced gastric injury. Naunyn Schmiedebergs Arch. Pharmacol..

[188] Zheng Z., Sun N., Lu Z., Zheng J., Zhang S., Lin S. (2023). The potential mechanisms of skin wound healing mediated by tetrapeptides from sea cucumber. Food Biosci..

[189] Wang Q., Gao C., Wei Z., Tang X., Ji L., Luo X., Peng X., Li G., Lou H. (2022). A Series of New Pyrrole Alkaloids with ALR2 Inhibitory Activities from the Sponge *Stylissa massa*. Mar. Drugs.

[190] Chang T.S., Lin J.J., Cheng K.C., Su J.H., She Y.Y., Wu Y.J. (2023). 11-*epi*-Sinulariolide Acetate-induced Apoptosis in Oral Cancer Cells Is Regulated by FOXO Through Inhibition of PI3K/AKT Pathway. Anticancer Res..

[191] Song Y., Tan Y., She J., Chen C., Wang J., Hu Y., Pang X., Liu Y. (2023). Tanzawaic Acid Derivatives from the Marine-Derived *Penicillium steckii* as Inhibitors of RANKL-Induced Osteoclastogenesis. J. Nat. Prod..

[192] Chang C.H., Lin B.J., Chen C.H., Nguyen N.L., Hsieh T.H., Su J.H., Chen M.C. (2023). Stellettin B Induces Cell Death in Bladder Cancer Via Activating the Autophagy/DAPK2/Apoptosis Signaling Cascade. Mar. Drugs.

[193] Sun C., Liu Q., Shah M., Che Q., Zhang G., Zhu T., Zhou J., Rong X., Li D. (2022). Talaverrucin A, Heterodimeric Oxaphenalenone from Antarctica Sponge-Derived Fungus *Talaromyces* sp. HDN151403, Inhibits Wnt/β-Catenin Signaling Pathway. Org. Lett..

[194] Su Y., Chen S., Liu S., Wang Y., Chen X., Xu M., Cai S., Pan N., Qiao K., Chen B. (2023). Affinity Purification and Molecular Characterization of Angiotensin-Converting Enzyme (ACE)-Inhibitory Peptides from *Takifugu flavidus*. Mar. Drugs.

[195] Wang Q., Chen D., Wang Y., Dong C., Liu J., Chen K., Song F., Wang C., Yuan J., Davis R.A. (2022). Thiaplakortone B attenuates RANKL-induced NF-κB and MAPK signaling and dampens OVX-induced bone loss in mice. Biomed. Pharmacother..

[196] Hao M.J., Chen P.N., Li H.J., Wu F., Zhang G.Y., Shao Z.Z., Liu X.P., Ma W.Z., Xu J., Mahmud T. (2022). β-Carboline Alkaloids From the Deep-Sea Fungus *Trichoderma* sp. MCCC 3A01244 as a New Type of Anti-pulmonary Fibrosis Agent that Inhibits TGF-β/Smad Signaling Pathway. Front. Microbiol..

[197] Kim A.T., Park Y. (2023). Trifuhalol A, a phlorotannin from the brown algae *Agarum cribosum*, reduces adipogenesis of human primary adipocytes through Wnt/β-catenin and AMPK-dependent pathways. Curr. Res. Food Sci..

[198] Yang J., Li J., Wei T.T., Pang J.Y., Du Y.H. (2023). Marine Compound Exerts Antiaging Effect in Human Endothelial Progenitor Cells via Increasing Sirtuin 1 Expression. ACS Pharmacol. Transl. Sci..

[199] Tong Y., Zhu W., Wen T., Mukhamejanova Z., Xu F., Xiang Q., Pang J. (2022). Xyloketal B Reverses Nutritional Hepatic Steatosis, Steatohepatitis, and Liver Fibrosis through Activation of the PPARα/PGC1α Signaling Pathway. J. Nat. Prod..

[200] Carroll A.R., Copp B.R., Davis R.A., Keyzers R.A., Prinsep M.R. (2022). Marine natural products. Nat. Prod. Rep..

[201] Carroll A.R., Copp B.R., Davis R.A., Keyzers R.A., Prinsep M.R. (2023). Marine natural products. Nat. Prod. Rep..

[202] Dembitsky V.M. (2023). Fascinating Furanosteroids and Their Pharmacological Profile. Molecules.

[203] Dembitsky V.M. (2022). Natural Polyether Ionophores and Their Pharmacological Profile. Mar. Drugs.

[204] Guo F.W., Zhang Q., Gu Y.C., Shao C.L. (2023). Sulfur-containing marine natural products as leads for drug discovery and development. Curr. Opin. Chem. Biol..

[205] Pereira L., Cotas J. (2023). Therapeutic Potential of Polyphenols and Other Micronutrients of Marine Origin. Mar. Drugs.

[206] Tan L.T. (2023). Impact of Marine Chemical Ecology Research on the Discovery and Development of New Pharmaceuticals. Mar. Drugs.

[207] Swain S., Bej S., Bishoyi A.K., Mandhata C.P., Sahoo C.R., Padhy R.N. (2023). Recent progression on phytochemicals and pharmacological properties of the filamentous cyanobacterium *Lyngbya* sp.. Naunyn-Schmiedeberg’s Arch. Pharmacol..

[208] Nawaz T.A., Gu L., Fahad S., Saud S., Jiang Z., Hassan S., Harrison M.T., Liu K., Khan M.A., Liu H. (2023). A comprehensive review of the therapeutic potential of cyanobacterial marine bioactives: Unveiling the hidden treasures of the sea. Food Energy Secur..

[209] Perera R.M.T.D., Herath K.H.I.N., Sanjeewa K.K.A., Jayawardena T.U. (2023). Recent Reports on Bioactive Compounds from Marine Cyanobacteria in Relation to Human Health Applications. Life.

[210] Moopantakath J., Imchen M., Anju V.T., Busi S., Dyavaiah M., Martínez-Espinosa R.M., Kumavath R. (2023). Bioactive molecules from haloarchaea: Scope and prospects for industrial and therapeutic applications. Front. Microbiol..

[211] Besednova N.N., Andryukov B.G., Zaporozhets T.S., Kuznetsova T.A., Kryzhanovsky S.P., Ermakova S.P., Galkina I.V., Shchelkanov M.Y. (2022). Molecular Targets of Brown Algae Phlorotannins for the Therapy of Inflammatory Processes of Various Origins. Mar. Drugs.

[212] Meinita M.D.N., Harwanto D., Amron, Hannan M.A., Jeong G., Moon I.S., Choi J.S. (2023). A concise review of the potential utilization based on bioactivity and pharmacological properties of the genus *Gelidium* (Gelidiales, Rhodophyta). J. Appl. Phycol..

[213] Wang X., Huang C., Fu X., Jeon Y.J., Mao X., Wang L. (2023). Bioactivities of the Popular Edible Brown Seaweed *Sargassum fusiforme*: A Review. J. Agric. Food Chem..

[214] Dai N., Wang Q., Xu B., Chen H. (2022). Remarkable natural biological resource of algae for medical applications. Front. Mar. Sci..

[215] El-Sheekh M., Fathy A.A., Saber H., Saaber A.A. (2023). Medicinal and Pharmaceutical Applications of Seaweeds. Egypt. J. Bot..

[216] Georgii A.D.N.P., Teixeira V.L. (2023). Dictyota and Canistrocarpus Brazilian Brown Algae and Their Bioactive Diterpenes—A Review. Mar. Drugs.

[217] Ibrahim T.N.B.T., Feisal N.A.S., Kamaludin N.H., Cheah W.Y., How V., Bhatnagar A., Ma Z., Show P.L. (2023). Biological active metabolites from microalgae for healthcare and pharmaceutical industries: A comprehensive review. Bioresour. Technol..

[218] Yang Y., Hassan S.H., Awasthi M.K., Gajendran B., Sharma M., Ji M., Salama E. (2023). The recent progress on the bioactive compounds from algal biomass for human health applications. Food Biosci..

[219] Sadeer N.B., Zengin G., Mahomoodally M.F. (2023). Biotechnological applications of mangrove plants and their isolated compounds in medicine-a mechanistic overview. Crit. Rev. Biotechnol..

[220] Chen Y., Xu L.C., Liu S., Zhang Z.X., Cao G.Y. (2022). Halometabolites isolated from the marine-derived fungi with potent pharmacological activities. Front. Microbiol..

[221] Gao L.-W., Zhang P. (2023). An update on chemistry and bioactivities of secondary metabolites from the marine algal-derived endophytic fungi. Phytochem. Rev..

[222] Hafez Ghoran S., Taktaz F., Sousa E., Fernandes C., Kijjoa A. (2023). Peptides from Marine-Derived Fungi: Chemistry and Biological Activities. Mar. Drugs.

[223] Wang Z., Qader M., Wang Y., Kong F., Wang Q., Wang C. (2023). Progress in the discovery of new bioactive substances from deep-sea associated fungi during 2020–2022. Front. Mar. Sci..

[224] Cooreman K., De Spiegeleer B., Van Poucke C., Vanavermaete D., Delbare D., Wynendaele E., De Witte B. (2023). Emerging pharmaceutical therapies of Ascidian-derived natural products and derivatives. Environ. Toxicol. Pharmacol..

[225] Chen Z.H., Guo Y.W., Li X.W. (2023). Recent advances on marine mollusk-derived natural products: Chemistry, chemical ecology and therapeutical potential. Nat. Prod. Rep..

[226] Hadisaputri Y.E., Nurhaniefah A.A., Sukmara S., Zuhrotun A., Hendriani R., Sopyan I. (2023). *Callyspongia* spp.: Secondary Metabolites, Pharmacological Activities, and Mechanisms. Metabolites.

[227] Liang J., She J., Fu J., Wang J., Ye Y., Yang B., Liu Y., Zhou X., Tao H. (2023). Advances in Natural Products from the Marine-Sponge-Associated Microorganisms with Antimicrobial Activity in the Last Decade. Mar. Drugs.

[228] Fagbohun O.F., Joseph J.S., Oriyomi O.V., Rupasinghe H.P.V. (2023). Saponins of North Atlantic Sea Cucumber: Chemistry, Health Benefits, and Future Prospectives. Mar. Drugs.

[229] Davies-Coleman M.T., McPhail K.L., Parker-Nance S. (2023). A Quarter Century of Marine Biodiscovery in Algoa Bay, South Africa. J. Nat. Prod..

[230] Sala S., Micke S.K., Flematti G.R. (2023). Marine Natural Products from Flora and Fauna of the Western Australian Coast: Taxonomy, Isolation and Biological Activity. Molecules.

[231] Martínez-Fructuoso L., Arends S.J.R., Freire V.F., Evans J.R., DeVries S., Peyser B.D., Akee R.K., Thornburg C.C., Kumar R., Ensel S. (2023). Screen for New Antimicrobial Natural Products from the NCI Program for Natural Product Discovery Prefractionated Extract Library. ACS Infect. Dis..

[232] Chukwudulue U.M., Barger N., Dubovis M., Luzzatto Knaan T. (2023). Natural Products and Pharmacological Properties of Symbiotic Bacillota (Firmicutes) of Marine Macroalgae. Mar. Drugs.

[233] Eze O.C., Berebon D.P., Emencheta S.C., Evurani S.A., Okorie C.N., Balcão V.M., Vila M.M.D.C. (2023). Therapeutic Potential of Marine Probiotics: A Survey on the Anticancer and Antibacterial Effects of *Pseudoalteromonas* spp.. Pharmaceuticals.

[234] Devkar H.U., Thakur N.L., Kaur P. (2023). Marine-derived antimicrobial molecules from the sponges and their associated bacteria. Can. J. Microbiol..

[235] Li H., Fu Y., Song F., Xu X. (2023). Recent Updates on the Antimicrobial Compounds from Marine-Derived *Penicillium* fungi. Chem. Biodivers..

[236] Guryanova S.V., Balandin S.V., Belogurova-Ovchinnikova O.Y., Ovchinnikova T.V. (2023). Marine Invertebrate Antimicrobial Peptides and Their Potential as Novel Peptide Antibiotics. Mar. Drugs.

[237] Zhang Y., Lin M., Qin Y., Lu H., Xu X., Gao C., Liu Y., Luo W., Luo X. (2023). Anti-Vibrio potential of natural products from marine microorganisms. Eur. J. Med. Chem..

[238] Awdhesh Kumar Mishra R., Kodiveri Muthukaliannan G. (2022). Role of microalgal metabolites in controlling quorum-sensing-regulated biofilm. Arch. Microbiol..

[239] Wibowo J.T., Bayu A., Aryati W.D., Fernandes C., Yanuar A., Kijjoa A., Putra M.Y. (2023). Secondary Metabolites from Marine-Derived Bacteria with Antibiotic and Antibiofilm Activities against Drug-Resistant Pathogens. Mar. Drugs.

[240] Khan F., Tabassum N., Bamunuarachchi N.I., Kim Y.M. (2022). Phloroglucinol and Its Derivatives: Antimicrobial Properties toward Microbial Pathogens. J. Agric. Food Chem..

[241] Thawabteh A.M., Swaileh Z., Ammar M., Jaghama W., Yousef M., Karaman R., Bufo S.A., Scrano L. (2023). Antifungal and Antibacterial Activities of Isolated Marine Compounds. Toxins.

[242] Ngo-Mback M.N.L., Zeuko’o Menkem E., Marco H.G. (2023). Antifungal Compounds from Microbial Symbionts Associated with Aquatic Animals and Cellular Targets: A Review. Pathogens.

[243] Ganeshkumar A., Gonçale J.C., Rajaram R., Junqueira J.C. (2023). Anti-Candidal Marine Natural Products: A Review. J. Fungi.

[244] Deshmukh S.K., Agrawal S., Gupta M.K., Patidar R.K., Ranjan N. (2022). Recent Advances in the Discovery of Antiviral Metabolites from Fungi. Curr. Pharm. Biotechnol..

[245] Singh U., Gandhi H.A., Nikita, Bhattacharya J., Tandon R., Tiwari G.L. (2023). Cyanometabolites: Molecules with immense antiviral potential. Arch. Microbiol..

[246] Fagundes T., Vasconcelos T.R., dos Santos Junior F., Rajsfus B.F., Allonso D., Menezes J.C., Valverde A.I., Campos V.R. (2023). Marine Natural Products in the Battle against Dengue, Zika, and Chikungunya Arboviruses. J. Braz. Chem. Soc..

[247] Gunathilaka M.D.T.L. (2023). Utilization of Marine Seaweeds as a Promising Defense Against COVID-19: A Mini-review. Mar. Biotechnol..

[248] Pereira R.S., Santos F.C.P., Campana P.R.V., Costa V.V., de Pádua R.M., Souza D.G., Teixeira M.M., Braga F.C. (2023). Natural Products and Derivatives as Potential *Zika* virus Inhibitors: A Comprehensive Review. Viruses.

[249] Pérez-Vargas J., Shapira T., Olmstead A.D., Villanueva I., Thompson C.A.H., Ennis S., Gao G., De Guzman J., Williams D.E., Wang M. (2023). Discovery of lead natural products for developing pan-SARS-CoV-2 therapeutics. Antivir. Res..

[250] Estrella-Parra E.A., Arreola R., Álvarez-Sánchez M.E., Torres-Romero J.C., Rojas-Espinosa O., De la Cruz-Santiago J.A., Martinez-Benitez M.B., López-Camarillo C., Lara-Riegos J.C., Arana-Argáez V.E. (2022). Natural marine products as antiprotozoal agents against amitochondrial parasites. Int. J. Parasitol. Drugs Drug Resist..

[251] Ribeiro R., Costa L., Pinto E., Sousa E., Fernandes C. (2023). Therapeutic Potential of Marine-Derived Cyclic Peptides as Antiparasitic Agents. Mar. Drugs.

[252] Zhang M., Zhang Q., Cui X., Zhu L. (2023). Promising Antiparasitic Natural and Synthetic Products from Marine Invertebrates and Microorganisms. Mar. Drugs.

[253] Hou A., Li B., Deng Z., Xu M., Dickschat J.S. (2023). Cladosporin, A Highly Potent Antimalaria Drug?. Chembiochem.

[254] Negm W.A., Ezzat S.M., Zayed A. (2023). Marine organisms as potential sources of natural products for the prevention and treatment of malaria. RSC Adv..

[255] Chen N., Zhang S., Javeed A., Jian C., Liu Y., Sun J., Wu S., Fu P., Han B. (2023). Structures and Anti-Allergic Activities of Natural Products from Marine Organisms. Mar. Drugs.

[256] Khursheed M., Ghelani H., Jan R.K., Adrian T.E. (2023). Anti-Inflammatory Effects of Bioactive Compounds from Seaweeds, Bryozoans, Jellyfish, Shellfish and Peanut Worms. Mar. Drugs.

[257] Couttolenc A., Medina M.E., Trigos A., Espinoza C. (2022). Antioxidant capacity of fungi associated with corals and sponges of the reef system of Veracruz, Mexico. Electron. J. Biotechnol..

[258] Martignago C.C.S., Soares-Silva B., Parisi J.R., Silva L.C.S.E., Granito R.N., Ribeiro A.M., Renno A.C.M., de Sousa L.R.F., Aguiar A.C.C. (2023). Terpenes extracted from marine sponges with antioxidant activity: A systematic review. Nat. Prod. Bioprospect..

[259] Nguyen N.B.A., El-Shazly M., Chen P.J., Peng B.R., Chen L.Y., Hwang T.L., Lai K.H. (2023). Unlocking the Potential of Octocoral-Derived Secondary Metabolites against Neutrophilic Inflammatory Response. Mar. Drugs.

[260] Akram W., Rihan M., Ahmed S., Arora S., Ahmad S., Vashishth R. (2023). Marine-Derived Compounds Applied in Cardiovascular Diseases: Submerged Medicinal Industry. Mar. Drugs.

[261] Islam M.R., Dhar P.S., Akash S., Syed S.H., Gupta J.K., Gandla K., Akter M., Rauf A., Hemeg H.A., Anwar Y. (2023). Bioactive molecules from terrestrial and seafood resources in hypertension treatment: Focus on molecular mechanisms and targeted therapies. Nat. Prod. Bioprospect..

[262] Liu L., Chen Y., Chen B., Xu M., Liu S., Su Y., Qiao K., Liu Z. (2023). Advances in Research on Marine-Derived Lipid-Lowering Active Substances and Their Molecular Mechanisms. Nutrients.

[263] Caffrey C., Leamy A., O’Sullivan E., Zabetakis I., Lordan R., Nasopoulou C. (2023). Cardiovascular Diseases and Marine Oils: A Focus on Omega-3 Polyunsaturated Fatty Acids and Polar Lipids. Mar. Drugs.

[264] Cho C. (2022). Therapeutic potential of seaweed-derived bioactive compounds for cardiovascular disease treatment. Appl. Sci..

[265] Chellappan D.K., Chellian J., Rahmah N.S.N., Gan W.J., Banerjee P., Sanyal S., Ghosh N., Guith T., Das A., Gupta G. (2023). Hypoglycaemic Molecules for the Management of Diabetes Mellitus from Marine Sources. Diabetes Metab. Syndr. Obes..

[266] Agarwal S. (2023). Antidiabetic Potential of Seaweed and Their Bioactive Compounds: A Review of Developments in Last Decade. Crit. Rev. Food Sci. Nutr..

[267] Imchen T. (2023). Marine algae colorants: Antioxidant, anti-diabetic properties and applications in food industry. Algal Res..

[268] Kaushik A. (2023). Algal metabolites: Paving the way towards new generation antidiabetic therapeutics. Algal Res..

[269] Magwaza S.N., Islam M.S. (2023). Roles of Marine Macroalgae or Seaweeds and Their Bioactive Compounds in Combating Overweight, Obesity and Diabetes: A Comprehensive Review. Mar. Drugs.

[270] Pereira L., Valado A. (2023). Algae-Derived Natural Products in Diabetes and Its Complications-Current Advances and Future Prospects. Life.

[271] Tamel Selvan K., Goon J.A., Makpol S., Tan J.K. (2023). Therapeutic Potentials of Microalgae and Their Bioactive Compounds on Diabetes Mellitus. Mar. Drugs.

[272] Casertano M., Vito A., Aiello A., Imperatore C., Menna M. (2023). Natural Bioactive Compounds from Marine Invertebrates That Modulate Key Targets Implicated in the Onset of Type 2 Diabetes Mellitus (T2DM) and Its Complications. Pharmaceutics.

[273] Tian Z., Lu X.T., Jiang X., Tian J. (2023). Bryostatin-1: A promising compound for neurological disorders. Front. Pharmacol..

[274] Rivai B. (2023). Neuroprotective compounds from marine invertebrates. Beni Suef Univ. J. Basic. Appl. Sci..

[275] Wang Z.L., Zhang S.Y., Hao S.L., Yang W.X. (2023). Neurotoxins and pore forming toxins in sea anemones: Potential candidates for new drug development. Histol. Histopathol..

[276] Medoro A., Davinelli S., Milella L., Willcox B.J., Allsopp R.C., Scapagnini G., Willcox D.C. (2023). Dietary Astaxanthin: A Promising Antioxidant and Anti-Inflammatory Agent for Brain Aging and Adult Neurogenesis. Mar. Drugs.

[277] Diao X., Han H., Li B., Guo Z., Fu J., Wu W. (2023). The Rare Marine Bioactive Compounds in Neurological Disorders and Diseases: Is the Blood-Brain Barrier an Obstacle or a Target?. Mar. Drugs.

[278] Kwon Y.J., Kwon O.I., Hwang H.J., Shin H.C., Yang S. (2023). Therapeutic effects of phlorotannins in the treatment of neurodegenerative disorders. Front. Mol. Neurosci..

[279] Khairinisa M.A., Latarissa I.R., Athaya N.S., Charlie V., Musyaffa H.A., Prasedya E.S., Puspitasari I.M. (2023). Potential Application of Marine Algae and Their Bioactive Metabolites in Brain Disease Treatment: Pharmacognosy and Pharmacology Insights for Therapeutic Advances. Brain Sci..

[280] Deepika N.P., Rahman M.H., Chipurupalli S., Shilpa T.N., Duraiswamy B. (2023). The Emerging Role of Marine Natural Products for the Treatment of Parkinson’s Disease. CNS Neurol. Disord. Drug Targets.

[281] Silva J., Alves C., Soledade F., Martins A., Pinteus S., Gaspar H., Alfonso A., Pedrosa R. (2023). Marine-Derived Components: Can They Be a Potential Therapeutic Approach to Parkinson’s Disease?. Mar. Drugs.

[282] Hu D., Jin Y., Hou X., Zhu Y., Chen D., Tai J., Chen Q., Shi C., Ye J., Wu M. (2023). Application of Marine Natural Products against Alzheimer’s Disease: Past, Present and Future. Mar. Drugs.

[283] Brillante S., Galasso C., Lauritano C., Carrella S. (2022). From the Sea for the Sight: Marine Derived Products for Human Vision. Front. Aging Neurosci..

[284] Fiorotti H.B., Figueiredo S.G., Campos F.V., Pimenta D.C. (2023). Cone snail species off the Brazilian coast and their venoms: A review and update. J. Venom. Anim. Toxins Incl. Trop. Dis..

[285] Groome J.R. (2023). Historical Perspective of the Characterization of Conotoxins Targeting Voltage-Gated Sodium Channels. Mar. Drugs.

[286] Nguyen L.T.T., Craik D.J., Kaas Q. (2023). Bibliometric Review of the Literature on Cone Snail Peptide Toxins from 2000 to 2022. Mar. Drugs.

[287] Huerta M., de la Nava J., Artacho-Cordón A., Nieto F.R. (2023). Efficacy and Security of Tetrodotoxin in the Treatment of Cancer-Related Pain: Systematic Review and Meta-Analysis. Mar. Drugs.

[288] Yang H., Zhang Q., Zhang B., Zhao Y., Wang N. (2023). Potential Active Marine Peptides as Anti-Aging Drugs or Drug Candidates. Mar. Drugs.

[289] Yamashita S., Miyazawa T., Higuchi O., Kinoshita M. (2023). Marine Plasmalogens: A Gift from the Sea with Benefits for Age-Associated Diseases. Molecules.

[290] Therdyothin A., Phiphopthatsanee N., Isanejad M. (2023). The Effect of Omega-3 Fatty Acids on Sarcopenia: Mechanism of Action and Potential Efficacy. Mar. Drugs.

[291] Labes A. (2023). Marine Resources Offer New Compounds and Strategies for the Treatment of Skin and Soft Tissue Infections. Mar. Drugs.

[292] Gago F. (2023). Computational Approaches to Enzyme Inhibition by Marine Natural Products in the Search for New Drugs. Mar. Drugs.

[293] Davinelli S., Saso L., D’Angeli F., Calabrese V., Intrieri M., Scapagnini G. (2022). Astaxanthin as a Modulator of Nrf2, NF-κB, and Their Crosstalk: Molecular Mechanisms and Possible Clinical Applications. Molecules.

[294] Nair A., Ahirwar A., Singh S., Lodhi R., Lodhi A., Rai A., Jadhav D.A., Harish, Varjani S., Singh G. (2023). Astaxanthin as a King of Ketocarotenoids: Structure, Synthesis, Accumulation, Bioavailability and Antioxidant Properties. Mar. Drugs.

[295] Deau E., Lindberg M.F., Miege F., Roche D., George N., George P., Krämer A., Knapp S., Meijer L. (2023). Leucettinibs, a Class of DYRK/CLK Kinase Inhibitors Inspired by the Marine Sponge Natural Product Leucettamine B. J. Med. Chem..

[296] Pascual Alonso I., Almeida García F., Valdés Tresanco M.E., Arrebola Sánchez Y., Ojeda Del Sol D., Sánchez Ramírez B., Florent I., Schmitt M., Avilés F.X. (2023). Marine Invertebrates: A Promissory Still Unexplored Source of Inhibitors of Biomedically Relevant Metallo Aminopeptidases Belonging to the M1 and M17 Families. Mar. Drugs.

[297] Costa A.M. (2023). Actin-Interacting Amphidinolides: Syntheses and Mechanisms of Action of Amphidinolides X, J, and K. Molecules.

[298] Córdova-Isaza A., Jiménez-Mármol S., Guerra Y., Salas-Sarduy E. (2023). Enzyme Inhibitors from Gorgonians and Soft Corals. Mar. Drugs.

[299] D’Aniello E., Amodeo P., Vitale R.M. (2023). Marine Natural and Nature-Inspired Compounds Targeting Peroxisome Proliferator Activated Receptors (PPARs). Mar. Drugs.

[300] Mir R.H., Mir P.A., Uppal J., Chawla A., Patel M., Bardakci F., Adnan M., Mohi-Ud-Din R. (2023). Evolution of Natural Product Scaffolds as Potential Proteasome Inhibitors in Developing Cancer Therapeutics. Metabolites.

[301] Ye W., Lui S.T., Zhao Q., Wong Y.M., Cheng A., Sung H.H., Williams I.D., Qian P.Y., Huang P. (2023). Novel marine natural products as effective TRPV1 channel blockers. Int. J. Biol. Macromol..

[302] Gonçalves A., Fernandes M., Lima M., Gomes J.P., Silva F., Castro S., Sampaio F., Gomes A.C. (2023). Nanotechnology to the Rescue: Therapeutic Strategies Based on Brown Algae for Neurodegenerative Diseases. Appl. Sci..

[303] Flores-Holguín N., Salas-Leiva J.S., Núñez-Vázquez E.J., Tovar-Ramírez D., Glossman-Mitnik D. (2023). Exploring marine toxins: Comparative analysis of chemical reactivity properties and potential for drug discovery. Front. Chem..

